# Commonality and variation in mental representations of music revealed by a cross-cultural comparison of rhythm priors in 15 countries

**DOI:** 10.1038/s41562-023-01800-9

**Published:** 2024-03-04

**Authors:** Nori Jacoby, Rainer Polak, Jessica A. Grahn, Daniel J. Cameron, Kyung Myun Lee, Ricardo Godoy, Eduardo A. Undurraga, Tomás Huanca, Timon Thalwitzer, Noumouké Doumbia, Daniel Goldberg, Elizabeth H. Margulis, Patrick C. M. Wong, Luis Jure, Martín Rocamora, Shinya Fujii, Patrick E. Savage, Jun Ajimi, Rei Konno, Sho Oishi, Kelly Jakubowski, Andre Holzapfel, Esra Mungan, Ece Kaya, Preeti Rao, Mattur A. Rohit, Suvarna Alladi, Bronwyn Tarr, Manuel Anglada-Tort, Peter M. C. Harrison, Malinda J. McPherson, Sophie Dolan, Alex Durango, Josh H. McDermott

**Affiliations:** 1 Computational Auditory Perception Group, Max Planck Institute for Empirical Aesthetics, Frankfurt am Main, Germany; 2 Presidential Scholars in Society and Neuroscience, Columbia University, New York, NY USA; 3 RITMO Centre for Interdisciplinary Studies in Rhythm, Time and Motion, University of Oslo, Blindern, Oslo, Norway; 4 Brain and Mind Institute and Department of Psychology, University of Western Ontario, London, Ontario Canada; 5 Department of Psychology, Neuroscience and Behaviour, McMaster University, Hamilton, Ontario Canada; 6 School of Digital Humanities and Social Sciences, Korea Advanced Institute of Science and Technology, Daejeon, Republic of Korea; 7 Graduate School of Culture Technology, Korea Advanced Institute of Science and Technology, Daejeon, Republic of Korea; 8 Heller School for Social Policy and Management, Brandeis University, Waltham, MA USA; 9 Escuela de Gobierno, Pontificia Universidad Católica de Chile, Santiago, Chile; 10 CIFAR Azrieli Global Scholars programme, CIFAR, Toronto, Ontario Canada; 11 Centro Boliviano de Investigación y Desarrollo Socio Integral, San Borja, Bolivia; 12 Department of Musicology, University of Vienna, Vienna, Austria; 13 Sciences de l’Education, Université Catholique d’Afrique de l’Ouest, Bamako, Mali; 14 Department of Music, University of Connecticut, Storrs, CT USA; 15 Department of Music, Princeton University, Princeton, NJ USA; 16 Department of Linguistics & Modern Languages and Brain and Mind Institute, Chinese University of Hong Kong, Hong Kong SAR, China; 17 School of Music, Universidad de la República, Montevideo, Uruguay; 18 Signal Processing Department, School of Engineering, Universidad de la República, Montevideo, Uruguay; 19 Music Technology Group, Universitat Pompeu Fabra, Barcelona, Spain; 20 Faculty of Environment and Information Studies, Keio University, Fujisawa, Japan; 21 School of Psychology, University of Auckland, Auckland, New Zealand; 22 Department of Traditional Japanese Music, Tokyo University of the Arts, Tokyo, Japan; 23 Department of Music, Durham University, Durham, UK; 24 Division of Media Technology and Interaction Design, KTH Royal Institute of Technology, Stockholm, Sweden; 25 Department of Psychology, Bogazici University, Istanbul, Turkey; 26 Max Planck Research Group ‘Neural and Environmental Rhythms’, Max Planck Institute for Empirical Aesthetics, Frankfurt am Main, Germany; 27 Cognitive Science Master Program, Bogazici University, Istanbul, Turkey; 28 Department of Electrical Engineering, Indian Institute of Technology Bombay, Mumbai, India; 29 Nizam’s Institute of Medical Sciences, Hyderabad, India; 30 Department of Cognitive and Evolutionary Anthropology, University of Oxford, Oxford, UK; 31 Department of Experimental Psychology, University of Oxford, Oxford, UK; 32 Department of Psychology, Goldsmiths, University of London, London, UK; 33 Faculty of Music, University of Cambridge, Cambridge, UK; 34 Department of Brain and Cognitive Sciences, Massachusetts Institute of Technology, Cambridge, MA USA; 35 Program in Speech and Hearing Biosciences and Technology, Harvard University, Cambridge, MA USA; 36 McGovern Institute for Brain Research, Massachusetts Institute of Technology, Cambridge, MA USA; 37 Department of Brain and Cognitive Sciences, Wellesley College, Wellesley, MA USA; 38 Neurosciences Graduate Program, Stanford University, Stanford, CA USA; 39 Center for Brains, Minds & Machines, Massachusetts Institute of Technology, Cambridge, MA USA

**Keywords:** Human behaviour, Human behaviour

## Abstract

Music is present in every known society but varies from place to place. What, if anything, is universal to music cognition? We measured a signature of mental representations of rhythm in 39 participant groups in 15 countries, spanning urban societies and Indigenous populations. Listeners reproduced random ‘seed’ rhythms; their reproductions were fed back as the stimulus (as in the game of ‘telephone’), such that their biases (the prior) could be estimated from the distribution of reproductions. Every tested group showed a sparse prior with peaks at integer-ratio rhythms. However, the importance of different integer ratios varied across groups, often reflecting local musical practices. Our results suggest a common feature of music cognition: discrete rhythm ‘categories’ at small-integer ratios. These discrete representations plausibly stabilize musical systems in the face of cultural transmission but interact with culture-specific traditions to yield the diversity that is evident when mental representations are probed across many cultures.

## Abstract (web summary)

Measuring rhythm priors in 39 participant groups from 15 countries, the authors find that properties of rhythm representations are common across cultures, while variation from place to place related to local musical traditions exists.

## Main

Music, like language, is conceived by Western scholars to consist of combinations of discrete elements^1^. Musical notes are grouped into phrases and described in terms of discrete intervals in frequency and time. In Western music, these intervals are non-arbitrary, often defined by integer ratios between frequencies or durations. Although music is notated in terms of these intervals, actual musical performances can deviate considerably from notated intervals in frequency and time^2–6^. Discrete symbolic mental representations of music are thought to be aided by categorical perception^7–11^—the perceptual mapping of continuous spaces of signals onto discrete elements^12^. Yet most studies of music perception have been conducted on listeners in Western Europe or North America, leaving the cross-cultural generality of such discrete representations unclear.

Attempts to characterize music from around the world have supported the idea that there are universal properties of music, including a reliance on discrete elements defined by simple-integer ratios^13–17^. However, such analyses of musical corpora have largely relied on Western-trained researchers to annotate what they hear when listening to recordings from other cultures, with the unavoidable possibility that researchers’ perceptual biases influence the results. Experiments to assess mental representations across cultures could more definitively address commonalities and variation in music cognition but have thus far been limited to small numbers of societies^18–31^ or to participants on the internet^32,33^, who plausibly have extensive exposure to a distribution of music similar to that consumed by typical participants in Western Europe and North America. In addition, experiments in non-musicians in the United States have in some cases failed to find evidence for discrete musical features such as pitch intervals and chords^34,35^. The universality of psychological mechanisms supporting discrete representations of music has thus remained unclear, as has the extent to which such representations are biologically constrained to exhibit characteristics found in Western European and North American listeners.

Here we present a large-scale study of cross-cultural variation in music cognition, measuring signatures of discrete mental representations of rhythm in a large number of diverse participant groups around the world. We used a paradigm developed in an earlier study^26^ to characterize Bayesian priors on simple periodic rhythms, which in US participants exhibit discrete peaks (modes) at rhythms composed of time intervals related by ratios of small integers (‘simple’ integer ratios such as 1:2 or 2:3 as opposed to 4:7 or 7:12)^36^. These modes bias the internal representation of presented rhythms by shifting them towards the rhythm represented by a mode, causing rhythms to be perceived categorically. We sought to assess the prevalence of this phenomenon in Western and non-Western listeners, as well as the relation of rhythm categories to culture-specific musical traditions. Throughout this paper, we at times use the term ‘Western’ as a summary term to refer to people from Western Europe or North America and ‘non-Western’ to refer to people from other geographical regions. We note that societies may differ on other dimensions as well (in particular, the Western societies we discuss are relatively educated, industrialized, rich and democratic^37^) and that there is a large degree of variability across Western societies and particularly within the residual category of non-Western societies. Our choice to contrast Western and non-Western listeners reflects the legacy of scholarship in this domain, which has often taken Western music as a reference point and has predominantly documented perception in Western listeners. Our use of this dichotomy should not be taken as an endorsement.

**Fig. 1:**
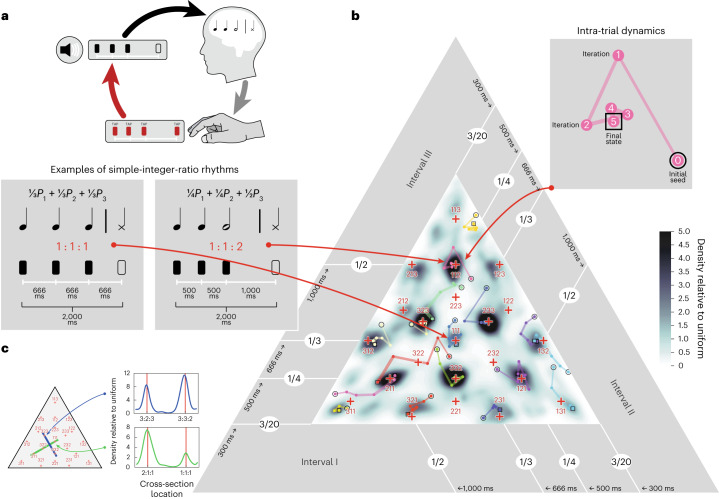
Iterated reproduction experiment paradigm and analysis. **a**, Schematic of the experiment. Participants are presented with initially random seed rhythms (a repeating cycle of three clicks, defined by three inter-click time intervals) and reproduce them by tapping. The reproduction becomes the stimulus on the following iteration. This procedure is iterated five times (one ‘trial’). Credit: Felix Bernoully. **b**, The triangular rhythm space in which the results are plotted (shown here for candombe musicians in Uruguay, for illustration purposes). Each axis (side) of the triangle represents one of the three intervals in a rhythm. We constrained each interval to be at least 300 ms in duration, and the total pattern duration to be 2,000 ms, resulting in the space within the inner triangle. Small-integer-ratio rhythms, in which the time intervals are related by ratios with integers less than 4 (Methods), occupy a subset of points in the triangular rhythm space. Two example small-integer-ratio rhythms (1:1:2 and 1:1:1) are shown to the left. For these examples, the three intervals are marked with white lines, the solid rectangles mark the three clicks of a cycle, and the outlined rectangle marks the click that starts the subsequent repeated cycle. The coloured dots connected by lines show trajectories from example experimental trials. The inset shows the dynamics of one example trial in more detail, converging in this case to the 1:1:2 rhythm. The distribution of reproductions is summarized with a kernel density estimate, plotted in greyscale over the rhythm triangle. **c**, Cross-sections through example modes in the prior shown in **b**, showing peaks at small-integer ratios; see also Extended Data Fig. 1. See Extended Data Fig. 2 and Supplementary Fig. 1 for the results of a second experiment conducted at a faster tempo by using a pattern duration of 1,000 ms.

In the experiment, individuals are initially presented with a random ‘seed’ rhythm: a repeating cycle of three clicks, separated by three successive time intervals, which we constrained to sum to two seconds. Participants reproduce the pattern by tapping along to it (Fig. 1a). Empirically, the reproduction is biased away from the actual stimulus rhythm, as might be expected if the internal representation of the rhythm were influenced by a prior via Bayesian inference. The reproduction is then substituted as the stimulus, and the process is iterated^26,38,39^. If the stimulus representation is determined by Bayesian inference with a fixed prior, the reproduction should be further biased at each iteration by the prior, such that it is eventually indistinguishable from a sample from the prior. The prior can then be estimated by running the procedure multiple times with different random seed rhythms. Here we used a kernel density estimate from the last iteration of a large set of random seeds to approximate the prior (Methods).

The results of the experiment are plotted in a triangular ‘rhythm space’ of three-interval rhythms (Fig. 1b). Each of the three axes of the rhythm space represents one of the three intervals. Because the total duration of the three-element rhythm is constrained to 2,000 ms, two of the intervals are sufficient to uniquely specify the rhythm. The brief and simple rhythms defined by this space are elements that can be composed (in accordance with other musical constraints) to form longer and more complex rhythms^8,27,40^. Small-integer-ratio rhythms are particular points in this space (Fig. 1b; the red crosses demarcate small-integer-ratio rhythms whose duration ratios are defined by integers less than 4), with isochrony (1:1:1) lying in the middle.

**Extended Data Fig. 1:**
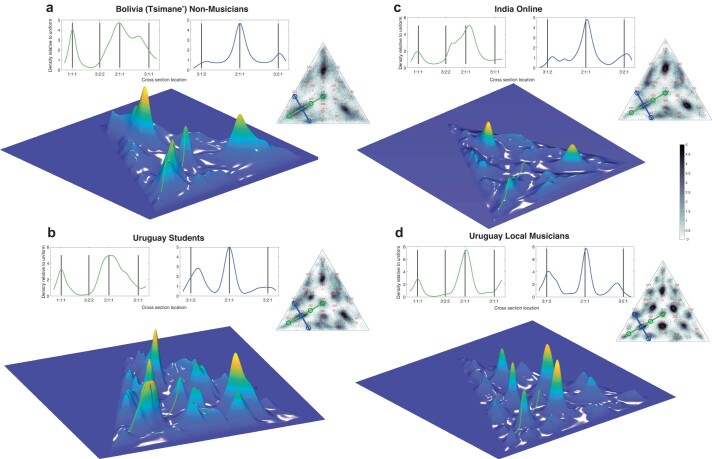
Alternative prior visualizations. Although some groups exhibited modes that were elongated more in some directions than others, all had clear peaks. The figure displays 2D and 3D plots of estimated priors from three groups that showed elongated modes (BO.TS, IN.OL, UY-ST), and as a comparison, one group with less elongated modes (UY-LM). For each group we draw two cross sections of an example mode from each group (shown in green and blue). **a**, Tsimane’ Non-Musicians. **b**, Uruguay-Students. **c**, India-Online. **d**, Brazil-Local Musicians. The cross sections show that the modes can be wider in one direction than the other, and also that the peaks fall on small-integer ratios. In the case of the Tsimane’ group, the mode elongation may be related to musical phrase elongation described in an early ethnographic study of Tsimane’ music. According to Riester (1975), free ‘fermata’ breaks usually occur near the end of phrases in Tsimane’ music. Example 104 in Riester’s 1975 treatise shows a break occurring after a long note preceded by a 1:1:2 pattern. This break produces a pattern in the direction of 1:1:3 (elongation of the longest tone), consistent with the elongation of 1:1:2 mode in this direction. This example provides additional evidence that the details of the priors revealed by our experiments relate to local music traditions (see also Fig. 7).

We previously found that iterated rhythm reproductions of US participants converged to stationary distributions, consistent with Bayesian inference under a prior. Moreover, the distributions for both musician and non-musician participants contained modes at small-integer-ratio rhythms^26^. Figure 1b shows example trajectories from an experiment in one group, which can be seen to gravitate towards small-integer ratios. The distribution of the reproductions can be estimated and visualized as kernel density estimates in the rhythm space (cross sections of the densities show that the densities have peaks near small-integer ratios; Fig. 1c; see also Extended Data Fig. 1).

Although the experiment results should in principle be influenced by motor noise, we found in practice that such noise is small enough to not substantially influence the results. In particular, the distributions for US musicians and non-musicians are similar despite much higher tapping precision for musicians^26^. Several additional strands of evidence support the idea that the distribution measured by the experiment reflects a perceptual prior^26^ despite being dependent on motor responses (‘Discussion’). We also note that the goals of the present paper do not hinge on whether the mental representation measured by the experiment is purely perceptual in origin. The experiment measures the biases in how a heard rhythm is translated into a reproduction, as would shape musical practice and its cultural transmission irrespective of whether these biases reside entirely in the realm of perception. We refer to the result of the experiment as a ‘prior’, cognizant that it could in principle be partly distinct from a classical perceptual prior.

**Fig. 2:**
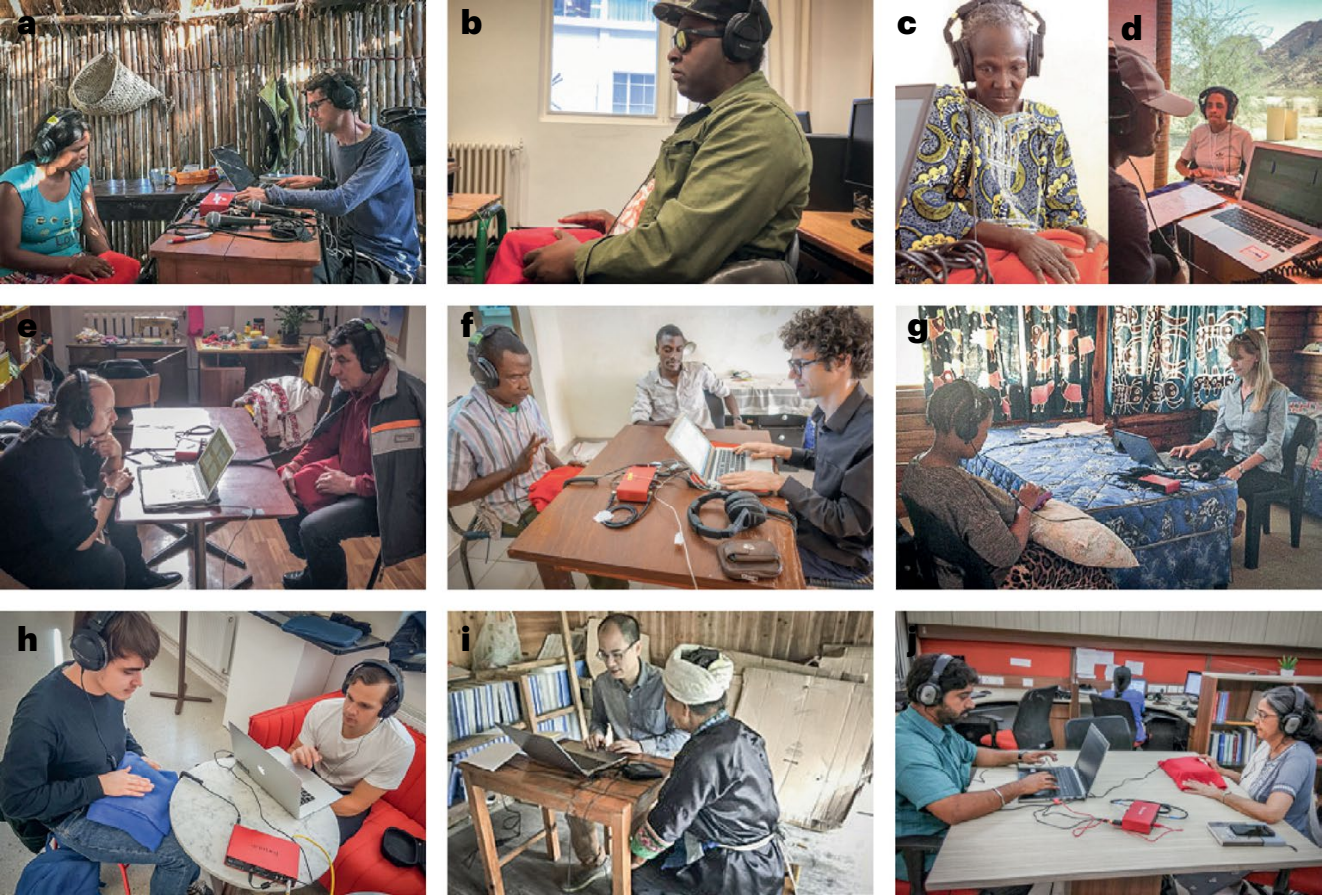
Example testing sites. **a**, Yaranda, Bolivia. **b**, Montevideo, Uruguay. **c**, Sagele, Mali. **d**, Spitzkoppe, Namibia. **e**, Pleven, Bulgaria. **f**, Bamako, Mali. **g**, D’Kar, Botswana. **h**, Stockholm, Sweden. **i**, Guizhou, China. **j**, Mumbai, India. Verbal informed consent was obtained from the individuals in each photo.

The iterated reproduction paradigm is well suited to cross-cultural experiments. The task is intuitive for participants, with minimal reliance on verbal instructions, and is easy to run in the field, allowing us to run the same experiment across many different groups speaking different languages and with varying levels of education (Fig. 2). The paradigm also has the attractive feature of being independent of any specific hypothesis about the structure of rhythm representation. In particular, the experiment is not limited to testing whether phenomena prominent in Western listeners (discrete modes at small-integer ratios) are present in other cultures. A group of people could in principle exhibit a uniform prior (without discrete modes), or one with a completely different modal structure from that observed in Westerners.

We previously observed a prior with small-integer-ratio modes in one Indigenous Amazonian society^26^. However, the integer ratios at which the modes occurred were different than in US participants. To test whether discrete modes at small-integer ratios are present across groups and to assess the nature and extent of cross-cultural variation, we ran the experiment in a large number of participant groups around the world.

## Results

**Fig. 3:**
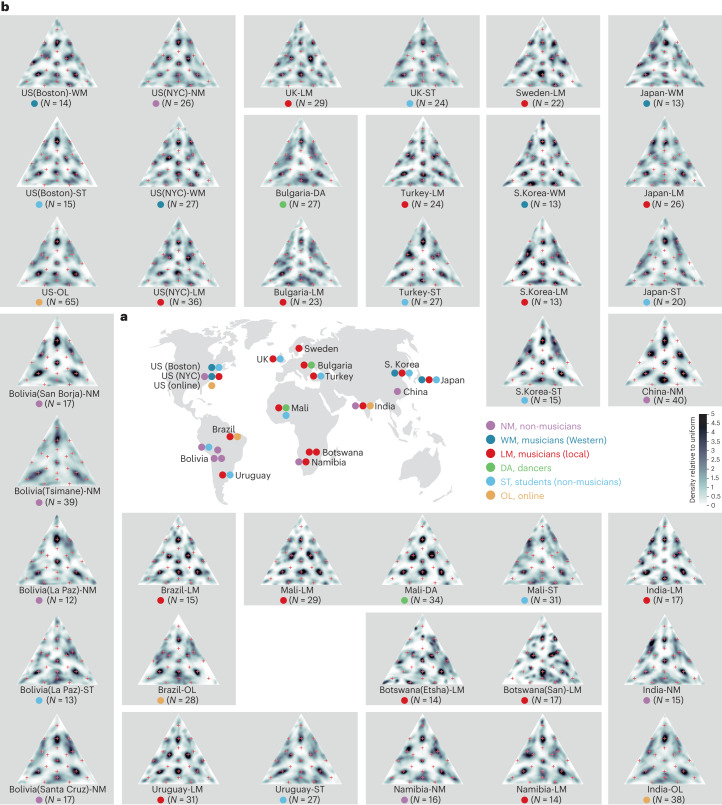
Summary results of iterated rhythm reproduction in 15 countries. **a**, Map of test sites. Credit: Felix Bernoully. **b**, Rhythm priors from all 39 groups tested. Musicians are labelled as ‘Western’ if they predominantly play Western music and ‘local’ if they predominantly play any other style. See Methods for the criteria used to classify participants as ‘musicians’ or ‘non-musicians’. The red crosses demarcate small-integer-ratio rhythms whose duration ratios are defined by integers less than or equal to 3. NM, non-musicians; WM, musicians (Western); LM, musicians (local); DA, dancers; ST, students (non-musicians); OL, online.

We tested 39 participant groups, spanning five continents and 15 countries (Fig. 3a; see Extended Data Table 1 for a summary of each group’s demographics). The groups were chosen primarily to provide a strong test of (1) potential universality by comparing groups with diverse musical experiences and (2) the role of musical experience in shaping mental representations. We thus included groups ranging from industrialized to small-scale societies, as well as groups of musicians and dancers from some non-Western societies. We also selected groups whose musical traditions were known to have distinct rhythmic characteristics to test whether any cross-cultural variability in mental representations could be explained by exposure to local musical styles. Where possible, we tested multiple groups from the same country that differed in the nature of their presumptive musical exposure. We also tested both university students and online participants in a number of countries. Compared with the other participant groups in the same countries, university students and online participants had musical experience that was more like that of typical Western participants; these participant groups were intended to assess the potential effects of exposure to Western/globalized music on mental representations of rhythm, and thus the consequences of reliance on student and online participants in cross-cultural research^37,41^. We chose participant groups on the basis of these criteria in conjunction with practical constraints (we tested more groups in some countries than in others primarily due to constraints of testing time and access to particular populations).

The key questions we sought to answer were (1) whether all groups would exhibit discrete rhythm categories; (2) whether any discrete categories would consistently occur at small-integer-ratio rhythms; (3) how rhythm categories would vary across groups, if at all; and (4) whether any cross-cultural variation would be related to musical or other demographic characteristics of the group.

### All groups exhibit priors with discrete modes

The measured priors are shown in Fig. 3b. Their most obvious feature is that they are non-uniform, being dominated in all cases by a set of relatively discrete modes that form local maxima in two dimensions. This need not have been the case—some or all priors could have been uniform or could have exhibited one-dimensional ridges rather than discrete two-dimensional modes. We substantiated this observation quantitatively in two ways. First, we found that in all 39 groups, 33% of the bins in the triangular rhythm space contained at least 61% of the rhythm reproductions in the fifth iteration (mean, 70.1%; range, 61.8–81.7%), suggesting that most of the probability mass is concentrated in a small portion of the space. The odds of this happening by chance are very low (*P* < 0.001 for points randomly positioned in the rhythm triangle, via bootstrapping). Second, the peak of the distribution in all 39 groups was at least five times larger than the uniform density (the ratio of the peak to the uniform ranged from 5.3 to 13.1, with a mean of 8.8; this ratio was significantly greater than would be expected by chance; *P* < 0.001 for all groups, via bootstrapping), suggesting that all distributions were far from uniform and very ‘peaky’. Although some groups have modes that are elongated more in one direction than another, all modes had clear peaks (evident in cross-sectional plots of the densities shown in Extended Data Fig. 1).

### All groups exhibit modes at small-integer-ratio rhythms

**Fig. 4:**
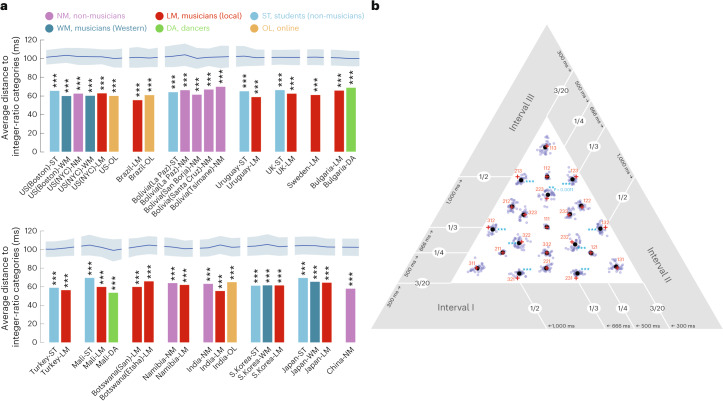
Relationship of priors to integer-ratios. **a**, Average distance from the nearest small-integer-ratio rhythm, for each participant group (split into two rows to make the figure more compact). This measure is small for a prior with all its mass at small-integer-ratio rhythms. The lines and shaded regions at the top plot the mean and 95% CI of a null distribution of the average distance from randomly selected points (this varies somewhat across groups as it depends on each group’s data distribution). The asterisks denote statistical significance relative to this null distribution, one-tailed (***P < 0.001). Here and elsewhere, *P* values are Bonferroni-corrected for multiple comparisons. All groups have a probability mass concentrated closer to integer-ratio rhythms than would be expected by chance. **b**, Categories exhibit small but systematic biases away from integer ratios. The grey dots plot the component means of the Gaussian mixture model computed for each of the 39 groups. The large black dots plot the average location over the 39 groups. The red crosses plot integer ratios. The asterisks denote biases that are statistically significantly different from integer ratios across groups (***P < 0.001; **P < 0.01). The results show that 9 of the 22 categories exhibit consistent biases.

**Extended Data Fig. 2:**
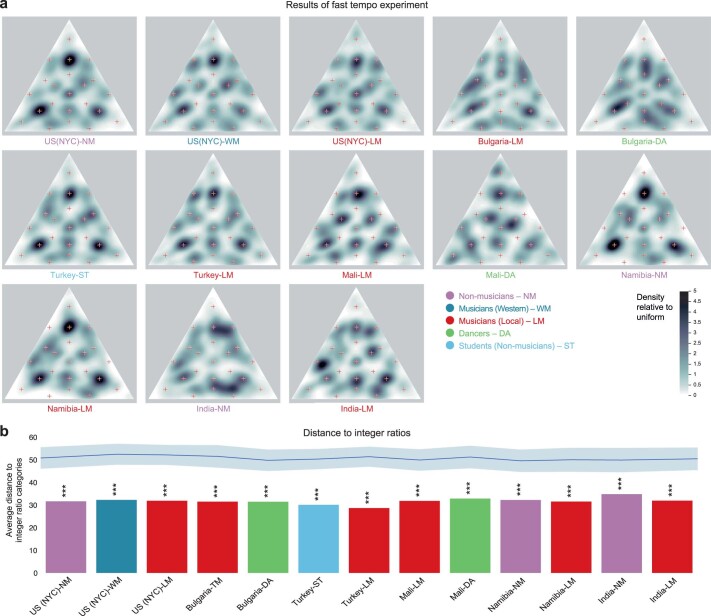
Results of the fast tempo experiment. **a**, Estimated priors for the 13 groups that performed an additional version of the experiment with a faster stimulus tempo (overall pattern duration of 1000 ms compared with 2000 ms in the main experiment). **b**, Average distance from nearest integer ratio rhythm, for each participant group. This measure is small for a prior with all its mass at integer ratio rhythms. Shaded region at top plots mean and 95% confidence intervals of a null distribution of the average distance from randomly selected points. Asterisks mark statistical significance after Bonferroni correction for multiple comparisons (***: p<0.001). All groups have more probability mass concentrated closer to integer ratio rhythms than would be expected by chance.

Visual inspection of the modes suggests that they fall on small-integer-ratio rhythms (the red crosses superimposed on Fig. 3b). To quantify the extent to which this was the case, we computed the average distance between each fifth-iteration reproduction by all participants in a given group and the closest small-integer-ratio rhythm. This measure should be small for priors with modes that are perfectly centred on small-integer ratios. We compared this measure to a null distribution obtained by computing the average minimum distance from a random set of points. All groups produced priors that were closer to small-integer ratios than would be expected by chance (*P* < 0.001 in all cases, via bootstrapping, Bonferroni-corrected; Fig. 4a). Given the diversity of the participant groups, this result suggests that small-integer-ratio rhythm categories are a widespread feature of human mental representations. This conclusion is further supported by two other measures of the overlap of the priors with small-integer ratios (including one where the null distribution was derived from random points constrained to be spaced similarly to the integer ratios; Methods). We obtained similar results in 13 groups who performed the same experiment at a faster tempo (pattern duration of 1,000 ms compared with 2,000 ms in the main experiment; Extended Data Fig. 2 and Supplementary Fig. 1). In all 13 cases, the priors were again closer to small-integer ratios than would be expected by chance (*P* < 0.001 in all groups, via bootstrapping; Extended Data Fig. 2b).

We also found that some of the rhythm categories were systematically biased away from the closest small-integer ratio. Of the 22 analysed small-integer ratios, 9 had corresponding modes (estimated with a Gaussian mixture model) that were slightly but significantly biased away from the integer ratio (Fig. 4b). These biases were present cross-culturally and were similar to those previously observed in US participants. Specifically, the short intervals in the categories 1:2:3 and 2:1:3 (and their cyclic permutations) were consistently lengthened relative to the medium intervals, causing the rhythms to shift slightly towards the centre of the rhythm space (isochrony). This lengthening of the short element is characteristic of rhythm performance studied in European musicians^42^. A similar bias was present for 2:2:3 (and its cyclic permutations). This result suggests that some specific deviations from small-integer ratios are also a widespread feature of mental representations of rhythm.

**Extended Data Fig. 3:**
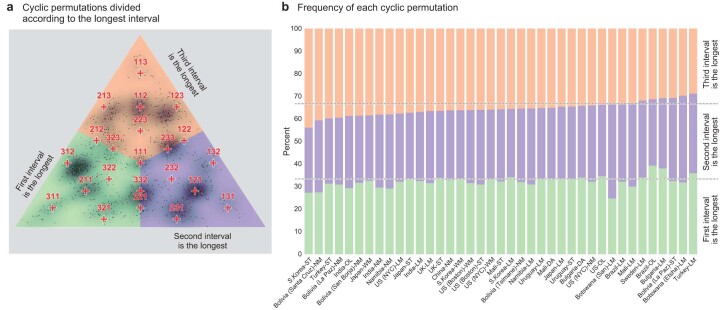
Analysis of symmetry. **a**, A partition of the triangular rhythm space into rhythms where the first, second, or third interval is the longest (green, purple, and red, respectively). The prior shown here for illustration purposes is that from Indian musicians (from the fast tempo experiment). **b**, Relative frequency of tapped responses in the final iteration that fell into each of the sections.

Another feature of the data that is common across cultures is the tendency for the response distributions to be symmetric across cyclic permutations (Fig. 3). For example, the modes at 1:1:2, 1:2:1 and 2:1:1 are about equally prominent for a given participant group. However, there was reason to think that the most frequently occurring permutations would be those where the long interval occurs at the end^43–45^, because if this configuration is played cyclically, the long interval provides a gap that helps the pattern to group according to Gestalt principles^43^. When we compared the frequency of each cyclic permutation in each participant group, we in fact found a consistent trend in this direction: the permutation with the long interval at the end occurred more frequently in 31 of 39 groups (Extended Data Fig. 3). Similarly, in 33 of 39 groups, participants placed their first tap immediately after the long interval, suggesting that most participants in most groups tended to hear this onset as the ‘beginning’ of the pattern. See Extended Data Fig. 3 (and ‘Cyclic permutations and an analysis of symmetry’ in Methods) for further analysis.

### Rhythm priors vary cross-culturally

Despite the consistent presence of discrete modes that tend to overlap small-integer ratios, the measured priors varied across groups. To examine the dominant dimensions of variation, we performed multidimensional scaling^46^ on the measured priors. Two dimensions of variation captured considerable variance (85.9% of the variance in the intergroup distances measured by Jensen–Shannon divergence) and are shown here to facilitate visualization.

**Fig. 5:**
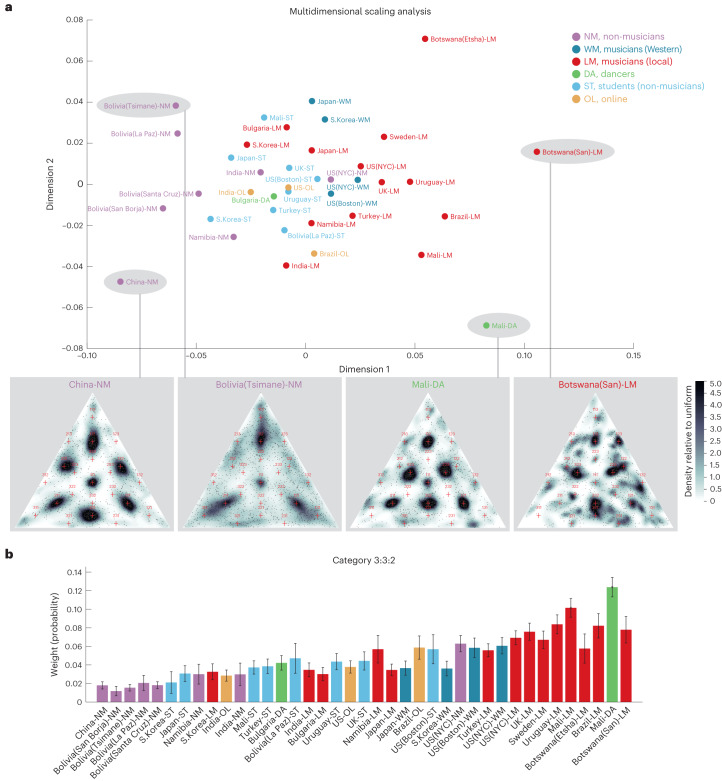
Sources of cross-cultural variation in mental representations of rhythm. **a**, Participant groups positioned in two dimensions via multidimensional scaling of their estimated rhythm priors. Group names are coloured to indicate musicians, dancers, non-musicians and university students. The insets show rhythm priors for the four participant groups lying at the corners of the multidimensional scaling space. These groups represent the extremes of the variation we observed. Note that for all analyses of specific rhythms, we averaged across the cyclic permutations of that rhythm (for example, 1:1:2, 1:2:1 and 2:1:1), as the differences between permutations were typically modest. See Extended Data Fig. 3 for an analysis of differences between cyclic permutations. **b**, Strength of the 3:3:2 rhythm (the average weight of the components at 3:3:2, 2:3:3 and 3:2:3 in the fitted Gaussian mixture model) for all participant groups, ordered according to their positions on the horizontal dimension of the multidimensional scaling space. This rhythm characterizes one dominant dimension of variation across groups. The error bars plot s.e.m., derived from 1,000 bootstrap samples.

**Extended Data Fig. 4:**
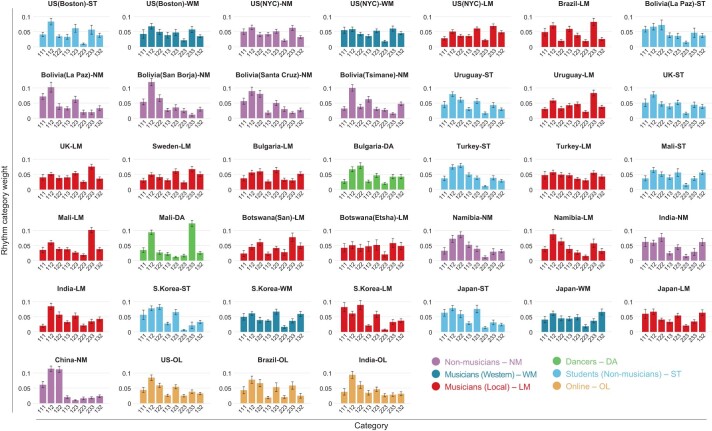
Category weights for all 39 groups. Category weights were obtained from fitting a constrained Gaussian mixture model to the data; error bars reflect standard deviation of the weight estimate derived from 1000 bootstrap samples (see methods).

**Extended Data Fig. 5:**
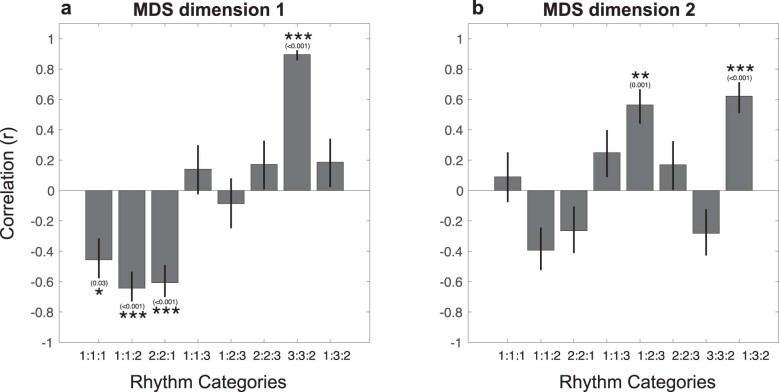
Correlations between multidimensional scaling dimensions and rhythm category weights. **a**, The correlation between the weights given to each of a subset of small-integer-ratio rhythms in a Gaussian mixture model fitted to the prior, and the position along the first (horizontal) multidimensional scaling dimension (from the analysis of Fig. 5a). Dimension 1 is highly correlated with the strength of category 3:3:2. Asterisks denote statistical significance of the correlations, after Bonferroni correction for multiple comparisons (***: p < 0.001; **: p < 0.01; *: p < 0.05). Error bars plot one standard deviation. **b**, Same as **b**. but for the second (vertical) multidimensional scaling dimension, which is significantly correlated with categories 1:2:3 and 1:3:2.

**Extended Data Fig. 6:**
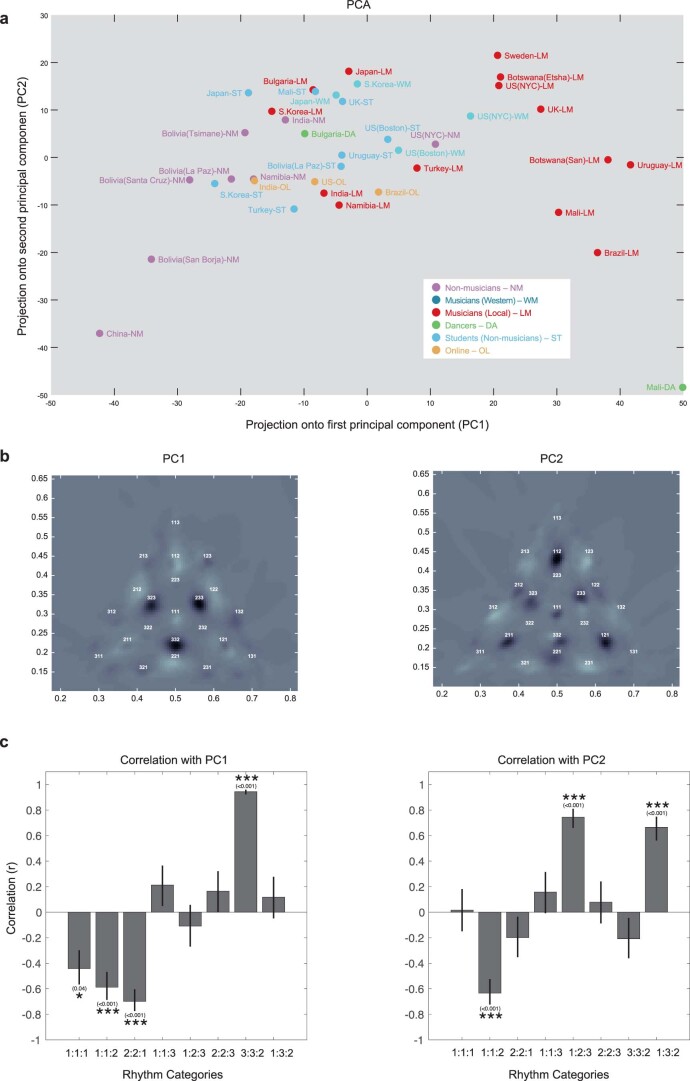
Principal component analysis alternative to the Multidimensional Scaling analysis of Fig. 4. **a**, Projection of each group’s prior onto the first two principal components of the measured priors. **b**, The first two principal components capture variation in the strength of particular integer ratio categories. **c**, Correlation of the PCA projections and GMM category weights (analogous to Extended Data Fig. 5 but for PCA). Asterisks mark statistical significance after Bonferroni correction for multiple comparisons (***: p < 0.001; **: p < 0.01; *: p < 0.05). Error bars plot one standard deviation.

Figure 5a shows the measured priors for each group arranged according to their positions in this space. The four groups lying at the ‘corners’ of the multidimensional scaling space provide a snapshot of the variation across groups, along with features that consistently appear (Fig. 5a, insets). All groups show modes at isochrony (1:1:1) and 1:1:2. However, the presence of other small-integer rhythms varies. We quantified the prominence of different integer-ratio rhythms as the weights on individual components of a Gaussian mixture model fitted to the data (with each component constrained to be unambiguously associated with a different integer ratio; see Extended Data Fig. 4 for the weights of components for all groups). The presence or absence of a mode at the 3:3:2 rhythm is a major source of variation, accounting for the horizontal dimension of the multidimensional scaling space (position along this dimension was highly correlated with the weight on the 3:3:2 category; *r*_37_ = 0.90; *P* < 0.001; 95% confidence interval (CI), (0.81, 0.94); Fig. 5b). The first dimension was also correlated with a reduction in the weight of the ‘simpler’ categories, including isochrony (1:1:1: *r*_37_ = −0.46; *P* = 0.028; 95% CI, (−0.67, −0.16); 1:1:2: *r*_37_ = −0.64; *P* < 0.001; 95% CI, (−0.8, −0.41); 2:2:1: *r*_37_ = −0.61; *P* < 0.001; 95% CI, (−0.77, −0.36)). The second multidimensional scaling dimension is less obviously interpretable but also correlated significantly with two of the small-integer-ratio rhythms (1:2:3: *r*_37_ = 0.56; *P* = 0.001; 95% CI, (0.3, 0.75); 1:3:2: *r*_37_ = 0.62; *P* < 0.0001; 95% CI, (0.38, 0.78); Extended Data Fig. 5), and it thus can be seen as partially embodying the strength of 6/8 rhythms. The groups at the corners also exhibit variation in the presence of 2:2:1, such that all possible combinations of modes at 2:2:1 and 3:3:2 occur: the Tsimane’ non-musicians from Bolivia show neither of these two modes, the San musicians from Botswana show both, the dancers from Mali show 3:3:2 but not 2:2:1 and the non-musicians from China show 2:2:1 but not 3:3:2. We also analysed the data using principal component analysis; the first two components captured variation along the same modes highlighted by the multidimensional scaling analysis (Extended Data Fig. 6).

The US and UK participant groups were all positioned close to the centre of the multidimensional scaling space. One speculative possibility is that this position reflects the widespread influence of Western music around the world, with different cultures having incorporated its influence in different ways.

### Cross-cultural variation is less evident in university students

**Fig. 6:**
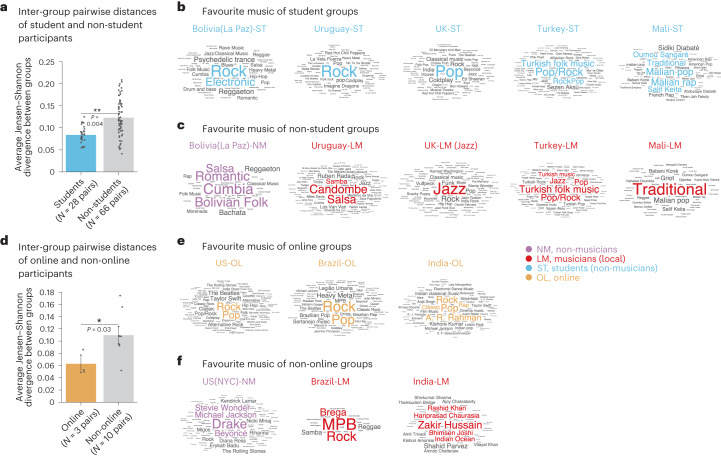
Cross-cultural variation is reduced in university students and online participants. **a**, Jensen–Shannon divergence between the estimated priors for pairs of student groups in different countries and pairs of non-student groups in the same set of countries (one for each student group). The error bars plot s.e.m., computed via bootstrapping. The dots indicate individual data points (to aid visualization, we added a small random offset to the horizontal position of each point). The asterisks mark one-sided statistical significance (**P < 0.01; *P < 0.05) computed via 10,000 bootstrap samples. **b**, Word clouds of favourite musical genres listed by student groups. **c**, Word clouds of favourite musical genres listed by non-student groups. **d**, Same as **a**, but for online and non-online groups. **e**,**f**, Same as **b**,**c**, but for online and non-online groups.

The positioning of the groups in the multidimensional scaling space (Fig. 5a) also suggests that the priors in university students and online participants from countries around the world are relatively similar to each other and to the priors of US participants. To quantify this effect in students, we first measured the distance (Jensen–Shannon divergence) between the estimated priors for pairs of student groups in different countries and compared it to that for pairs of non-student groups (from the same countries from which the student groups were drawn). This comparison revealed that the distance between student groups was significantly smaller than that between non-student groups (*P* = 0.004, permutation test; Cohen’s *d* = 1.06; difference of mean distances, 0.039; 95% CI, (0.03, 0.05); Fig. 6a). This result indicates that student participants underrepresent cross-cultural diversity. This conclusion is supported by the self-reported music listening habits of the participants, which are notably more similar between the student groups than between the corresponding non-student groups from the same countries (Fig. 6b,c and Supplementary Table 2).

### Cross-cultural variation is less evident in online participants

We conducted an analogous analysis in online participants and obtained a similar result (smaller intergroup distances than for non-online groups; *P* = 0.03, permutation test; Cohen’s *d* = 1.46; difference of mean distances, 0.047; 95% CI, (0.02, 0.07); Fig. 6d). This result indicates that online participants also underrepresent cross-cultural diversity. As with the student groups, this conclusion is supported by self-reported music listening habits, which are more similar between the online groups than between non-online groups from the same countries (Fig. 6e,f and Supplementary Table 2).

### Students and online participants resemble US participants

To quantify the similarity of student and online groups to US participants, we measured the average distance (again using Jensen–Shannon divergence as the distance measure) between priors of either student or online groups to the prior of the US student group (taken as a representative group of US participants). We then compared these two average distances to those between the US group and random sets of groups of the same size (who were not students or online participants). Student and online groups were significantly closer to US participants than were the other groups (students: *P* < 0.001; Cohen’s *d* = 2.7; difference of mean distances, 0.027; 95% CI, (0.009, 0.05); online: *P* = 0.014; Cohen’s *d* = 1.86; difference of mean distances, 0.032; 95% CI, (0.002, 0.07); via bootstrapped Jensen–Shannon divergence, as described in ‘Analysis of student and online groups’ in Methods).

While the online participants spanned a wide range of ages, student participants tended to be younger than other groups (Extended Data Table 1). To control for possible effects of age, we repeated both analyses, restricting participants to the age range of 20–40 (Methods). The pairwise distance between student and online groups was still significantly smaller than that between non-student and non-online groups (students: *P* < 0.001; Cohen’s *d* = 1.66; difference in means, 0.069; 95% CI, (0.05, 0.08); online: *P* < 0.001; Cohen’s *d* = 2.78; difference in means, 0.081; 95% CI, (0.05, 0.1); via bootstrapping). So was the distance between the US student group and other student and online groups when compared with that between the US student group and non-student/non-online groups (students: *P* < 0.001; Cohen’s *d* = 3.33; mean difference, 0.058; 95% CI, (0.03, 0.09); online: *P* < 0.001; Cohen’s *d* = 1.88; mean difference, 0.055; 95% CI, (0.02, 0.1); via bootstrapping). In addition, we compared priors of younger (under 35) and older (over 35) online participants and found no significant differences in each of the three tested locations (via bootstrapped Jensen–Shannon divergence; US online group: *P* = 0.09; Cohen’s *d* = 0.66; mean difference in distance, 0.02; 95% CI, (−0.03, 0.06); India online group: *P* = 0.11; Cohen’s *d* = 1.4; mean difference in distance, −0.007; 95% CI, (−0.07, 0.05); Brazil online group: *P* = 0.4; Cohen’s *d* = 0.32; mean difference in distance, −0.002; 95% CI, (−0.09, 0.1)).

These findings suggest that certain lifestyle factors associated with student and online participants (plausibly including socio-economic status or access to global media and internet) involve exposure to globalized culture that is sufficient to produce mental representations of rhythm that are similar to those of US residents. As a consequence, student and online participants (the populations typically studied in psychology, neuroscience and music cognition^37,41,47,48^) underrepresent global variability in music perception^37^.

### Culture-specific variation can be linked to the music in a group

**Fig. 7:**
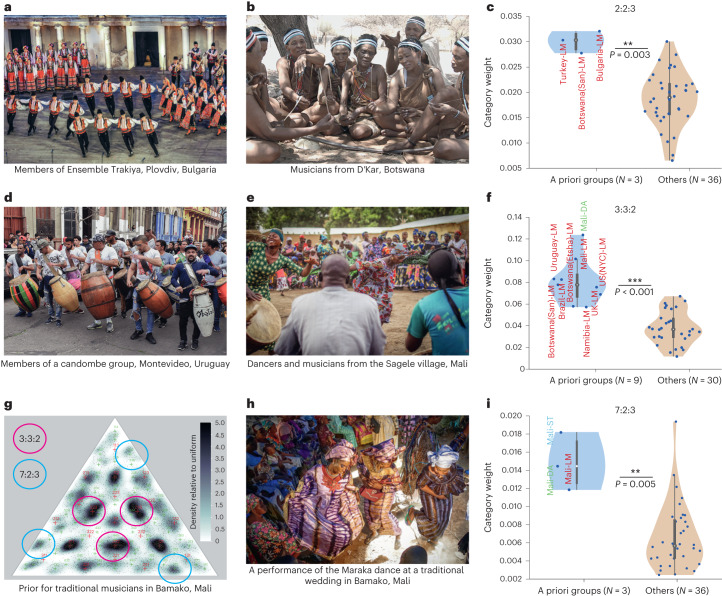
Rhythm priors reflect established culture-specific musical features. **a**, Dancers and musicians from Ensemble Trakiya in Plovdiv, Bulgaria. Here and in other panels, verbal informed consent was obtained from the groups in each photo. Credit: Ivan Banchev. **b**, Musicians from D’Kar, Botswana. Credit: Van K. Yang. **c**, Violin plots showing the strength of the 2:2:3 rhythm for all tested groups, separated into those in whose music the rhythm is prominent, and all other groups. Here and in other violin plots in this paper, the open circle plots the median, and the top and bottom of the grey bar plot the 75th and 25th percentiles. Whiskers (thin lines) are computed using Tukey’s method and reflect the range of non-outlier points (see 'Violin plots' for details). The violin plots are kernel density estimates of the data distribution. Here and in **f**,**i**, the asterisks mark statistical significance via one-sided Wilcoxon rank-sum tests (***P < 0.001; **P < 0.01; *P < 0.05). The 2:2:3 rhythm is strongly represented in the priors of traditional musicians in Bulgaria, Turkey and Botswana, when compared with all other groups. **d**, Members of a candombe group in Montevideo, Uruguay. **e**, Dancers and musicians from the Sagele village in Mali. **f**, The strength of the 3:3:2 rhythm for all tested groups, separated into those in whose music the rhythm is prominent (that is, the music of African and Afro-diasporic traditions), and all other groups. **g**, Rhythm prior for drummers from Bamako, Mali, showing modes at 3:3:2 and 7:2:3. **h**, A performance of the Maraka dance (featuring the 7:2:3 rhythm) at a traditional wedding in Bamako, Mali. **i**, Strength of the 7:2:3 rhythm for all tested groups.

What underlies the observed variation across groups? In principle, variation could be due to any of the many factors that varied across groups, including non-musical factors such as the rhythms prominent in spoken language or in environmental sounds. But in several cases, variation in rhythm priors had obvious links to rhythms prominent in local musical systems. For instance, the mode at the 2:2:3 rhythm was pronounced in groups that have this rhythm in their local musical tradition^49–51^: traditional musicians in Turkey, Botswana and Bulgaria (Fig. 7a,b). As shown in Fig. 7c, the weights assigned to the 2:2:3 rhythm in a Gaussian mixture model fit to the prior of each group were substantially higher in these groups than in most other groups (*P* = 0.003, via a one-sided Wilcoxon rank-sum test; Cohen’s *d* = 2.17; mean difference in weight, 0.011; 95% CI, (0.003, 0.02)). This result is consistent with previous developmental work showing differences in sensitivity to the 2:2:3 rhythm in US and Turkish adults^52^, but not infants^53^, suggesting that sensitivity to the rhythm may be lost without exposure.

The 3:3:2 rhythm can also be related to specific musical systems—in this case, those of African and Afro-diasporic music. In particular, it features prominently in many sub-Saharan musical styles^54,55^, is popular in Afro-Cuban and Latin music (where it is referred to as ‘tresillo’), and is characteristic of many Afro-diasporic traditions from Cuba, Brazil, Uruguay and North America^56–59^, among others (Fig. 7d,e). In the priors estimated from the experiment, the 3:3:2 mode was strongest in dancers from the Sagele village in Mali (Figs. 5b and 7e). We recorded a representative corpus of their musical repertoire and found that 46% of the excerpts recorded in the corpus featured a prominent 3:3:2 pattern (see Methods for additional details). We similarly found strong 3:3:2 weights in all other groups of musicians and dancers from African and Afro-diaspora traditions (musicians from Botswana, Mali, Uruguay and Brazil, and both US and UK jazz musicians; Fig. 7f; significantly higher weights for this mode when compared with the other participant groups, *P* < 0.001, via a one-sided Wilcoxon rank-sum test; Cohen’s *d* = 2.60; mean difference in weight, 0.043; 95% CI, (0.035, 0.051)).

### Perceptual modes can occur at moderately complex integer-ratio rhythms

Another example of an idiosyncratic feature that can be linked to a musical system is the mode at 7:2:3 evident in drummers in Mali (Fig. 7g). This rhythm is noteworthy because it is defined by a relatively complex integer ratio. We took several steps to establish that this rhythm is prominent in the local musical tradition (see ‘Analysis of specific modes’ in Methods). First, we observed that some participants recognized the rhythm during the experiment session. They identified it as ‘Maraka’, which is a popular local piece of dance music that uses a 7:2:3 rhythmic pattern (Fig. 7h). To validate this recognition of the rhythm in the musician groups we tested, we conducted post-experiment interviews after the end of the main experimental session with the help of an ethnographer team member (R.P.). We estimated the empirical modes of the distribution, synthesized stimuli corresponding to the mode near 7:2:3 and asked participants to identify it. The Malian drummer participants repeatedly identified the pattern as ‘Maraka’.

As shown in Fig. 7i, the 7:2:3 mode was stronger in the three groups from Mali than in all other groups (*P* = 0.005, via a one-sided Wilcoxon rank-sum test; d.f. = 37; Cohen’s *d* = 2.33; mean difference in weight, 0.0082; 95% CI, (0.007, 0.01)). This result suggests that even relatively complex rhythms can form perceptual categories but that they are strongly dependent on musical experience within cultural environments where the corresponding rhythm prevails.

### Culture-specific rhythm priors predict categorical perception

**Extended Data Fig. 7:**
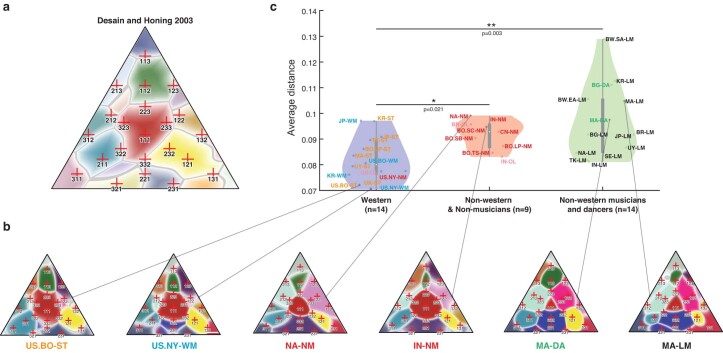
Rhythm categorization. We simulated categorization judgments with a Bayesian model using the estimated priors from each group, and compared the results to categorization judgments of rhythms at different points within the rhythm triangle by Western musicians, previously obtained by Desain and Honing^9^. In the original experiment, the musician participants heard a rhythm and chose one of 17 category choices, each defined by musical notation. **a**, The results of the experiment are summarized as a set of regions that are associated with each musically notated rhythm (as the most frequent category choice). Human results are replotted from the data of Desain and Honing. **b**, Analogous regions for the model categorization judgments using each group’s prior. **c**, Average distance between the categories predicted by a group’s prior and those measured in Western musicians. For every sampled point on the rhythm triangle, we compared the model prediction with the category most frequently selected by human participants in the Desain and Honing experiment, and measured the distance measured in the three-dimensional space of the three intervals in the rhythm, expressed as ratios (proportions of the total pattern duration). We then averaged this distance for each rhythm in the experiment (that is, every sampled point on the rhythm triangle) to yield an overall measure of the match between the model’s predictions and Western musician category judgments. We pooled participants into three sets of groups, defined as follows: Western participants (US.BO-ST, US.BO-WM, US.NY-NM, US.NY-WM, BO.LP-ST,UY-ST,UK-ST, TK-ST, MA-ST, KR-ST, KR-WM, JP-ST, JP-WM, US-OL), non-Western non-musicians (BO.LP-NM,BO.SB-NM,BO.SC-NM,BO.TS-NM, NA-NM, IN-NM, CN-NM, BR-OL, IN-OL) and non-Western musician and dancers (BR-LM,UY-LM, SE-LM, BG-LM, BG-DA, TK-LM, MA-LM, MA-DA, BW.SA-LM, BW.EA-LM, NA-LM, IN-LM, KR-LM, JP-LM). We excluded jazz musicians in the US and UK as there was not a natural hypothesis regarding how their priors would predict the categories of Western classical musicians. Asterisks denote significance of a one-sided Wilcoxon rank-sum test (**: p<0.01; *: p<0.05).

To test whether the rhythm ‘categories’ (modes) in the measured priors could predict perceptual categorization, we simulated categorization judgements with a Bayesian model based on a Gaussian mixture model fit to the estimated priors from each group (in which each mixture component corresponds to a rhythm category). We then compared the results to previously published categorization judgements by Western musicians of rhythms at different points within the rhythm triangle^9^. Because the category choices were defined by musical notation, this experiment was possible only in Western musicians. The results of the experiment can be expressed as a set of regions that are associated with each musically notated rhythm (Extended Data Fig. 7a). The categories predicted by priors measured in US participant groups generally provided good matches to the experimentally obtained category boundaries of Western musicians (Extended Data Fig. 7b, left). By contrast, the category boundaries for the non-Western groups we tested provided worse matches (Extended Data Fig. 7b, right). We quantitatively assessed the match between the categories predicted by a group’s prior and those measured in Western musicians via the average of the distances over all points in the triangle between the categories assigned by the model and the majority category in the Desain and Honing experiment (see ‘Category predictions from rhythm priors’ in Methods). As shown in Extended Data Fig. 7c, when evaluated in this way, both the non-Western non-musician groups and the non-Western musician and dancer groups that we tested produced significantly worse matches than the Western groups (here defined as student, Western musician and US groups) via Wilcoxon rank-sum tests (non-Western non-musicians: *P* = 0.021; Cohen’s *d* = 1.2; difference of mean distances, 0.01; 95% CI, (0.0035, 0.015); non-Western musician and dancer groups: *P* = 0.003; Cohen’s *d* = 1.2; difference of mean distances, 0.015; 95% CI, (0.0062, 0.023)). This result is consistent with the idea that the prior measured by our iterated tapping experiment reflects a perceptual representation and so can predict purely perceptual judgements. The result also shows that the cross-cultural variation in the measured priors has the expected consequences for perceptual category predictions (in this case, worse predictions of Western musicians’ categories for groups whose musical experience presumptively deviates substantially from that of Western musicians).

### Musicianship does not obviously alter rhythm priors

**Extended Data Fig. 8:**
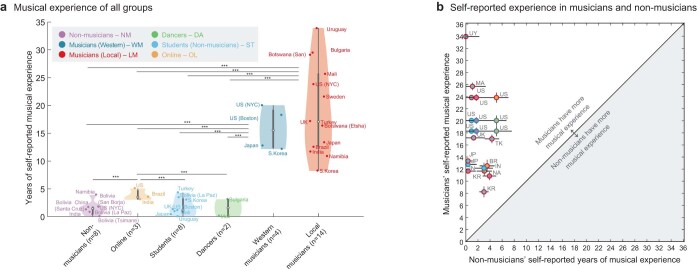
Musical experience in musicians and non-musicians. **a**, Average self-reported years of musical experience for each participant group. Both traditional and Western musicians self-reported significantly more years of experience than other participant groups. In the violin plots, the open circle plots the median, and the top and bottom of the gray bar plot the 75^th^ and 25^th^ percentiles. We plotted significant comparisons with a threshold of p<0.001 (one-sided Wilcoxon test, corrected for multiple comparisons with Bonferroni correction). **b**, Scatter plot of self-reported years of musical experience for musician and non-musician groups from the same country. Each point represents a pair of non-musicians (x-axis) and musicians (y-axis) groups.

We previously found that rhythm priors were similar for musicians and non-musicians in Boston, USA^26^, suggesting that priors are driven by ‘passive’ exposure to music rather than explicit training or practice. We replicated this finding here in multiple other groups of musicians who play Western music and corresponding groups of non-musician students with exposure to Western music (in South Korea, Japan and the United States). In each case there was no significant difference between priors derived from splits of the data within and across groups, as evaluated by bootstrapping the Jensen–Shannon divergence using the procedure described in the Methods section ‘Significant distance between two groups’ (South Korea: *P* = 0.17; Cohen’s *d* = 1.1; mean difference between across- and within-group distance, 0.03; 95% CI, (−0.01, 0.07); Japan: *P* = 0.051; Cohen’s *d* = 1.1; mean difference between across- and within-group distance, 0.03; 95% CI, (−0.02, 0.08); United States: *P* = 0.56; Cohen’s *d* = 0.64; mean difference between across- and within-group distance, 0.02; 95% CI, (−0.03, 0.07)). For the sake of brevity, here and elsewhere we classify and refer to participants as ‘musicians’ or ‘non-musicians’ (Methods), cognizant that the Western concept of musicianship may not fully apply to other societies and that there can be a continuum of levels of participation in musical activities. We nonetheless found that years of self-reported musical experience differed between groups classified as ‘musicians’ or ‘non-musicians’ (Extended Data Fig. 8a); pairwise comparisons between musician and non-musician groups in the same country were statistically significant in all cases (Extended Data Fig. 8b; *P* < 0.001 in all cases via one-sided Wilcoxon tests; Cohen’s *d* ranged between 1.65 and 4.27; the difference between median years of experience ranged from 6 to 37 years).

We did find multiple cases where musicians and non-musicians in the same country exhibited distinct priors, but these all seem explainable by differences in musical exposure. Specifically, local musician groups in Mali and Uruguay differed from other groups in those countries (again assessed using bootstrapped Jensen–Shannon divergence, comparing across- and within-group distance; MA-LM versus MA-DA: *P* = 0.002; Cohen’s *d* = 1.8; mean difference in distance, 0.04; 95% CI, (0.0006, 0.07); MA-LM versus MA-ST: *P* = 0.018; Cohen’s *d* = 2.6; mean difference in distance, 0.07; 95% CI, (0.02, 0.1); UY-LM versus UY-ST: *P* = 0.036; Cohen’s *d* = 2.3; mean difference in distance, 0.06; 95% CI, (0.01, 0.1)), presumably because they have internalized different types of music in the cultural milieu of the tradition they specialize in (Fig. 6b,c,e,f). We thus have no reason to suppose that musicianship per se qualitatively alters mental representations of rhythm. The signature properties of discrete modes at small-integer ratios are clearly evident in both musicians and non-musicians.

### Musicianship improves tapping precision

**Fig. 8:**
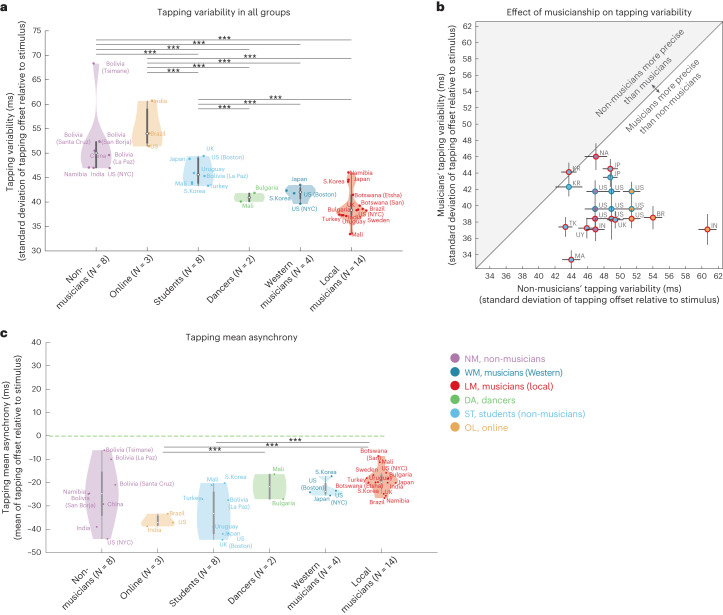
Tapping variability and musical experience. **a**, Tapping variability (standard deviation of the asynchrony) as a function of musical experience. The open circle plots the median, and the top and bottom of the grey bar plot the 75th and 25th percentiles. Whiskers (thin lines) are computed using Tukey’s method and reflect the range of non-outlier points (see 'Violin plots' for details). We plotted significant comparisons with a threshold of *P* < 0.001 (one-sided Wilcoxon test, corrected for multiple comparisons with Bonferroni correction). We note that there are only two groups categorized as dancers and that for one of the groups (that in Mali), there was a demographic confound compared with the musician group in the same country (dancers were predominantly female, while musicians were predominantly male). The apparent differences with other types of groups should thus be considered provisional given the small number of groups. **b**, Scatter plot of tapping variability for musician and non-musician groups from the same country. The error bars plot s.e.m., computed via bootstrapping. **c**, Mean asynchrony of tapped responses as a function of musical experience. Same conventions as **a**.

Strong effects of musicianship were, by contrast, evident in the variability of tapped reproductions, standardly used as a measure of tapping precision^3^ (Fig. 8a). We compared tapping variability (quantified as the standard deviation of the asynchrony between stimulus and response^3^) between 21 pairs of musician and non-musician groups in the same country. Tapping variability was lower in musicians than in non-musicians (Fig. 8b) in 20 of the 21 pairs (*P* < 0.001 via a binomial test). This difference was statistically significant (*P* < 0.05) in 15 of the 21 individual pairs via one-sided Wilcoxon tests. For these 15 pairs, Cohen’s *d* ranged between 1.08 and 2.36; the difference in median variability ranged between 5.41 ms and 23.69 ms. These results are consistent with a large literature in Western musicians showing perceptual and production advantages in musical tasks^60–63^, including tapping to a beat^3^, but they provide evidence that these musicianship advantages are present cross-culturally. The results support a distinction between musicians and non-musicians that extends beyond North America and Western Europe^27,64^. Despite these musicianship effects, we saw that every participant group exhibited a tendency to tap slightly before the beat, quantified as a negative mean stimulus–response asynchrony (Fig. 8c)^3,65^. This negative mean asynchrony has long been noted as a feature of tapping in Western participants^3^; our responses suggest that it is present across cultures.

### No evidence that language influences rhythm priors

It is natural to wonder whether the prior on rhythm might be influenced by experiential factors outside of music, most obviously spoken language. Speech has been argued to influence rhythm perception^66–68^, but most recent evidence indicates segregation of speech and music analysis in the brain^69^. Several findings from our study suggest that language does not strongly influence rhythm representations.

**Extended Data Fig. 9:**
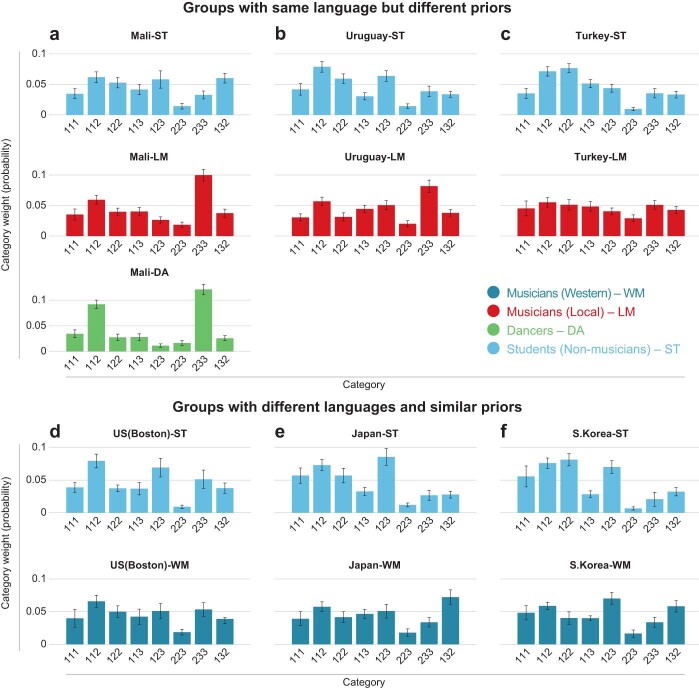
Comparison of rhythm priors in groups speaking different languages or with differing degrees of musical expertise. **a–c**, Groups speaking the same language that exhibit different priors. Graphs plot weights of different rhythm categories in a Gaussian mixture model fit to the data of each group (see section on Gaussian mixture model fits in the Methods). Error bars plot standard deviation of the estimates derived from 1000 bootstrap samples (see methods). **d–f**, Groups speaking different languages that exhibit similar priors. The comparison of these groups also provides an example where priors can be similar between musicians and non-musicians if the music they consume is plausibly similar (compare the student non-musicians to the Western musicians). Same plotting conventions as in **a-c**.

First, we observed clear examples of groups who speak the same language but whose rhythm priors were quite different. The clearest example is the three groups in Mali (local musicians, dancers and students). All three groups speak a nearly identical set of languages but have obviously distinct rhythm priors (via bootstrapped Jensen–Shannon divergence; MA-LM and MA-ST: *P* = 0.012; Cohen’s *d* = 2.6; mean difference between across- and within-group distance, 0.07; 95% CI, (0.02, 0.1); MA-LM and MA-DA: *P* = 0.009; Cohen’s *d* = 1.8; mean difference in distance, 0.04; 95% CI, (0.0006, 0.07); MA-ST and MA-DA: *P* < 0.001; Cohen’s *d* = 4.5; mean difference in distance, 0.1; 95% CI, (0.08, 0.2)). Specifically, both the local musicians and dancers in Mali had significantly higher weights on the 3:3:2 rhythms than did Malian students (via bootstrapped Gaussian mixture model fits as described in the Methods section ‘Category weight for the 3:3:2 rhythm’; MA-ST and MA-LM: *P* = 0.003; Cohen’s *d* = 5.3; mean weight difference, 0.064; 95% CI, (0.039, 0.087); MA-ST and MA-DA: *P* < 0.001; Cohen’s *d* = 6.7; mean weight difference, 0.087; 95% CI, (0.061, 0.11); see Extended Data Fig. 9a for the category weights). We found a similar result for comparisons of students and local musicians in Uruguay (distinct rhythm priors as evaluated by bootstrapped Jensen–Shannon divergence; *P* = 0.013; Cohen’s *d* = 2.2; mean difference in distance, 0.06; 95% CI, (0.01, 0.1); the weight on the 3:3:2 rhythm again differed between groups, *P* < 0.001; Cohen’s *d* = 3.1; mean weight difference, 0.04; 95% CI, (0.014, 0.065); see Extended Data Fig. 9b for the category weights). An analogous result was evident in Turkey, where local musicians had significantly higher weights on the 2:2:3 rhythm, which is associated with Balkan and Turkish music, compared with Turkish students (Extended Data Fig. 9c; *P* = 0.001; Cohen’s *d* = 3; mean weight difference, 0.02; 95% CI, (0.0071, 0.033); the priors themselves were also again significantly different as evaluated by bootstrapped Jensen–Shannon divergence; *P* = 0.02; Cohen’s *d* = 1.3; mean difference in distance, 0.02; 95% CI, (−0.008, 0.05)).

Second, we observed several examples of similar rhythm priors despite distinct spoken language experience. Both university students and Western-trained musicians in Japan and South Korea exhibited priors that were not obviously different from those of US participants (Extended Data Fig. 9d–f; no significant difference in either case as evaluated by bootstrapped Jensen–Shannon divergence: Japanese students and US students: *P* = 0.77; Cohen’s *d* = 0.47; mean difference in distance, 0.01; 95% CI, (−0.03, 0.05); Japanese Western musicians and US Western musicians: *P* = 0.11; Cohen’s *d* = 1.1; mean difference in distance, 0.02; 95% CI, (−0.02, 0.06); South Korean students and US students: *P* = 0.25; Cohen’s *d* = 1.3; mean difference in distance, 0.04; 95% CI, (−0.01, 0.1); South Korean Western musicians and US musicians: *P* = 0.44; Cohen’s *d* = 0.92; mean difference in distance, 0.02; 95% CI, (−0.02, 0.07)). Although the student groups in these countries undoubtedly had some exposure to English, it was not their native language. We cannot exclude the possibility that language could in some cases have some influence on rhythm representations, but our study provides no evidence for such influences.

## Discussion

We conducted a large-scale cross-cultural study of music perception. Our aim was to assess whether discrete mental representations of musical rhythm are present across cultures and whether the associated mental categories are fixed or dependent on some aspect of life experience. We ran the same iterated reproduction experiment on 39 different groups around the world. We found that all groups imposed discrete structure on the continuous space of simple rhythms. Mental ‘categories’ (modes) tended to be small-integer-ratio rhythms, but the categories varied across groups and could often be linked to specific rhythms prominent in the local musical culture. Our study provides unambiguous evidence for properties of music cognition that are present across cultures, but it also demonstrates substantial cultural variation linked to culture-specific musical experience.

### Universality and cultural specificity

Given that music is present in all known societies but also varies considerably across cultures^14–16,70^, it must result from interactions between biological constraints and culture-specific experience. What do our results reveal about these constraints and experiential influences? The most salient feature of our results is the cross-cultural presence of relatively discrete modes within the mental prior over rhythms. We refer to these modes as ‘categories’ because they yield characteristics of categorical perception, biasing the perception of nearby rhythms towards the mode centre^26,71^ and predicting the boundaries between perceptual rhythm categories (Extended Data Fig. 7). These discrete categories were present in every group we tested. Discrete mental representations probably help stabilize musical systems. If a reproduction of a heard piece of music is attracted to discrete categories in the listener’s mind^72,73^, inaccuracies in musical reproduction are less likely to be perceived and thus less likely to be transmitted as a song is passed along between individuals^6,74,75^.

The prevalence of discrete categories across such diverse groups suggests that the conception of music as combinations of discrete elements is not merely an invention of the Western academic and music traditions but is instead fundamental to the human experience of music around the world. The quantization of a continuous space into discrete categories allows signals to be represented and stored more efficiently^76,77^. This efficiency gain probably makes patterns composed of the rhythm categories easier to learn and share, which may further aid cultural transmission.

The particular categories we observed were also non-arbitrary. Categories were present at small-integer-ratio rhythms in every group tested and thus appear to be widespread features of human mental representations^15^. This result could in principle reflect innate biases favouring integer-ratio rhythms. However, it is also clear that any such biases at best only partially constrain adult perceptual systems, because the specific integer ratios that were present as categories varied substantially across groups. We also cannot exclude the possibility that mental representations simply mirror the musical systems to which listeners are exposed (akin to how phonetic categories are thought to be consequences of linguistic exposure), which might feature integer ratios for other reasons (for example, production constraints^78^ from the periodic nature of motor behaviours).

We found evidence that much of the variation in categories is linked to the local musical systems the participants had grown up with^27,79^ (Fig. 7). Moreover, the relevant experience appears to primarily be consumption rather than production of music—musicianship appears to influence priors primarily insofar as it alters musical experience. For instance, musicians who play classical Western music and Western non-musicians exhibited similar priors, presumably because both groups are exposed to similar distributions of music, whereas traditional musicians in some other countries were substantially different from students in cities (for example, Mali, Uruguay and Turkey). Rhythm categories thus appear to be somewhat flexible and culturally dependent (similar to other domains of perception in which categorical perception has been documented cross-culturally, such as colour^80^, speech^81^ and smell^82^). One difference relative to other examples of cross-cultural categorical perception is that the rhythm categories are typically non-verbal, consistent with the idea that discrete representations do not necessarily depend on linguistic labels.

How might other aspects of culture influence rhythm priors? We found evidence that rhythm priors could be dissociated from language, in that we observed several sets of groups that spoke the same language but exhibited distinct priors. The question of whether language influences rhythm priors at all remains open; our groups were not selected to definitively address this issue, in that language covaried along with music across most groups. It remains possible that groups specifically selected to dissociate language from music might show such an influence.

### Small-integer ratios

Small-integer ratios are often proposed to be perceptually favoured in various ways as a consequence of their mathematical simplicity^58,83^. We found that the perceptual categories that were common across all groups were indeed the simplest possible in mathematical terms (1:1:1 and 1:1:2). We also found systematic biases, present cross-culturally, in which more complicated rhythms were shifted to be closer to isochrony (Fig. 4b). However, we also found instances of categories at relatively complex ratios (for example, 7:2:3). We note that although 7:2:3 is a complex rhythm from the perspective of ratios, it can be generated from a fixed train of 12 pulses grouped hierarchically into groups of 2 and 3 (7:2:3 = [2 + 2 + 3] + 2 + 3). The associated five-interval pattern of 2:2:3:2:3 is also common in West African music^54,55^. This observation raises the possibility that small-integer ratios tend to arise due to pressures favouring rhythms that can be generated from isochrony (that is, by dividing an isochronous pulse sequence into groups of small numbers of pulses), rather than simplicity per se. Moreover, the prevalence of the very simplest rhythms (1:1:1 and 1:1:2) across all groups studied could reflect the fact that they can be generated from isochrony in many different ways, and are thus consistent with many different metres. For instance, 1:1:2 can be formed in groups of 4 (1:1:2), 8 (2:2:4), 12 (3:3:6) and 16 (4:4:8) pulses.

### Lack of diversity evident in university students and online participants

Our results illustrate that perceptual phenomena seen in residents of Western Europe and North America (here, the specific prior found in Westerners) may not always generalize to other populations, consistent with the growing awareness of the underrepresentation of human cultural diversity in psychology and cognitive neuroscience^37,41^. Our results also suggest that cross-cultural studies involving convenient participant samples from university communities or online cohorts are similarly likely to underrepresent cultural diversity. University students and online participants in the non-Western countries in our study were substantially more similar to US participants than corresponding non-student groups recruited in person, underscoring the problematic reliance on student and online participants in cognitive science. A wide range of participant groups extending from small-scale societies to villagers to small-town dwellers to urbanites, as well as multiple groups per society with different backgrounds, was critical to providing a strong test of universality and to revealing the extent of diversity in human perception^41,84,85^.

### Limitations

Due to the practical constraints of our experimental paradigm, our experiments were limited to periodic three-interval rhythms, two seconds in duration (though see Extended Data Fig. 2 and Supplementary Fig. 1 for results at a faster tempo in a subset of groups), presented cyclically. We think it plausible that such simple rhythms can serve as building blocks for more complicated rhythms^86^, but they are nonetheless small in scale relative to many natural musical rhythmic motifs, and they do not capture some phenomena that are evident in such longer patterns (for example, metre). It is likely that additional principles govern more extended musical pieces, such as memory constraints, that could be revealed with experiments using longer patterns^74^. We note also that the cyclic stimulus presentation limits our ability to examine phenomena involving the beginning of a rhythm, such as anacrusis (though see Extended Data Fig. 3).

Our paradigm is also constrained by the choice of stimuli. The repeating rhythms we used are limited by the absence of melody. The method could be naturally extended to incorporate melody—for instance, by having participants sing back a note sequence defined by both time and pitch intervals^28^. Such experiments could address whether priors for pitch and time are independent and whether rhythm and melody processing are interdependent^87^. More generally, it seems plausible that humans learn multiple distinct priors for the temporal patterns in different types of sound and that different contexts could invoke distinct priors. For instance, we found in previous work that using spoken phrases as stimuli in an iterated reproduction experiment yielded a completely different prior compared with click patterns^26^. Stimuli based on environmental sounds (for example, based on footstep sounds) could similarly evoke a different prior. There could also be multiple priors on musical rhythm (for instance, for different genres) that could be evoked in different settings (for example, based on instrument sounds that are associated with particular genres) and that might yield distinct experimental results if participants are primed to rely on one prior or another.

Could the results reflect production-related influences in addition to the perceptual biases traditionally associated with priors? Several existing sources of evidence support a perceptual origin for the experimental result. First, the distribution for US participants is similar irrespective of whether responses are tapped or vocalized (as a repeated syllable), but is markedly different if participants vocally reproduce a spoken sentence^26^. The results are thus not tightly linked to a particular reproduction modality, while also being domain-specific, as would be expected for a prior over musical rhythms. Second, the modes of the prior in US participants confer one characteristic of categorical perception: discrimination of rhythms is worse for rhythms near a mode than for rhythms distant from modes^26^, as would be expected from Bayesian inference^71^. More generally, biases in tapped reproductions^36,88^ are also evident in perceptual discrimination biases^8,89^. Third, the results of our iterated tapping experiment in US participants can be predicted from a Bayesian model with a prior estimated from Western musical scores^90^. This latter result indicates that a prior based on statistics of actual music is sufficient to explain tapping biases, without the need to appeal to any influences specific to motor production.

The present paper provides two additional lines of support for the perceptual interpretation of reproduction biases. The first is that the measured priors predict perceptual category boundaries in a culture-specific way (Extended Data Fig. 7), as would be expected if they function as priors for purely perceptual judgements in addition to production tasks. The second is that priors in musicians were often similar to those in non-musician groups from the same country, despite the greater precision of musician tapping (Fig. 8), providing additional evidence that motor noise does not have much effect on the result.

Despite these various pieces of evidence, we do not think it possible to completely rule out some contribution from motor factors. Given that the estimated priors are similar for tapping and spoken syllables, any motor influences are likely to be central, and in particular also evident even for a production modality (vocalizing) that is not normally dominated by periodic motor rhythms. It may be that completely separating perception and central motor processes is futile for music—listening to music may inevitably invoke motor processes for entrainment^91^, for instance. We note that our conclusions do not depend critically on this point, and that regardless of the perceptual–motor basis, the experiment is measuring something that matters for musical behaviour: the ‘prior’ that we measure provides a description of how what we hear is translated into a reproduction. This process of hearing and reproducing is the essence of musical practice and culture. Our measurement thus has as much legitimacy as anything we might measure with a purely perceptual experiment.

One could also take issue with our selection of participant groups, which were chosen using prior knowledge of local musical cultures to provide a strong test of universality subject to practical constraints. With unlimited resources, one could envision sampling groups uniformly (for example, across geography or linguistic clades) to have the sampling be independent of any particular hypothesis. However, because of the process of cultural globalization, selecting groups in this way would probably produce a prevalence of relatively Western-like participants and would require a much larger number of participant groups to reveal the sort of diversity evident in our results. And given the recent drastic rise of globalization^84^, it is not obvious that uniform sampling would reveal anything fundamental about the diversity of music. Nonetheless, our sampling of groups is clearly not exhaustive, and additional groups might reveal additional constraints on rhythm priors.

Another limitation is that our tests of the influence of local musical idioms were not exhaustive. We related the measured priors to the rhythmic structure of local musical traditions using modes at relatively complex ratios that were known to be prevalent in some cultures and not others (Fig. 7). An alternative approach would be to measure rhythmic structures in corpora of local music and to systematically investigate their relation to each group’s prior. This is not possible at present due to limitations in automated rhythm analysis from recorded music^92^, but it should be possible in the future. We were similarly opportunistic in testing a role for language, and a more comprehensive comparison across a more diverse sample of languages could reveal influences that were too small to detect here.

Finally, previous literature suggests that tempo is an important factor in rhythmic representation^93^. To explore the role of tempo, we ran the experiment with a faster tempo in 13 of the 39 groups (in 6 of the 15 countries). We found that the results for the fast tempo were qualitatively similar to those for the slower tempo used in the main experiment, but there were some instances where category weights changed with tempo (Extended Data Fig. 2 and Supplementary Fig. 1). This finding is consistent with previous work on tempo dependence in rhythm perception but suggests that our main conclusions are not specific to the particular tempo we studied^27,94,95^.

### Relation to prior work

Experimental efforts to compare aspects of music perception across cultures have revealed striking differences along with commonalities^18–30^ but have in all previous cases been limited to comparisons of small numbers of societies. As a result, the existence of universal perceptual phenomena that might constrain music, as well as the extent of cross-cultural variation in perception, has remained unclear. For instance, across a series of studies, one Indigenous society (Tsimane’) was shown to exhibit notable differences in music perception compared with US residents, along with some similarities^25,26,28,29^. These studies have left open the extent to which the particular group studied was unusual relative to other world cultures. Our present results show that at least for the rhythm priors studied here, Tsimane’ listeners are not alone in differing from US listeners—other groups in the study were equally distinct (Fig. 5a). This result suggests that substantial differences with Westerners are unlikely to be limited to a small number of unusual groups.

Our study provides an example of how cross-cultural experiments can be conducted at a relatively large scale. Our method is well suited to cross-cultural experiments in that it is largely non-verbal and simple for participants to understand. It also has the advantage relative to some other methods of making no prior assumption about the structures that might be perceptually important. For instance, small-integer ratios in music have been widely discussed by Western scholars and musicians for centuries^96–99^, and it is natural to wonder whether this emphasis reflects intellectual biases rather than the nature of perception^100^. However, we found small-integer ratios to emerge from the data in each participant group. Small-integer ratios thus indeed appear to be a prominent feature of the way most humans perceive music.

Previous attempts to characterize universality in music have relied on corpus studies that analyse collections of recorded music from different societies^14–16^. Our approach is complementary to such efforts, in that we infer mental representations (from an experiment) rather than directly measure cultural artefacts (music performances). Our method also avoids a weakness of current corpus studies, in that we do not require a Western-trained musical expert to annotate the results, which probably introduces biases that are difficult to quantify or control for. Advances in machine perception are likely to enable corpus studies that automate the transcription process^101^, but biases from the training of such algorithms will probably remain a challenge^102^.

### Future directions

The cross-cultural differences we observed in rhythm priors raise the question of their developmental trajectory. The simplicity of our method should enable cross-cultural experiments in children that could address this. Previous results have suggested that infants initially possess undifferentiated sensitivity to rhythms that then narrow over development dependent on the musical system one is exposed to^53^. Accordingly, priors measured in young children of different societies might be more similar than those in adults. One key question is whether priors are initially uniform or whether small-integer-ratio modes are evident early on. Such experiments could help reveal the origins of the small-integer ratio sensitivity that we found in all groups we tested.

The large-scale, cross-cultural nature of our study illustrates the value of collaboration between science and the humanities, as well as of international cooperation between research groups^47^. To interpret our results, it was essential to consult the ethnomusicological literature and to collaborate closely with ethnomusicologists with a deep understanding of specific musical cultures. Moreover, the clarity provided by a relatively large number of participant groups around the world is most readily attained by large international teams. The results here suggest that large-scale multi-culture studies with other tasks could clarify similar issues of universality and diversity in other aspects of cognition.

## Methods

### Procedure

#### Informed consent

All participants provided informed consent in accordance with the Ethics Council of the Max Planck Society (protocols 2017_12 and 2020_11), the Columbia University Institutional Review Board (protocol IRB-AAAR3726), the University of Western Ontario Health Science Research Ethics Board (protocol 108477), the KAIST Institutional Review Board (protocol IRB-KH2017-15), Durham University (Music Department Ethics Committee, February 2018), the Bogazici University Social Sciences Human Research Ethics Committee (protocol SBB-EAK 2017/1) and the Massachusetts Institute of Technology Committee on the Use of Humans as Experimental Subjects (protocol 1209005242R006). All participants received compensation for their involvement, the amount of which was consistent with the minimum wage regulations of their respective countries. We obtained verbal consent to publish images of participants and musicians.

#### Overview of procedure

The experiment measured iterated reproduction (sometimes referred to as serial reproduction or iterated learning^38,39^) of rhythms. The participants were instructed to synchronize their finger tapping to a repeating auditory stimulus presented over headphones. In previous work^26^ we found that synchronization to an ongoing rhythm produced similar results to an alternative task in which participants heard a pattern and then tapped a reproduction from memory. However, we found empirically that synchronization was easier to explain to participants and for this reason opted to use it for this cross-cultural study. They first completed a short training session (about 10 minutes long) familiarizing them with the apparatus and task (described below). The main experiment consisted of a series of trials, each of which contained five iterations.

On each trial we sampled a random seed uniformly from the triangular rhythm space, corresponding to a three-interval rhythm (*s*_1_, *s*_2_, *s*_3_). We then generated a sequence of clicks from the seed by repeating the three-interval seed pattern ten times. After a few clicks (typically a bit more than one cycle), participants began to synchronize to the click sequence (‘paced’ tapping). A MATLAB script (MATLAB 2018a) extracted response onsets from an audio recording of the participant’s taps (see ‘Onset extraction’ below). We averaged the inter-response intervals across the ten repetitions, obtaining an average three-interval response (*r*_1_, *r*_2_, *r*_3_). Taps were not always detected at every stimulus onset, both because participants sometimes failed to produce a tap in response to a stimulus click, and because produced taps were not detected with 100% reliability. We thus allowed some missing taps within each repeated three-interval pattern but required there to be ‘enough’ taps to estimate an average response. Specifically, we required that each of the three stimulus onsets within the pattern be associated with a tap onset in at least three of the ten repetitions. To obtain the average response, we replaced missing taps with the corresponding average onset time for the taps that were detected. We further required that the response (*r*_1_, *r*_2_, *r*_3_) was not situated far beyond the region we defined for human-producible rhythms (defined as not containing an interval shorter than 285 ms).

If the iteration satisfied these two criteria, we set the seed pattern for the subsequent iteration to the response pattern: (*s*_1_, *s*_2_, *s*_3_) ← (*r*_1_, *r*_2_, *r*_3_). If the iteration was invalid, the data from that iteration were omitted from analysis and the seed remained unchanged. We repeated this process five times. If there were three invalid iterations within a trial, the trial was stopped, and a new trial with a new seed was started (such failed trials typically were due to a rhythm being too difficult for a participant to reproduce, and this procedure was intended to minimize a participant’s frustration). For trials with two or fewer invalid iterations, the *n*th iteration was analysed as the *n*th iteration even if the iteration that preceded it was invalid. There was a fixed interval of approximately 4 s between iterations within a trial and a fixed interval of approximately 9 s between trials (both varied by up to 200 ms in either direction due to slight variation in computer systems across sites).

The number of trials that could be run in an experimental session varied depending on the location and is reported in Supplementary Table 2. In 13 locations we also performed an additional experiment with a faster tempo (a pattern duration of 1,000 ms). This additional experiment was always performed after the main experiment (with a pattern duration of 2,000 ms). The demographic information for this experiment is provided in Supplementary Tables 1 and 3.

#### Apparatus

Data were collected with between one and four computerized stations for each testing location. Each station included a computer, a Focusrite Scarlett 2i2 USB sound card, two sets of Sennheiser HD 280 Pro headphones and a tapping sensor (Fig. 2). This design is identical to that reported in the paper by Jacoby and McDermott that introduced the experiment paradigm^26^. Each sensor contained a microphone embedded in sound isolation materials and covered with a soft cloth to muffle impact sounds as much as possible. Instructions for constructing the sensor are provided in the supplementary Open Science Framework (OSF) repository. The microphone in each sensor was highly sensitive, and light touches generated bursts of noise that were recorded by the microphone. The sound card simultaneously recorded the microphone output and the audio stimulus played out by the participant’s headphones, so that the latency of the response recording relative to the stimulus was nearly eliminated (less than 1 ms). The specification of the hardware and instructions for building the sensor are provided in the OSF repository associated with this paper (see ‘Data availability’).

#### Stimuli

The stimulus on each trial was a rhythmic pattern composed of short percussive sounds (bursts of white noise) 65 ms long with an attack time of 5 ms (linear ramp) and decaying gradually over the remaining 60 ms, with the envelope hand-designed to mimic common percussion instruments (the decay amplitude decreased exponentially to 10% of the maximum over the first 55 ms, then decayed exponentially at a faster rate in the next 5 ms and was then truncated). These patterns contained ten repetitions of a particular three-interval rhythm. The stimuli were identical to those used in Jacoby and McDermott^26^. Software to replicate the experiment and sound material can be found in the OSF repository associated with this paper (see ‘Data availability’).

#### Three-interval rhythms

Each stimulus was defined by a pattern of three intervals (*s*_1_, *s*_2_, *s*_3_) constrained such that the overall pattern duration was 2,000 ms: *s* = *s*_1_ + *s*_2_ + *s*_3_ = 2,000. In addition, to avoid rhythms that were too fast for humans to reproduce, we constrained the initial seeds so that the smallest interval was larger than 300 ms (*s*_1_, *s*_2_, *s*_3_ > 300). We then repeated the interval pattern ten times, thereby forming a sequence of 30 intervals {*S*}_1≤*i*≤30_ = (*s*_1_, *s*_2_, *s*_3_, *s*_1_, *s*_2_, *s*_3_, *s*_1_, …, *s*_3_). From this sequence, we created a sequence of 31 onsets ({*O*}_0≤*t*≤30_) with intervals corresponding to *S*. The 31 onsets (‘clicks’) were defined with respect to the initial onset ${O}_{0},{O}_{t}={O}_{0}+{\sum }_{1\le i\le t}{s}_{i}$ for 1 ≤ *t* ≤ 30.

#### Projection to the rhythm triangle

We mapped a three-interval rhythm with inter-onset intervals (*s*_1_, *s*_2_, *s*_3_) to a point in a triangular rhythm space spanning all linear combinations of three extremal rhythms: $\frac{{s}_{1}}{s}\,{\vec{P}}_{1}+\frac{{s}_{2}}{s}{\vec{P}}_{2}+\frac{{s}_{3}}{s}{\vec{P}}_{3}$, where *s* = *s*_1_ + *s*_2_ + *s*_3_, and where ${\vec{P}}_{i}$ are the vertices of the triangle (a simplex) (Fig. 1b; see also refs. ^9,26^). For visualization, we used an equilateral triangle, with ${\vec{P}}_{1}=\left(\mathrm{0,0}\right),{\vec{{P}}}_{2}=\left(\mathrm{1,0}\right),{\vec{P}}_{3}=\left({\frac{1}{2}},{\frac{\sqrt{3}}{2}}\right)$. Note that since the initial seed intervals satisfied the constraint that (*s*_1_, *s*_2_, *s*_3_) > 300 ms, the initial seeds were located within an inner triangular region with vertices $\left(\,\frac{3}{2}{f},\frac{\sqrt{3}}{2}{f}\,\right),$ $\left(1-\frac{3}{2}{f},\frac{\sqrt{3}}{2}{f}\;\right),\left(\frac{1}{2},\frac{\sqrt{3}}{2}(1-2f)\;\right)$, where *f* = 300/2,000 = 0.15 (Fig. 1b, inner region).

#### Onset extraction

We processed the microphone recording from each trial in non-overlapping windows of 15 s, detecting all samples exceeding a relative threshold of 1.45% of the maximal power of the recorded waveform in the window. This threshold was slightly more sensitive than the 2.25% threshold used in Jacoby and McDermott^26^; this change was made to accommodate a small number of participants (less than 3%) who tended to produce very light taps. In most cases, onsets were detected with minimal errors (as evaluated by comparing the detected onsets to what was audible from listening to example trials). We nevertheless took several steps to ensure that the detected onsets corresponded to actual taps. First, we discarded onsets that were too close to one another (less than 80 ms apart), as humans generally cannot produce two taps in such close proximity (see Repp^3^ for a justification of this threshold). Second, we discarded responses that were too far from any stimulus click, regarding them as errors. Here we took into account an important characteristic of human tapping known as ‘negative mean asynchrony’—namely, that tapping in time to a beat tends to be biased compared with the beat onset (typically occurring before the beat)^3^. We first matched each onset to its closest stimulus click and computed the mean asynchrony *m* as the average difference between a response and its corresponding stimulus click. We then excluded all events such that |(O^R^ − *m*) − O^S^| > 150 ms where O^S^ and O^R^ are the stimulus and response onsets, respectively (namely, a window of 300 ms centred around the perceptual centre defined by the mean asynchrony) from further analysis. In addition, for the analysis, we included only points inside the triangle (see below). This resulted in 99,189 of 2,418,284 tapped responses being excluded from the main experiment (4.1%).

Apart from the change in the detection threshold mentioned above, this procedure was identical to the one reported by Jacoby and McDermott^26^. The code for the procedure is provided in the OSF repository associated with this project.

#### Procedure for the experimental session

The participants were asked to position the sensor in a comfortable way (see the OSF repository for the full instructions). In most cases, participants preferred to sit on a chair, positioning the sensor on their lap and using their entire hand or one finger for tapping. However, we allowed for different postures in different groups. For example, some participants in Mali preferred to sit on the floor with their legs stretched out in front of them. In all cases, the participants were encouraged to change posture or switch hands if they were fatigued; however, they were not permitted to tap with two hands simultaneously or to tap with two alternating fingers. In cases where participants nevertheless tried to do so, the experimenter would stop the experiment and repeat the instructions. The experiment was conducted using a fixed set of steps:

1. The first step of the experiment was to ask the participant to tap ‘a steady beat’ at any tempo that they liked. The idea of this task was to familiarize the participant with the sensor, as well as to characterize the spontaneous motor tempo of the participants^103^. This task was generally easy for participants, but the concept of ‘steady beat’ varied slightly across participant groups. For some participants in Mali and Uruguay, a ‘steady beat’ was a non-isochronous rhythmic pattern rather than an isochronous beat. In cases where participants continued to tap a rhythmic pattern rather than an isochronous beat, the experimenter repeated the instruction. In some cases, participants did not change their behaviour according to the repeated instructions and continued to tap a non-isochronous pattern; in these cases, we did not stop the experiment again and instead proceeded to the next step and recorded their performance as is.
2. The participants were then asked to ‘tap faster’.
3. Next, the participants were instructed to tap ‘as fast as they can’ for a couple of seconds. This step aimed to test that the participant did not have any severe motor constraint that would limit them in performing the task. After the participant tapped for about 3–6 s, the experimenter thanked the participant and stopped their tapping to avoid fatigue.
4. The next step was to familiarize the participant with the stimulus. The experimenter played a few isochronous clicks with an inter-stimulus interval (ISI) of 800 ms over the headphones and asked the participant to report if the sound was too loud or too soft. The level of the sound was then adjusted until the participant felt that the sound was at a comfortable level.
5. The participants were then instructed to tap along to an isochronous beat (with an ISI of 800 ms). This step could be repeated multiple times until the experimenter felt that the participant understood the task and was able to provide a synchronized response. Note that this was not easy for all participants—for example, some participants naturally tapped in antiphase (‘off-beat’; halfway in between the beat). In case of difficulties, the experimenter would first check that this was not caused by the participant’s posture, in which case the experimenter would suggest that the participant change their position to enable easier tapping. In other cases, the experimenter would demonstrate synchronous tapping to the participant. These additional steps allowed nearly all participants to successfully perform isochronous synchronous tapping. The rare participants who could not successfully perform isochronous tapping in this setting did not continue to the main experiment.
6. We then performed an isochronous tapping task at the same rate as in the familiarization phase (ISI = 800 ms) in which the participants tapped to a sequence of 56 clicks lasting 44 s.
7. We next performed an additional isochronous tapping task at a faster rate (ISI = 600 ms) with 75 clicks lasting 45 s.
8. Finally, we performed a tempo-changing tapping task in which the ISI alternated between 546 and 654 ms every 8–13 clicks (chosen pseudorandomly), with a total of 74 clicks lasting 45 s (the exact tapping sequence, identical for all participants, can be found in the OSF repository).
9. In the next step of the experiment, the participants were told that they would now tap a rhythm. The experimenter emphasized that, as before, the participant needed to ‘tap once for every click that they hear’. The participants were given five trials of random three-interval rhythms (each with five iterations) to get them familiarized with the task. The experimenter provided feedback to the participant only if they were performing the task in a qualitatively incorrect way, such as tapping on the off-beats or omitting a beat.
10. The participants performed 10–30 trials (mean, 22.0; s.d., 6.6) of the tapping experiment (each containing five iterations, where each iteration contained ten repetitions of the three-interval pattern, as described above). Due to the long duration, the participants were informed that they could ask for a short break at any time, and the experimenter included additional breaks at various times during the experiment.
11. The participants answered a set of demographic questions (see the OSF repository for the full list) during one of the breaks and/or at the beginning or end of the experiment.
12. In some locations, we performed an additional experiment after the completion of the main experiment, in which we repeated the main experiment with an overall pattern duration of 1,000 ms.

#### Procedures for replicability across sites

Testing stations were created by local research team members according to a set of specifications (see the OSF repository) or created according to the same specifications by N.J. and sent to teams in different locations. A written procedure describing the process of hardware preparation, software installation, task instruction and participant training was delivered to each group (see the OSF repository for the details). The task instructions were translated into local languages. The experimenters were either highly fluent in the local language or accompanied by a translator who was a native speaker. In most locations, the data collection team included an anthropologist or an ethnomusicologist with an expert understanding of the local culture, social groups and music. To ensure that the same procedures were used across sites, all teams were walked through the procedures by N.J., either directly or via video conferencing. Pilot data were collected and analysed at each site, and N.J. inspected the quality of the data before the collection of additional data. To assist this process, the MATLAB script generated images with a small file size (about 150 KB) that summarized the main statistics of data collection (the validity of the trial, the mean and standard deviation of tapping asynchrony (indicative of tapping accuracy) and plots showing the microphone recording levels). The same script also generated small binary files (about 4 KB) with summaries of the data (onset times for stimuli and responses). These files were sent to N.J. via low-bandwidth internet from remote data collection sites, which assisted in troubleshooting technical and data collection errors.

#### Testing conditions

Experiments in the United States (Boston and New York City) were run in sound-attenuating booths. Elsewhere, where possible, the experiments were run indoors in rooms without other activities (Brazil, Uruguay, the United Kingdom, Sweden, Bulgaria, Turkey, the Bamako site in Mali, India, South Korea and Japan). When run outdoors, the experimenter chose a location away from community activities that was relatively free of distractions and noise (Bolivia, the Sagele site in Mali, Botswana, Namibia and China).

#### Demographic questionnaires

We employed a demographic questionnaire to characterize musical experience, dance experience and basic demographic information (age, gender, education and spoken languages). We used a baseline demographic questionnaire (see the OSF repository) that was translated and adapted to different languages and participant groups by the researchers. There was some customization of the questions based on their relevance to the local culture. In each location, we consulted with ethnographers and translators regarding the relevance and translations of each demographic item.

#### Online measurement of tapped responses

To run the experiment online, we used REPP^104^, a software package for measuring sensorimotor synchronization in online experiments that works efficiently using hardware and software available to most online participants. To achieve temporal accuracy superior to that obtained by a web browser (which would have been inadequate for our experiment^105,106^), the software plays the audio stimulus through the participant’s laptop speakers and records the original signal simultaneously with the participant’s responses (which they supply by tapping on the laptop case) using the built-in laptop microphone. The resulting recording is then analysed to extract the participant’s taps. The method has been validated in a series of calibration and behavioural experiments^104^, and it achieves high temporal accuracy (latency and jitter within 2 ms on average). In addition, the method has been shown to provide results that are consistent with those obtained in a laboratory set-up (for example, for isochronous tapping, the lab–online correlation for the tapping precision of individual participants was measured to be *r*_19_ = 0.94; *P* < 0.001; 95% CI, (0.85, 0.98); see Experiment 2 in ref. ^104^).

To measure stimulus-coordinated tapping by online participants, we made some modifications to the original paradigm. One set of differences involved the stimulus. First, because the recorded audio contains both the stimulus and the response, we filtered the stimulus with a high-pass filter (cut-off frequency of 1,000 Hz) to avoid overlap with the frequency range typically occupied by tapping responses (80–500 Hz). Next, we added three custom audio ‘markers’ with known temporal locations at the beginning and end of each stimulus (six in total). These markers enabled us to unambiguously identify the positions of the stimulus onsets in the recorded audio and facilitated precise measurement of participants’ asynchronies. The markers were designed to be robustly detected across a variety of hardware and software set-ups, including cases of noise-cancellation technologies and ambient room noise. The marker sounds were generated from 15 ms bursts of bandpass-filtered white noise in the range of 200–340 Hz, to which we applied linear ramps at the onset and offset (2 ms long). We chose very short intervals between the markers (280 ms for the first interval and 230 ms for the second) to avoid participants confusing the markers with the repeated rhythm (the rhythm pulses and the markers also differed substantially in timbre due to the different frequency ranges).

A second set of differences involved the response recording. The online experiments used free-field recording whereby the audio stimulus is played through the laptop speakers and simultaneously recorded along with the participant’s tapping response using the built-in laptop microphone. This returns an audio file where both the audio stimulus and participant tapping are superimposed. To separate this recording into the different components of the stimulus and response, we used bandpass filters. Since most of the energy in the tapping signal occurs at low frequencies, filtering the recording around the tapping range (80–500 Hz) isolated the tapping response with a high degree of efficacy. We were also able to isolate the markers by filtering in their frequency range (200–340 Hz). In addition, we applied a filter in the 100–170 Hz range, the output of which was used for calibration. Because the markers had no energy in this range, this helped determine the noise level in the recording, which we used to adaptively set the marker detection thresholds. This allowed us to reliably estimate the marker locations even with very noisy or low-quality recordings, as characterize some laptop models and brands.

The detected stimulus markers were used to estimate and compensate for the latency of the recording and to estimate the jitter in the recording. This enabled us to monitor the timing accuracy of each individual trial, crucial to ensuring that timing accuracy remained high in all trials. See Experiment 1 in Anglada-Tort et al.^104^ for the full details of the calibration experiments and their validation.

After the online-specific pre-processing to isolate the tapped response and compensate for the recording latency, we used the same pipeline as in the main experiment to align the tap onsets and perform the tapping analysis.

#### Online iterated reproduction procedure

To meet the challenges of online data collection, such as poor control over participants’ hardware and software and a higher risk of fraudulent responders, we made two minor changes to the iterated reproduction procedure.

1. In the online version of the experiment, the analysis of the recording could take a few seconds to complete (for example, from uploading the audio recording, performing the signal processing and synthesizing the new stimulus). To avoid unnecessary wait times, we did not run consecutive iterated reproductions of the same rhythm seed, as in the laboratory. Instead, we ran six ‘chains’ of iterated reproductions in parallel. On each trial of the online experiment, the participants performed a single iteration of a chain (that is, tapping to a single ten-repetition rhythm—either a seed rhythm or the result of the reproduction from the preceding iteration of the chain). Each trial was randomly assigned to one of the chains that was not used for the previous trial. Each participant completed all six chains during an experimental block. In Jacoby and McDermott (Experiment 4)^26^, we showed that the results of this parallel chain procedure do not differ substantially from those of the original paradigm, where the iterations from different chains are not intermixed.
2. The change in trial order also necessitated a change in the failing criteria: if participants failed a trial, they repeated the trial with a maximum of ten possible additional attempts, but these repeated trials were randomly drawn from the six chains (the ten allowed failed trials were tallied globally within the block). In those cases where this limit was exceeded, the experiment was terminated.

In addition, the following seven modifications were made to the overall procedure to ensure that the online participants met the technical requirements for the online experiment and were able to provide good tapping data consistently throughout the experiment. The different steps in this procedure have been extensively piloted and optimized to ensure high data quality when collecting tapping data in online settings^104^.

1. First, the participants were instructed that the experiment could only be performed using the laptop speakers and that they should unplug any headphones/earphones or disconnect any wireless devices. They were also instructed to remain in a quiet environment.
2. The participants were then asked to set the volume of their speakers to a level that was sufficiently high to be detected by the microphone. A sound meter was used to visually indicate when the level was appropriate.
3. After the volume test, the participants completed a short recording test to detect hardware and software that did not meet the technical requirements of the experiment, such as malfunctioning speakers or microphones. The recording test played a test stimulus with six marker sounds. The markers were recorded with the laptop’s microphone and analysed using our signal processing pipeline. During the playback, the participants were supposed to remain silent. There were a total of three such test recording trials, and we provided feedback after the first trial based on the recording quality: if the markers were not recorded (for example, this could occur if the participant forgot to unplug their headphones), we reminded the participants that they needed to unplug any headphones. If, despite these reminders, marker sounds could not be detected in two of the three trials, the participant was excluded from the experiment. Note that this process also serves as a basic test of task compliance, as the participants must follow the instructions (for example, accept the enabling of the microphone in the browser, unplug any headphones or wireless devices and adjust the volume of the computer) to pass the test. Participants who did not satisfy the technical conditions or who abandoned the experiment at this stage were excluded (747 of 1,303). This relatively high percentage of participants that did not satisfy the technical inclusion criteria is consistent with previous online tapping experiments^104^.
4. Participants who passed the recording test were then directed to a tapping calibration test. Here, the participants were asked to tap on the surface of their laptops with their index finger to test whether the microphone could detect their taps, using a sound meter to visually provide feedback. In cases where the signal was too low, the participants were asked to tap on different locations of the laptop or to try to tap harder.
5. Next, the participants performed a practice phase to acquaint themselves with the main tapping task. The practice phase consisted of four trials using the stimulus sampling procedure of the main experiment (for example, three-interval rhythms randomly sampled from the triangular simplex with a fixed duration of 2,000 ms and repeated ten times). In the first two trials, we provided feedback to the participants based on their recording quality and tapping performance. We used the remaining two trials to exclude participants who were still unable to provide good tapping data, as assessed by failing in one or more of these two trials. A trial was considered a failure if we could not detect all marker sounds, or if the detected markers were displaced relative to each other by more than 15 ms, or if the percentage of detected taps (that is, the number of detected tapping onsets out of the total number of stimulus onsets) was less than 50% or more than 200%. Note that none of these criteria involve participants’ accuracy in replicating the target rhythm; they only reflect whether the signal could be correctly recorded and processed, and whether the participants produced a minimally/maximally acceptable number of tapping responses. An additional 358 participants were excluded on this basis.
6. Participants who passed the practice phase were then able to start the main tapping task, which used the same procedure described for the in-person experiments except for the modifications mentioned above.
7. As mentioned above, a main difference between the in-person and online experiments was that the latter consisted of shorter and more flexible experimental sessions. Namely, the experiment was divided in different blocks of six chains per block. After completing one block, the participants could decide whether to continue with the next block or to instead end the experiment. There was a maximum of three blocks per session (18 chains). The motivation behind this design choice was to keep online experimental sessions engaging and short, always allowing the participants to decide whether to complete more trials or not.

After completing the first block, the participants answered the same set of demographic questions used in the in-person experiments. We excluded participants who abandoned the experiment prior to its completion or who did not complete the full demographic questionnaire that we administered at the end of the experiment (67 additional participants were excluded on this basis). In total, 131 online participants completed the full experiment and were analysed.

### Participants

We tested 39 participant groups spanning five continents and 15 countries (Extended Data Table 1). Overall, we recruited 923 participants (792 were run face-to-face and 131 online) who completed a total of 20,287 trials (seeds) and 2,319,095 individual taps.

#### Criteria for group selection

Participant groups were chosen to provide a strong test of any potentially universal features of the results. We included groups from both industrialized and non-industrialized societies, as well as groups of local musicians from some non-Western societies (who performed different types of non-Western music). We also tested groups of musicians and dancers where possible, as these populations would be expected to have substantial exposure to particular musical styles. In addition, we tested university students in a number of countries to assess potential effects of exposure to Western culture, which we presumed would be correlated with university attendance. The groups tested were also determined in part by practical constraints (testing time and access to particular populations). Age and gender could not be fully equalized across groups. For example, Malian professional jembe drummers and Uruguayan candombe drummers (the populations recruited for MA-LM and UY-LM) are both relatively small groups—less than 50 individuals—composed of highly skilled professionals, and were predominantly male. In both cases, only one participant in each group was female. The substantial experience required for membership in these groups also resulted in these participants being older (Mali: mean age, 40.5 years; s.d., 11.9; Uruguay: mean age, 45.5 years; s.d., 12.8). At the other extreme, dancers in the Sagele village in Mali (MA-DA) were predominantly female.

#### Sample sizes

We conducted a power analysis using data from US participants collected for a previous publication^26^. The approach was to try to collect enough trials that the test–retest reliability of the estimated prior for a group was likely to be relatively high (with the goal of having enough data that the results of the experiment would be similar in a hypothetical future replication). The test–retest reliability was estimated using the split-half reliability of our previously collected data following Spearman–Brown correction^107^. We simulated different amounts of data by subsampling the number of trials used to estimate the prior (resampling without replacement). We found that 250 trials produced a test–retest reliability greater than 0.8, and we thus targeted this number for the sample size of each group. In practice, we often ran more trials if circumstances permitted (between 261 and 948 trials and therefore up to 3.8× the target number of trials; Supplementary Table 2). The only exceptions were the two groups in Botswana, for whom we did not reach this recruitment target because of practical constraints on testing time (170 and 127 trials for the San and Etsha groups, respectively; abbreviated as BO.SA and BO.EA). However, the post hoc reliability of the data collected for these groups was not far below our target value (0.75 and 0.67 for BO.SA and BO.EA, respectively). The post hoc reliabilities of all other groups were high, meeting or exceeding the predicted value of 0.8 (ranging from 0.8 to 0.96; mean, 0.9; s.d., 0.03).

### Definition of group types

#### Students (ST)

We defined students as members of local universities in either undergraduate or graduate programmes.

#### Musicians (WM and LM)

For brevity, we used the term ‘musicians’ to describe participants with relatively extensive musical experience, acknowledging that musicianship is a concept that changes from place to place^47^. On the basis of previous work, we defined recruiting criteria that generalize more broadly for different cultural contexts^27^: (1) professionalism—‘Do you make most or part of your living from music, or did you in the past?’, (2) training—‘Did you undergo music training (such as an apprenticeship or formal study)?’ and (3) public playing—‘Do you perform in public?’ In some locations (the United States, Uruguay, the United Kingdom, Bulgaria, Turkey, Mali and Namibia), all musicians satisfied all criteria, while in other locations (Brazil, Sweden, Botswana, India, Korea and Japan), we also included people who satisfied the last two criteria but not the first one. For all participants, we recorded self-reported years of regularly playing an instrument or singing, as is common in music cognition literature (Supplementary Table 2). We recruited both musicians who play Western classical music (WM) and musicians who play a local musical style that is not Western classical music (LM). We note that for the local musician groups, the nature of the local musical style varied somewhat from group to group; in some cases (most notably for the US.NY-LM group, who played jazz) the musical style was one that had spread globally.

#### Dancers (DA)

In Bulgaria, we recruited dancers who were members of the same professional ensembles from which we recruited the musician groups. In Mali, we recruited group members of a local recreational dance association that promotes events featuring traditional dance and music.

#### Non-musicians (NM)

Non-musicians were people who did not satisfy any of the inclusion criteria for the other groups. Their self-reported years of musical experience were substantially less than those of the musician groups in all cases (Supplementary Table 2).

#### Online (OL)

Online participants were recruited from Amazon Mechanical Turk. Their geographical location was determined by the Amazon qualification system and verified with IP geo-location. We tested participants from the 3 countries of the 15 from which the other participant groups were drawn with substantial Mechanical Turk worker pools (the United States, Brazil and India)^108^.

### Recruiting locations

Basic demographic information for each participant group is provided in Extended Data Table 1. Supplementary Table 2 provides additional information about each group including the number of languages spoken, the languages spoken, years of self-reported musical experience, instruments played and favourite artists or musical genres. We report demographic variables that we were able to reliably measure, and we note that these are not the only variables that varied across groups and that might influence the results. These factors were all based on self-report questionnaires, except the literacy level within the group, which was estimated by the experimenters. Here we provide additional information about each group, ordered according to their geographical location.

#### United States: Boston—students and Western classical musicians (US.BO-ST and US.BO-WM)

The Boston student participants were students from local universities, recruited using the MIT Brain and Cognitive Sciences Department participant mailing list and through additional online advertisement. All participants were residents of the Boston area, a metropolitan region of New England with over eight million inhabitants. The musician group was recruited from the same departmental mailing list as well as via a social media ad targeting conservatory students from the Boston area. All participants in this musician group had formal training in music. Some of them were professional musicians. Most participants in the musician group played Western classical music, though some also played other styles such as pop and jazz. There was some overlap between these groups and the US participant group in a previous publication^26^; the groups were not identical due to different exclusion criteria in the two studies.

#### United States: New York City—non-musicians, Western classical musicians and jazz musicians (US.NY-NM, US.NY-WM and US.NY-LM)

New York participants were recruited by word of mouth, campus advertisements at Columbia University and online advertisements. The participants were residents of the New York City metropolitan area, a densely populated region in the United States with over 18.8 million people. We recruited three groups: non-musicians, musicians specializing in Western classical music (the WM group) and jazz musicians (the LM group). Both musician groups were a mix of music students and professional musicians. All musicians had formal education and training in music.

#### Bolivia: Tsimane’—non-musicians (BO.TS-NM)

Tsimane’ are an Indigenous people of lowland Bolivia, comprising about 19,000 individuals who live in about 130 small villages mostly along river basins (including the Maniqui River), located in the department of Beni (a subdivision of Bolivia). They subsist mostly on farming, fishing and hunting. Tsimane’ have traditional music, familiarity with which varies across individuals. As reported by Riester^109^, their traditional songs have characteristic rhythmic patterns. The most common such pattern reported by Riester can be written in ratios as 1:1:2. Traditional Tsimane’ musical culture also once included shamanic practices with drum playing, but these practices are no longer in use^110^. The region containing Tsimane’ communities is undergoing rapid modernization due to a push by the Bolivian government and non-government organizations to provide services to the Indigenous peoples. Radio usage is now fairly common, and villages near the local town of San Borja tend to have electricity. During the mid-1950s, Protestant missionaries from the United States settled permanently along the river Maniqui to proselytize Tsimane’, setting up the first rural schools for them and teaching them church hymns^111^. More recently, evangelism has spread Christianity within and across many villages. Thus, in addition to their knowledge of traditional music, nowadays most Tsimane’ villagers are somewhat familiar with religious Christian hymns. These hymns are monophonic and sung in Tsimane’. They are similar to traditional Tsimane’ music in relying on small intervals and a narrow vocal range and are sometimes accompanied by other instruments played by community members. Group singing appears to be rare, irrespective of whether the material is traditional songs or hymns. For the present study, we recruited participants in three Tsimane’ villages. Upon arriving at each village, we used a horn or a bell to initiate a community meeting where we introduced the researchers and registered participants for experiment sessions. Two of the villages (Mara and Moseruna) were a two-day walk or a three-hour car ride from San Borja, along a road that was accessible only to high-clearance vehicles and motorcycles if recent weather had been dry. The other village (Yaranda) was located along the Maniqui River and accessible only by a one-day trip on a motorized canoe. All three villages have relatively little communal church singing. The participants had varied musical experience, but none regarded music as a profession. None of the participants had formal training in music. Two participants reported regularly playing an instrument, and 34 participants reported playing an instrument at least once.

#### Bolivia: San Borja—non-musicians (BO.SB-NM)

San Borja is a small town in the Bolivian department of Beni in the Amazon basin with over 20,000 residents. At the time of data collection, San Borja could be reached by car during dry months of the year but was accessible only by small planes during much of the rainy season. Participants were recruited by word of mouth and had resided for most of their life in San Borja. The participants had not received formal education in music.

#### Bolivia: Santa Cruz de la Sierra—non-musicians (BO.SC-NM)

Santa Cruz de la Sierra is the largest city in Bolivia, with a population of over 1.8 million people. Participants were recruited using an online advertisement and word of mouth. All participants had been born and raised in the Bolivian department of Santa Cruz, and they resided in the city at the time of the experiment. The participants had not received formal education in music.

#### Bolivia: La Paz—students and non-musicians (BO.LP-ST and BO.LP-NM)

La Paz is the third-largest city in Bolivia, with a population of over 0.9 million people, located in the Andes. We recruited the student group from local universities. Most student participants were recruited by a student who was a research assistant. For the non-musician group, we recruited blue-collar workers employed by a hotel and their relatives. These participants were mostly from El Alto, a city adjacent to La Paz (with a population of about one million people), and many of them were Indigenous (Aymara or Quechua). Many participants reported experience with traditional music and dance in childhood or as adults. The participants had not received formal education in music.

#### Brazil—local musicians (BR-LM)

We recruited percussionists in the Recife metropolitan area, a city in Pernambuco, part of Northeast Brazil. The population of the Recife metropolitan area is over four million people. The percussionists practice a local style of music that is part of the *Maracatu-nação* (translation from Portuguese: ‘nation *maracatu*’) cultural tradition. This region-specific tradition, usually perceived as Afro-Brazilian, involves religion, music, song, dance and elaborate costumes. Its history is disputed but is most often linked to the colonial coronations of queens and kings among enslaved Africans in Brazil (in the sixteenth to nineteenth centuries). However, the earliest descriptions date only from the beginning of the twentieth century^112,113^. The music is performed by community-based groups in the slums of the metropolitan area of Recife termed *maracatus-nação*, approximately 30 of which are currently active. These groups include a costumed dance group and a large percussion ensemble. The groups perform in parades that take place primarily during Carnival. The percussionists we recruited either grew up within one of these community groups or had participated in one for at least several years. The participants had not received formal education in music in a university or conservatory but received substantial training as described above.

#### Uruguay—students and local musicians (UY-ST and UY-LM)

Participants in Uruguay were all recruited in Montevideo, the capital of Uruguay, with a population of over 1.3 million people. The musicians were performers of Uruguayan candombe drumming^59,114^. All participants were born and raised in neighbourhoods with a strong tradition of candombe drumming and were ‘native players’, having acquired their competence in the style by direct transmission. The majority of the participants were outstanding players, regarded as master drummers by the community. As a rule, the participants did not have formal musical training in a conservatory or university, although a few of them had some basic knowledge of music theory and could play instruments other than the drum (such as keyboards and bass). However, they had all had substantial practical training in drumming since childhood. The participants were recruited by L.J. and M.R., who are local experts in this musical style and are familiar with the local musicians. The students were members of the local university with no formal musical training but with passive exposure to music, including Uruguayan candombe drumming. They were recruited by two research assistants by word of mouth.

#### United Kingdom—students and jazz musicians (UK-ST and UK-LM)

Two groups of participants were recruited in North East England and Scotland, an area of the United Kingdom with over eight million people. Most participants were recruited from Durham, a county in England with over 500,000 people. The first group consisted of students from Durham University. The second group consisted of instrumental jazz musicians, comprising a mix of professional musicians and students currently studying music at university (the students were recruited from Durham University and the University of Edinburgh). All participants in this group reported that they perform jazz in public and earn money from performing, and all had formal training in music in a university and/or conservatory. These participants played a range of instruments (including piano, saxophone, guitar, trumpet, drums and double bass), and most reported performing in a range of different sub-genres and groups, most commonly big bands and smaller ensembles (such as trios) playing jazz standards. Some participants reported liking or performing musical traditions from other cultures, including Latin and Balkan-influenced music.

#### Sweden—local musicians (SE-LM)

The recruitment of musicians in Stockholm (the largest metropolitan area in Sweden, with over 2.4 million people) focused on students and teachers of folk music performance at the Royal College of Music, and on dance students at the School of Dance and Circus at the Stockholm University of the Arts, as the latter also had extensive experience playing music. Among the latter group, we required the participants’ focus to be Swedish folk dance. Of the 22 recruited participants from the Stockholm area, 9 considered themselves mainly as dancers and 13 mainly as musicians, and 1 participant identified as a dancer and musician to the same degree. In addition, 72% of the participants reported making money from performing music or dance, and all but 4 reported performing in public. Independent of this self-categorization, all participants asserted that they dance and have experience with either instrumental or vocal music making.

#### Bulgaria—local musicians and dancers (BG-LM and BG-DA)

The recruitment of musicians and dancers in Bulgaria focused on members of a type of professional folk ensemble that developed once the country adopted a communist system of government after the Second World War^115,116^. These ensembles typically consist of an orchestra of folk instruments, a women’s choir and a dance troupe, and they give stage performances of arranged and newly composed Bulgarian folk music and elaborately choreographed folk dances. Most performers in these ensembles have studied folk music performance or choreography in the Bulgarian conservatory system. Some of the dancers who participated in the study belonged to professional or semi-professional dance troupes that perform Bulgarian folk dances with recorded rather than live music. We recruited participants in three Bulgarian cities: Pleven (a city with approximately 90,000 people), Plovdiv (a city with approximately 343,000 people) and Sofia (the capital of Bulgaria and the largest metropolitan area, with over 1.2 million people). The participants were members of the ensembles in these cities and were contacted with permission from the ensemble directors.

#### Turkey—students and local musicians (TR-ST and TR-LM)

The group of Turkish student participants was recruited from Istanbul Technical University Turkish Music State Conservatory and Bogazici University. The Istanbul metropolitan area has over 15.8 million people. The musician participant group was recruited from the cities of Izmir and Istanbul and included professional musicians who had studied at institutes or conservatories for traditional Turkish music, such as Istanbul Technical University Turkish Music State Conservatory. These musicians were experienced in Turkish folk music or dance through formal training or extensive practice in groups or ensembles. They had experience in various sub-genres of Turkish music, ranging from Aegean to Black Sea regions, also including Sufi music. The musicians were involved in ensembles performing traditional, religious, classical or modern Turkish music, as well as Western music with Eastern influences.

#### Mali—students, local musicians and dancers (MA-ST, MA-LM and MA-DA)

The group of Malian university students was recruited in the capital city of Bamako and comprised both BA- and MA-level students as well as recent graduates of the University of Bamako. Bamako is the capital of Mali and the largest metropolitan area in the country, with a population of over two million people. Students were recruited by N.D. by word of mouth. The musician group was also recruited in Bamako. Their main performance work occurred at local music and dance events, primarily wedding celebrations, but they also worked in the national and international scenes of staged folk dance and percussion music^117^. Most musicians did not have training in music from a university or conservatory but instead had substantial training via traditional, practice-based apprenticeships. By contrast, Malian dancers were not active as specialized musicians (and did not have formal education in music or dance) but regularly danced at wedding celebrations in which professional musicians performed, and thus were highly familiar with local styles of music. Only one of the participants reported receiving money from playing or dancing. R.P. (who has more than 30 years of experience working with Malian musicians) recruited the musicians with the help of a research assistant. The dancer participants were recruited on the basis of their membership in a local dance organization in the peasant village of Sagele, approximately 75 km southwest of Bamako. Sagele has a population of approximately 5,000 people.

#### Botswana: San—local musicians (BW.SA-LM)

The San musician group was recruited in D’Kar, a village ~640 km northwest of Gabarone (the capital of Botswana), with a population of about 1,700 people. The participants were either members of a local organization that performed traditional songs and dances of the San culture (primarily for nearby events or for tourists) or local residents with substantial musical experience (at least ten years of self-reported musical experience) but no formal musical training in a university or conservatory.

#### Botswana: Etsha—local musicians (BW.EA-LM)

The second Botswanan group was recruited in Etsha, a group of villages ~320 km north of D’kar, with a population of about 10,000 people. The participants were members of two groups who performed traditional songs, or local residents with substantial musical experience (more than seven self-reported years of musical experience). The participants came from two closely related Okavango Delta region subcultures: Hambukushu and Bayei. Although the Hambukushu and Bayei are culturally distinct, their geographic proximity means they are each exposed to each other’s music, with far less exposure to the music of the San. The participants had no formal musical training from a university or conservatory.

#### Namibia—non-musicians and local musicians (NM-NM and NM-LM)

Participants in Namibia were recruited from a small local Damara community (population ~500) in the Spitzkoppe region via word of mouth during the week prior to the start of the study. Recruitment and data collection occurred with help from two local research assistants, who also acted as translators. The musician group was drawn from two active musical groups, both of whom had extensive experience with traditional music of the region: a ‘cultural singing group’ (who frequently practice and perform *|ais* (old traditional folk songs and dances), in addition to other styles of performance) and a ‘youth choir’ (who typically rehearse and perform *elob mis*, a form of gospel music, sung in Khoekhoegowab (the local language), English and Afrikaans). Both groups perform at local and regional community events (such as weddings, funerals and an annual Damara traditional festival). The singing group intermittently performs for visiting tourists, and the youth choir also performs weekly in church. The first group typically earns money in exchange for these performances, though most members would not be construed as professional performers and did not have formal training from a university or a conservatory. The non-musician participants were members of the local community who were not part of either group. Although almost all participants reported engaging in some form of singing (for example, joining in at weddings or other community events, or simply while listening to the radio), these non-musician participants did not regularly practice or perform with any group, and many had limited or no knowledge of old traditional song lyrics and dances.

#### India—non-musicians and local musicians (IN-NM and IN-LM)

In India, the experiment was conducted at I.I.T. Bombay in the city of Mumbai (a city with over 20.9 million people). The non-musicians all worked or studied at I.I.T. Bombay and had not had any musical training or substantial exposure to Indian classical music. We classified the group as non-musicians rather than students because only a minority (6 of 15) were students. Most of the musician participants were professional musicians living in the city, but a few were students at I.I.T. Bombay. Most of them were trained in the North Indian (Hindustani) form of art music. Formal training typically involves taking one-on-one lessons from a teacher (the appointed guru) and performing in public concerts. Some of the musician participants were primarily vocalists but also played an instrument, and about half of them were tabla (percussion) players who accompanied vocalists in concerts. All but three musicians reported currently playing in public concerts, and 64% reported at least sometimes receiving money from playing.

#### South Korea—students, Western classical musicians and local musicians (KR-ST, KR-WM and KR-LM)

The Korean student participant group was recruited from Chungnam National University in Daejeon, a metropolitan area containing over 1.4 million people. The Western musician group consisted of students from the Department of Music at Chungnam National University. These participants had been exposed to both Western music and K-pop music via mass media. The local musician group comprised students studying traditional Korean instruments in the Department of Korean Music at Jeonbuk National University located in Jeonju, a metropolitan area containing over 651,000 people. These participants had trained on Korean traditional instruments for many years but also had extensive exposure to both Western and K-pop music.

#### Japan—students, Western classical musicians and local musicians (JP-ST, JP-WM and JP-LM)

In Japan, the student and Western musician groups were recruited from Keio University Shonan Fujisawa Campus in Fujisawa, Kanagawa, near Tokyo. Tokyo is the capital of Japan and the metropolitan area with the largest population (over 37.2 million people). Participants in the student group had no formal musical training. The Western musician group had formal music training in Western instruments. The local musician group was recruited from Tokyo University of the Arts (Japan’s leading music conservatory). They all played at public events, and 73% received money for playing music. We recruited students studying traditional Japanese instruments (*shamisen*, *koto*, *shakuhachi* or *hayashi* instruments from the *noh* ensemble) in the Department of Traditional Japanese Music. These students had to train on these instruments for many years to qualify for acceptance into the department and can be considered ‘bi-musical’^118^ in that they had extensive exposure to both popular Western and traditional Japanese musical systems. All Japanese participants had extensive passive exposure to both Western music and Western-influenced Japanese popular music via radio, TV and other media. All Japanese participants were recruited from Tokyo and its surrounding cities.

#### China—non-musicians (CN-NM)

Participants in China were recruited from a cluster of Dong minority villages in the Guizhou Province in southwestern China. The Guizhou Province contains approximately 38.5 million people, and the Dong villages each have approximately 500 to 2,000 people. Singing features prominently in many village activities, but the most famous and distinctive tradition of Dong song is *Dage*, or Big Song, recognized by UNESCO in 2009 on their list of humanity’s Intangible Cultural Heritage. There is wide participation throughout the villages in learning and performing *Dage*, a genre of two-part group polyphonic singing, occasionally metred, where words are central and pitch height and contour carefully follow the lyrics^119^. *Dage* is performed informally within people’s homes and more formally within the drum-towers found in many villages^119^. The participants had no formal musical training from a university or conservatory.

#### Online participants from the United States, Brazil and India (US-OL, BR-OL and IN-OL)

All online participants were recruited using Amazon Mechanical Turk. In the online advertisement, we required the participants to meet the following five criteria: they had to (1) be at least 18 years old, (2) speak English, (3) use a laptop to complete the experiment (no desktop computers allowed), (4) use an up-to-date Google Chrome browser (due to compatibility with the technology) and (5) sit in a quiet environment, to ensure that their tapping could be recorded precisely. To test participants from the United States, India and Brazil, we used Amazon Mechanical Turk’s qualification system, which allows researchers to recruit participants registered in each location. To ensure that the participants were undertaking the experiment from the registered location, we only included participants whose registered location matched their IP-based geo-location, which we verified using the service ipinfo.io. We estimate that the Mechanical Turk participant pool in the United States has a few thousand unique active participants per week. In India and Brazil, we estimate that the number of unique active participants is about 500 and 200, respectively.

### Analysis

#### Small-integer ratios

Following Jacoby and McDermott^26^, we considered ‘small-integer-ratio rhythms’ to be those with ratios composed of the integers 1, 2 and 3 that fell within the rhythm triangle. This results in the following 22 unique ratios: *Ω*_22_ = {1:1:1, 1:1:2, 1:2:1, 2:1:1, 1:2:2, 2:1:2, 2:2:1, 1:1:3, 1:3:1, 3:1:1, 1:2:3, 2:3:1, 3:1:2, 1:3:2, 2:1:3, 3:2:1, 2:2:3, 2:3:2, 3:2:2, 2:3:3, 3:2:3, 3:3:2}. We also grouped together categories that are equivalent under cyclic permutation, resulting in eight categories: *Ω*_8_ = {111, 112, 122, 113, 123, 223, 233, 132}.

#### Kernel density estimates of the prior

The experiment consisted of a number of trials, each of which consisted of iterated reproduction of a random seed rhythm. We estimated a participant’s prior using the data from the fifth and final iteration of each trial, having demonstrated in Jacoby and McDermott^26^ that five iterations are sufficient for the iterative procedure to converge to the prior (Supplementary Figs. 1, 2 and 7 in Jacoby and McDermott; see Supplementary Fig. 2 of this paper for analyses of convergence in each group)^26^. Before this analysis, we excluded points outside the inner triangular region defined earlier that was intended to correspond to the region of human-producible rhythms (with vertices $\left(\,\frac{3}{2}{f},\frac{\sqrt{3}}{2}{f}\,\right),\left(1-\frac{3}{2}{f},\frac{\sqrt{3}}{2}{f}\;\right),\left(\frac{1}{2},\frac{\sqrt{3}}{2}(1-2f)\;\right)$, where *f* = 300/2,000). The prior was then estimated by adding together Gaussian kernels, with mean *μ*_*i*_ and covariance *Σ*_*i*_ empirically computed from the repetitions of the rhythm within the fifth iteration (there were up to ten repetitions depending on the number that the participant correctly produced during the fifth iteration; for repetitions that had missing taps, the missing tap(s) was replaced by the mean onset of the successfully produced taps at that stimulus position). Since this covariance matrix is estimated on the basis of small numbers of samples, we added a regularization term: ${\varSigma }_{i}^{{\prime} }={\varSigma }_{i}+\gamma I$, where *I* is the identity matrix, and *γ* = 15 ms (we slightly increased this value compared with the *γ* = 10 ms of Jacoby and McDermott^26^ since some participant groups had lower numbers of correctly reproduced repetitions). We averaged these kernels (${G}_{i}\left(x\right) \sim {\mathrm{N}}({\mu }_{i},{\varSigma }_{i}^{{\prime} })$, one per trial) across all completed trials within a participant group, obtaining a distribution $P(x)=\frac{1}{N}{\sum }_{i=1}^{N}{G}_{i}\left(x\right)$ over the triangle. For statistical analyses, we represented these distributions in bins spanning 0.006 in each dimension of the triangle (that is, 12 ms given the 2,000 ms pattern duration). To generate high-resolution images for the paper figures, we used bins of size 0.003 in each dimension. Supplementary Figs. 3–9 show high-resolution kernel density estimates for all groups.

#### Normalizing density compared with uniform distribution

As described above, the random seeds were constrained to have the smallest interval exceed 15% of the pattern duration (300 ms), corresponding to a smaller triangular region within the full rhythm triangle. We defined the uniform distribution over this smaller region as *U*. To avoid working with small numbers, we pointwise-normalized the kernel density estimate *P* with respect to *U*—namely, *P*′(*x*) = *P*(*x*)/*U*(*x*). We note that in all images depicting kernel density estimates, the density was clipped at a value of 5 (relative to uniform) to preserve the dynamic range for details at low density values. In the OSF repository associated with this project, we included images with less clipping (relative density of 10).

#### Jensen–Shannon divergence

To compare distances between distributions, we used the Jensen–Shannon divergence. The Jensen–Shannon divergence of two distributions *P* and *Q* is defined as

$${\mathrm{JSD}}(P,Q)=\frac{1}{2}{D}_{{\mathrm{KL}}}\left(P,M\,\right)+\frac{1}{2}{D}_{{\mathrm{KL}}}\left(Q,M\,\right)$$

where $$M=\frac{1}{2}\left(P+Q\right)$$ and $${D}_{{\mathrm{KL}}}\left(P,Q\right)=\displaystyle\int_{x} P(x)\ \log_2\left(\frac{P(x)}{Q(x)}\right)\ {\mathrm{d}} \ \!x$$.

#### Gaussian mixture model fits

To measure the relative weight of each category in a group’s prior, we used a Gaussian mixture model in which the mean of each mixture component was constrained to be close to a small-integer-ratio rhythm. This constraint aided interpretability by removing the degeneracy in the correspondence between mixture components and the modes of the data distribution, guaranteeing that each mode was associated with the same category across groups, while allowing the mixture components to deviate from exact integer ratios as dictated by the data. We imposed additional constraints on the standard Gaussian mixture model fitting procedure of the model to ensure that the mapping of mixture components to integer ratios was fixed across groups, to avoid artefacts associated with small sample size and to avoid uninterpretable overlap between the modes.

We define a Gaussian mixture model with category centres {*μ*_*i*_}_*i*=1…*K*_, covariance matrices {*C*_*i*_}_*i*=1…*K*_ and weights {*w*_*i*_}_*i*=1…*K*_ as follows:

$$Q(x)=\sum _{i}\frac{{w}_{i}}{2\pi \sqrt{{\rm{|}}{C}_{k}{\rm{|}}}}\exp \left(-\frac{1}{2}{\left(x-{\mu }_{i}\right)}^{{\mathrm{T}}}{C}_{k}^{-1}(x-{\mu }_{i})\right)\,$$

To fit the model, we used a modified expectation–maximization algorithm^120^. We initialized the algorithm by assigning the mixture components to the small-integer-ratio rhythms within *Ω*_22_. We then proceeded by alternating between the expectation and maximization steps. After each maximization step, we applied the following additional constraints:

1. Mode identity: to guarantee that each mode was associated with the same category across groups, we required that ${\|{\mu }_{i}-{\mu }_{i}^{0}\|}_{2} < {\frac{1}{2}d}_{\min }$, where ${\mu }_{i}^{0}$ is the *i*th category in *Ω*_22_ and where *d*_min_ is the minimal distance between categories in *Ω*_22_. This constraint permits the modes to deviate substantially from integer ratios to faithfully represent bias in the data, but not so much that the correspondence with integer ratios is lost.
2. Overlap: to avoid overlap between the modes, we required that the eigenvalues of the covariance matrix *λ*_1_ and *λ*_2_ satisfy the constraint that $\left|{{\lambda }}_{i}\right| < {d}_{\min }$.
3. Additional constraints on the overlap between the modes: we also required that *λ*_1_ and *λ*_2_ be limited by $A < \frac{{\lambda }_{i}}{{\lambda }_{1}+{\lambda }_{2}} < 1-A$, where *A* is a constant. We fixed *A* = 1/5, which intuitively corresponds to a constraint on the aspect ratio of the ellipsoid defined by the covariance matrix.

We applied these constraints after the maximization step. We applied constraint 1 by projecting the *μ*_*i*_ resulting from maximization step to the closest point in Euclidean distance that satisfied constraint 1. Similarly, we applied constraints 2 and 3 on the eigenvalues of the covariance matrix by truncating them so they satisfied both constraints:

$${\lambda' }_{i}=\min\left({\lambda }_{i},{\frac{1}{2}d}_{\min }\right)$$

$${\lambda^{\prime\prime}}_{i}=\min{\left(\max\left({\lambda^{\prime}}_{i},A({\lambda^{\prime}}_{1}+{\lambda^{\prime}}_{2})\right),(1-A)({\lambda^{\prime}}_{1}+{\lambda^{\prime}}_{2})\right)}$$

We then iterated these steps until convergence (using a convergence threshold of 1 × 10^−6^). The result was an estimate of {*μ*_*i*_}_*i*=1…*K*_, {*C*_*i*_}_*i=*1*…K*_ and {*w*_*i*_}_*i=*1*…K*_. We emphasize that these constraints do not place strong limits on the locations of the modes, which are free to exhibit biases, or on the category boundaries, which need not be located symmetrically between modes. The constraints merely serve to enable us to label the modes in a consistent way. A Gaussian mixture model fit in this way to each group’s data explained most of the kernel density variability (91% on average, ranging from 80.6% to 97.5% depending on the group).

This procedure is different from the one reported in Jacoby and McDermott^26^, where we performed a numeric constraint optimization with the MATLAB fmincon function on the Kullback–Leibler divergence of the kernel density estimate defined by the model and the kernel density estimate of the data. Other than this difference, we had similar constraints on the optimization. The 2017 procedure provided comparable results but was slower than the method used here. Considering the large amount of data in this project, we considered the efficient expectation–maximization method to be preferable to direct optimization.

#### Gaussian mixture model with 7:2:3 category

In the case of Malian drummers and dancers, we added additional rhythm categories at 2:3:7, 7:2:3 and 3:7:2 (Figs. 5 and 7g–i). We denote this by *Ω*_25_ = *Ω*_22_∪{2:3:7,7:2:3,3:7:2} and used it instead of *Ω*_22_ for the Gaussian mixture model estimate. In all other respects, the analysis was identical to that in the previous section (Gaussian mixture model fit with *Ω*_22_).

#### Average category weights

In all cases, we fitted the Gaussian mixture to the categories (*Ω*_22_, except for Fig. 7g–i, where we used *Ω*_25_). In some cases, we wanted to display or analyse the results ignoring cyclic permutations of the same category (for example, 2:2:3, 2:3:2 and 3:2:2 would be mapped to the same category 223). We then computed the Gaussian mixture model fit to *Ω*_22_ and averaged the weight across the three permutations. The one exception was the isochronous rhythm 1:1:1, which has no variants; in this case we used the original fit of 1:1:1 in *Ω*_22_. This resulted in eight weights per group corresponding to the eight categories in *Ω*_8_ (defined above in ‘Small-integer ratios’). These category weights are reported in Extended Data Fig. 4 and are provided as part of the OSF repository associated with this publication.

#### Significant distance between two groups

In this analysis, we evaluated whether two distributions *P*_1_ and *P*_2_ associated with two groups had significantly different kernel density estimates. Since the Jensen–Shannon divergence is always positive, it deviates from zero when the kernel density estimates being compared are computed from a finite sample. We used bootstrapping to estimate whether the distance between *P*_1_ and *P*_2_ was greater than what is expected from this finite-sampling effect. We created 1,000 simulated split halves of the trials from each participant group. From these bootstrap samples, we estimated the 1,000 kernel density estimates associated with the two splits of each group (we denote them by ${P}_{1}^{\;j,1}$ and ${P}_{1}^{\;j,2}$, where *j* indexes the 1,000 split halves, and ${P}_{2}^{\;j,1}$ and ${P}_{2}^{\;j,2}$). We then computed ${\rm{JSD}}\left({P}_{1}^{\;j,1},{P}_{2}^{\;j,1}\right)$ (the distance between split halves across groups) and compared it with ${\rm{JSD}}\left({P}_{1}^{\;j,1},{P}_{1}^{\;j,2}\right)$ and ${\rm{JSD}}\left({P}_{2}^{\;j,1},{P}_{2}^{\;j,2}\right)\,$. We assessed statistical significance via a *P* value from the minimum of the rank order of ${\mathrm{JSD}}\left({P}_{1}^{\;j,1},{P}_{2}^{\;j,1}\right)$ within the two null distributions for ${\mathrm{JSD}}\left({P}_{1}^{\;j,1},{P}_{1}^{\;j,1}\right)$ and ${\mathrm{JSD}}\left({P}_{2}^{\;j,1},{P}_{2}^{\;j,1}\right)\,$. Namely, to declare that two groups are significantly different, their mean Jensen–Shannon divergence had to be significant with respect to both within-group Jensen–Shannon divergences. We also computed the difference in Jensen–Shannon divergence:

$$\begin{array}{l}D=\left[\left({\rm{JSD}}\left({P}_{1}^{\;j,1},{P}_{2}^{\;j,1}\right)-{\rm{JSD}}\left({P}_{1}^{\;j,1},{P}_{1}^{\;j,1}\right)\right)+\left({\rm{JSD}}\left({P}_{1}^{\;j,1},{P}_{2}^{\;j,1}\right)\right.\right.\\\qquad\left.\left.-\,{\rm{JSD}}\left({P}_{2}^{\;j,1},{P}_{2}^{\;j,1}\right)\right)\right]/2.\end{array}$$

We report the mean of the difference (mean(*D*)) as well as the 95% CIs of *D*.

#### Discrete mode (‘peakiness’) analysis

We performed three analyses to substantiate the presence of discrete modes in the measured priors. In analysis 1, to show that the mass of the estimated density was centred in a small part of the space, we computed for each group the 33% of the bins with the highest kernel density and then computed the sum of the density in these bins relative to the sum over all bins. This resulted in numbers ranging between 61.8% and 81.7% (mean 70.1%) over the 39 groups. To obtain a null distribution for this quantity, for each group we sampled points (equal in number to the total number of trials for that participant group) randomly on the triangle and estimated the empirical kernel density estimate for this random distribution. We then repeated the selection process described above, picking the 33% of the bins with the highest kernel density and computing the proportion of the summed density in these bins. This analysis showed that the percentage obtained in this way from the empirical data was significantly larger than would be expected from the null distribution computed from uniform sampling.

In analysis 2, we estimated the peak density with respect to a uniform distribution. We identified the bin in the kernel density estimate with the highest density and found that in all 39 groups this bin had a density that was over five times larger than the density of the same bin under a uniform distribution (range, 5.3–13.1; mean, 8.8). We estimated the statistical significance of this ratio using a null distribution obtained by sampling points from a uniform distribution (using the same number of points per group) and measuring the peak density from the resulting kernel density estimate. We found that the empirical peak ratios were significantly larger than would be expected by chance for all 39 groups (*P* < 0.001 in each case).

In analysis 3, we fit a Gaussian mixture model with mixture components constrained to be near small-integer ratios (see ‘Gaussian mixture model fits’ for the details). This model explained most of the variance in the kernel density (91% on average, ranging from 80.6% to 97.5% depending on the group). Explained variance was measured here by treating the kernel density estimates of both the empirical data and the models as vectors and squaring the correlation between the vectors.

#### Overlap with small-integer ratios

We evaluated overlap with small-integer ratios using three different analyses (Fig. 4a). First, we computed the average minimal distance between each fifth-iteration reproduction (for all participants in a given group) and the closest small-integer-ratio rhythm: *L*_2_(*Ω*_22_, *p*_*i*_), where *p*_*i*_ are the fifth-iteration reproductions represented on the rhythm triangle, *Ω*_22_ is the set of small-integer ratios involving the numbers 1–3 defined above and *L*_2_ is the minimum of the Euclidean distances between the point *p*_*i*_ and each of the 22 points in *Ω*_22_. To show that these distances are significantly smaller than would be obtained by chance, we generated a null distribution by randomly sampling sets of 22 points uniformly from the triangle and computing the same mean minimal distance between the points *p*_*i*_ and these randomized sets. We then compared the empirical distance to the null distribution. We used this first analysis for the results reported in Fig. 4a on the grounds that it is simple to describe.

Second, we performed an additional control analysis where instead of sampling the sets of 22 points uniformly, we constrained them so that each point fell within a circle of radius *d* around the integer points, where *d* is 1/2 of the minimal distance between two points in *Ω*_22_. This guaranteed that the null sets were spaced similarly to *Ω*_22_. The results of this alternative analysis were similar to those of the simpler analysis described above, and in all 39 cases the empirical distance was significantly smaller (*P* < 0.001) than would be expected from the null distribution, even when Bonferroni correction was applied.

Third, we applied the integerness score reported in Jacoby and McDermott^26^. In this analysis, we compared the Jensen–Shannon divergence distance between the empirical kernel density estimate of the fifth iteration (*P*) and the normalized indicating function ${I}_{{\varOmega }_{22}}(x)$ $=\frac{1}{22}\sum _{\omega \in {\varOmega }_{22}}\delta \left(x-\omega \right)$, where *δ* is the Dirac delta function on the triangle. This Jensen–Shannon divergence is maximal if all probability mass is located at the small-integer ratios. We initially fitted an unconstrained Gaussian mixture model with 22 components. We then randomized the means of the components of this mixture. This simulates a response distribution that is similar in statistical characteristics to the data, but that is not centred around integer ratios. We obtained a null distribution by generating 1,000 such randomized distributions, each time computing the Jensen–Shannon divergence with ${I}_{{\varOmega }_{22}}(x)$. We then compared the distance of ${I}_{{\varOmega }_{22}}(x)$ and *P* to this null distribution. We found that in all cases the distance between ${I}_{{\varOmega }_{22}}(x)$ and *P* was significantly smaller (*P* < 0.01) than would be expected by chance.

#### Bias analysis

Figure 4b displays an analysis testing whether perceptual category centres are systematically biased away from the corresponding small-integer ratio. The small grey dots plot the component means of the fitted Gaussian mixture models for each category and participant group. We then calculated the empirical means (indicated with larger black dots) of each category across all groups. We applied a non-parametric test analogous to an analysis of variance to test whether each category was biased. The test statistic was the ratio of (1) the average squared Mahalanobis distance between all points and the empirical mean and (2) the average squared Mahalanobis distance between all points and the corresponding small-integer ratio. We compared this test statistic to its null distribution computed from 10,000 bootstrapped samples where the data were randomly sampled from a Gaussian distribution with the same empirical covariance matrix as the experimental data but with the mean set to the integer ratio category (that is, with zero bias).

Nine categories showed small but significant deviations from unbiased integer ratio categories after Bonferroni correction (*P* < 0.0012 for all cases; see the blue significance symbols in Fig. 4b: ***P < 0.001; **P < 0.01). The biased categories consisted of the three cyclic permutations of each of 1:2:3, 2:1:3 and 2:2:3 (123/231/312, 213/321/132 and 223/232/322). The bias of the ‘6/8’ categories 1:2:3 and 2:1:3 is consistent with lengthening of short elements in rhythm performance studied in European musicians^42^. It is also evident in the experimental literature on rhythm perception on Western European and North American listeners. Fraisse^121^ showed that non-musicians have a small bias when judging three-interval rhythms, tending to judge the two shorter intervals as being closer to equal. Repp et al.^122^ argue that this bias originates from categories near 1:2:3 and 2:1:3 that are slightly shifted away from those integer-ratio rhythms, in a direction that lengthens the shortest interval (a phenomenon we also observed, cross-culturally). The results are also consistent with the observation that the shortest interval of a two-interval rhythm is heard as elongated, making the rhythm more similar to isochrony^123^. It may also be related to the phenomenon of non-isochronous beat subdivision in African and African-American music genres (for example, jembe music from Mali and ‘swing’ jazz from the United States), in which the short interval in short-long rhythms is often markedly elongated relative to a 1:2 ratio^124^.

#### Multidimensional scaling analysis

To visualize the similarity relations between the rhythm priors for each participant group, we first estimated the priors as the kernel density estimate *P*_*i*_ from the fifth iteration of the experiment (aggregated for all participants in each group; see above; Fig. 5a). We then computed the Jensen–Shannon divergence between all pairs of groups *M*_*ij*_ = JSD (*P*_*i*_, *P*_*j*_). We used MATLAB’s mdscale function with the default parameters to obtain a two-dimensional space in which the rhythm prior for each group was positioned so as to best match the measured distances. Note that we used the distances between the full distributions (that is, the kernel density estimates) for the multidimensional scaling analysis (as opposed to the Gaussian mixture models used in other analyses).

#### Category weight for the 3:3:2 rhythm

To compute the weight of the 3:3:2 rhythm for each group, we computed the Gaussian mixture model weights as explained above for the 22 rhythm categories and then averaged the weights over the three cyclic permutations of 3:3:2 (3:3:2, 3:2:3 and 2:3:3; Fig. 5b). We obtained error bars via bootstrapping, sampling 1,000 datasets with replacement for each group and computing the weights of the Gaussian mixture model for each of these datasets. The error bars plot one standard deviation of the resulting distribution above and below the mean (that is, the standard error of the mean). The order of the groups in the bar graph of Fig. 5b is drawn from the first dimension of the multidimensional scaling analysis, and it is obvious that this dimension is correlated with the 3:3:2 category weight (which increases nearly monotonically across the first multidimensional scaling dimension).

#### Analysis of student and online groups

In the first analysis, we computed the average distance (Jensen–Shannon divergence) between the estimated priors of all pairs of student or online groups in different countries and compared it to that of pairs of non-musician and local musician groups (from the same countries as the student/online groups; Fig. 6a,d). The pairs we considered were within the following sets of groups:

- Figure 6a: for students, US (Boston)-ST, Bolivia (La Paz)-ST, Uruguay-ST, UK-ST, Turkey-ST, Mali-ST, S. Korea-ST and Japan-ST; for non-online groups, US(NYC)-NM, US(NYC)-LM, Bolivia (La Paz)-NM, Bolivia (San Borja)-NM, Bolivia (Santa Cruz)-NM, Bolivia (Tsimane)-NM, Uruguay-LM, UK-LM, Turkey-LM, Mali-LM, S. Korea-LM and Japan-LM.
- Figure 6d: for online groups, US-OL, Brazil-OL and India-OL; for non-students, US (NYC)-NM, US (NYC)-LM, Brazil-LM, India-NM and India-LM.

To evaluate the statistical significance of the difference in distances, we created shuffled datasets where two sets of groups (one the same size as the student/online set and one the same size as the non-student/non-online set) were sampled without replacement from the union of the student/online and non-student/non-online sets. We then computed the difference between the average Jensen–Shannon divergences of these shuffled groups for each resampling and evaluated the probability of the actual difference under this null distribution.

In the second analysis, we computed the average distance (Jensen–Shannon divergence) between the US student group (US(Boston)-ST) and the priors of all other student/online groups (student: Bolivia(La Paz)-ST, Uruguay-ST, UK-ST, Turkey-ST, Mali-ST, S.Korea-ST and Japan-ST; online: US-OL, Brazil-OL and India-OL). We compared this average distance to a null distribution obtained by sampling sets of non-student/non-online groups of the same size (student: seven groups; online: three groups) and measuring the average distance of each set of groups and the US student group.

To control for the fact that student groups tended to be younger than other groups, we repeated the above two analyses restricted to participants younger than 40. The group differences in mean age were not eliminated by this restriction, but they were significantly reduced (with this restriction, all groups had mean ages between 21 and 33.7 years and could all be considered ‘young’). The statistics reported in ‘Students and online participants resemble US participants’ in ‘Results’ use pairwise bootstrapped Jensen–Shannon divergence (see ‘Significant distance between two groups’).

We also performed a control analysis testing for effects of age by comparing the differences in the kernel densities between young and old subsets of the online groups. Given that over a third—specifically, 46%, 34% and 39% in the US, Indian and Brazilian groups, respectively— of participants in the online groups were older than 35, we divided all groups into younger and older subsets using the age of 35 as a threshold. We found that kernel density estimates for the US, Indian and Brazilian groups were not significantly different (see ‘Students and online participants resemble US participants’ in ‘Results’). These results suggest that age does not explain the increased similarity of student populations.

#### Word clouds of favourite music

In the demographic questionnaire, we asked participants to list their three favourite bands or musical artists and to indicate the genre for each (Fig. 6b,c,e,f). The level of detail varied somewhat between individuals and groups (for example, some individuals specified sub-genres such as ‘indie-rock’, whereas others indicated ‘rock’). Due to site-specific limitations on the experiment session duration, this question was asked of only 31 of the 39 groups. Text entries were verified by searching each entry in the Google Knowledge Graph Search API (https://developers.google.com/knowledge-graph). In case of items with incomplete matches, spelling errors were manually corrected. We then analysed the results using single-word histograms. Some of these histograms are presented as word clouds in Fig. 6, with the font size proportional to the frequency of occurrence. In addition, the most common words for each group are presented in Supplementary Table 2. This analysis is qualitative but nonetheless provides concrete evidence for the differences in musical listening habits between student/online and non-student/non-online groups.

#### Analysis of specific modes

Figure 7 analyses the prominence of particular rhythm modes in different participant groups. For each group, we computed the Gaussian mixture model weights as explained above for the 22 rhythm categories and then averaged the weights over the three cyclic permutations of the rhythm in question. We then examined these weights for participant groups whose local musical tradition was known to feature the rhythm. We asked whether the weights were higher than in the remaining participant groups using a Wilcoxon rank-sum test.

The 2:2:3 and 3:3:2 rhythms had been previously associated with specific musical traditions. 2:2:3 has been documented in Balkan, Turkish and Botswanan music^49–51^, which often employ metres with a signature of 7/8. Balkan and Turkish listeners have also been shown to better discriminate this pattern than US and Canadian participants without such familiarity^53,125^. The 3:3:2 rhythm is similarly ubiquitous across sub-Saharan Africa^54,55^ and the African diasporas^56–59^ in the Americas. We confirmed its presence in the musical culture of our Malian dancer participants (Mali-DA, recruited among farmers from Sagele village in Southern Mali) by recording and analysing a representative corpus of their musical repertoire. The pieces chosen were ones to which they frequently danced in the context of wedding celebrations and other local events. We found that 46% of the recorded excerpts prominently featured a 3:3:2 pattern, making it one of the most characteristic rhythmic patterns in this repertoire.

The 7:2:3 rhythm evident in the priors measured from drummers in Mali (Fig. 7g–i) is popular in West Africa; a slightly denser, five-interval variant (2:2:3:2:3) constitutes a signature rhythm that is emblematic of the musical culture area^54,55^. Drumming in Mali is multi-part ensemble music composed of three basic parts: an improvising lead drum, a simple invariant accompaniment and a ‘timeline’ part, whose specific rhythm patterns identify each piece of repertoire^126^. The ‘Maraka’ is the most frequently performed piece in their repertoire^127^. One characteristic timeline pattern for the Maraka consists of three accented events that are distributed according to a 7:2:3 pattern across a periodicity composed of 12 metric units (7 + 2 + 3). This pattern is often performed by the timeline player, who alternates during the piece between this pattern and other variants with similar accents. Additionally, we substantiated its presence in Malian music as described in ‘Results’. We used the same procedure to validate participant responses in Bulgaria (2:2:3 rhythm).

#### Violin plots

To generate violin plots (used in Figs. 7 and 8 and Extended Data Fig. 8), we used Bastian Bechtold’s Violin Plots for MATLAB package (https://github.com/bastibe/Violinplot-Matlab, 10.5281/zenodo.4559847). The open circle plots the median, and the top and bottom of the grey bar plot the 75th and 25th percentiles. The violin plots are kernel density estimates of the data distribution. Whiskers (thin lines) are computed using Tukey’s method^128^ and reflect the range of non-outlier points.

#### Tapping precision and asynchrony in musicians and non-musicians

To compare objective precision in our task between musicians and students/non-musicians, we used Wilcoxon tests (one-sided), again Bonferroni-corrected (Fig. 8). For the musician groups, we included both those playing Western music and those playing local musical styles. The measure used to assess tapping precision was the standard deviation of the tapping asynchrony (the time difference between a stimulus click and the corresponding tapped response)^3^, computed over all valid tapped responses in the main experiment. We also compared the mean of the tapping asynchrony, again computed over all valid tapped responses in the main experiment. The negative mean asynchrony reflects the tendency of taps to occur before the stimulus (in anticipation of the upcoming stimulus).

#### Cross sections of priors

To see the structure of the modes of the priors, we used an alternative visualization. Extended Data Fig. 1 displays 1D plots of estimated priors from four groups: three that show elongated modes (BO.TS, IN.OL and UY-ST) and, as a comparison, one group with more symmetric modes (UY-LM). We also show 2D and 3D plots of the priors for comparison. The 3D plots were generated with MATLAB’s surf function.

#### Cyclic permutations and an analysis of symmetry

Across the groups we tested, the response distributions were typically fairly symmetric across cyclic permutations (Fig. 3 and Extended Data Fig. 3). For example, the modes at 1:1:2, 1:2:1 and 2:1:1 have about the same weight for a given participant group. To quantify this symmetry, we compared the percentage of responses in the final iterations that are in each of the three possible cyclic permutations, which can be identified by whether the longest interval is in the first, second or third position, defined relative to the beginning of the stimulus (Extended Data Fig. 3a).

As is evident in Extended Data Fig. 3b, the deviations from perfect symmetry were relatively modest (perfect symmetry would yield 33.3% of tapped responses in each third of the space; actual proportions ranged from 24.3% to 43.6%; the standard deviation of the difference from 33.3% was 3.2%). However, these deviations appeared to be non-random, with a tendency for more weight on permutations in which the third interval is the longest (red region). Previous literature^43–45^ in fact predicts that the most frequently occurring permutations would be those where the long interval occurs at the end, because if this configuration is played cyclically, the long interval provides a gap that helps the pattern group according to Gestalt principles^43^. This was the case in 31 of 39 groups (those for which the green area in Extended Data Fig. 3b extends beyond the horizontal line; this number is much greater than would be expected by chance, *P* < 0.001 via a binomial test; the mean percentage of long-interval-at-the-end patterns was 36%; Cohen’s *d* = 0.73; 95% CI, (34.7%, 36.5%)). We also found that in 33 of the 39 groups (again much greater than expected by chance, *P* < 0.001 via a binomial test), the majority of the first taps within a block occur right after the long interval (the mean percentage across groups of tapping after the long interval was 47%; 95% CI, (43.6%, 50.7%); Cohen’s *d* = 1.2). This suggests that most participants in most groups tend to perceive the onset after the long interval as the ‘beginning’ of the pattern and align their first response to it.

What can explain the overall tendency towards symmetry? In principle, the symmetry could reflect the fact that the beginning of a repeating cycle is ambiguous if participants ignore or forget the initial interval. In an earlier paper^26^, we tested whether this ambiguity underlies the symmetry evident in the response distributions. Specifically, we performed an experiment in which the first click of the repeating stimulus was given a 7 dB level increment to render the permutations distinct. The results (Supplementary Fig. 5 in that paper) indicated that the symmetry of the response distribution was largely maintained, suggesting that perceptual ambiguity is not the only reason for symmetry in the prior.

Another possibility is that the perception of simple periodic rhythms is influenced by grouping multi-stability, whereby the same stimulus rhythm can be perceived in different sequential arrangements given that starting points are subjective and may be interchangeable to some degree^129,130^. For instance, a listener might hear the first element of a stimulus as an ‘upbeat’ (anacrusis) preparing the second element to represent the perceptual beginning^67,95^. Under this interpretation, cyclic permutations of an interval pattern have different beginnings but would otherwise be perceptually similar.

#### Category weights

Categorization weights were computed for each group for the seven category types (Extended Data Fig. 4). As part of the OSF repository, we have provided the raw data for these category estimates.

#### Multidimensional scaling and category weights

We computed the correlation between projections of the priors onto the multidimensional scaling dimensions and each of the category weights for each group, averaged across cyclic permutations (Extended Data Fig. 5). The multidimensional scaling projections and Gaussian mixture model weights were computed as described above. The correlation was computed across the 39 groups. The CIs were obtained using the RIN method^131^.

#### Principal component analysis

In Fig. 3, we used multidimensional scaling to perform dimensionality reduction, computing the Jensen–Shannon divergences between the kernel density estimates of all pairs of groups. As an alternative, we performed an analogous analysis using principal component analysis (Extended Data Fig. 6). We treated the triangle images generated from the kernel density estimates as large feature vectors, where each pixel is a feature (using kernels with a resolution of 12 ms—that is, 0.006× the pattern duration of 2,000 ms). We computed the principal components of these vectors across 39 groups. The projections of the 39 groups’ priors onto the first two principal components showed a structure very similar to what we obtained with multidimensional scaling (Extended Data Fig. 6a). For example, it is apparent that student groups were again centred in the middle. As with multidimensional scaling, the projection of each group’s estimated prior onto the first principal component was positively correlated with the 2:3:3 category (*r*_37_ = 0.94; *P* < 0.0001; 95% CI, (0.89, 0.97)) and negatively correlated with the simpler categories (1:1:1: *r*_37_ = −0.44; *P* = 0.04; 95% CI, (−0.66, −0.14); 1:1:2: *r*_37_ = −0.59; *P* < 0.001; 95% CI, (−0.76, −0.33), 1:2:2: *r*_37_ = −0.70; *P* < 0.001; 95% CI, (−0.83, −0.49); Extended Data Fig. 6c). The projection onto the second principal component was correlated with 6/8 rhythms (1:3:2: *r*_37_ = 0.66; *P* < 0.001; 95% CI, (0.44, 0.81); 1:2:3: *r*_37_ = 0.74; *P* < 0.001; 95% CI, (0.56, 0.86)) as well as the 1:1:2 rhythm (*r*_37_ = −0.63; *P* < 0.001; 95% CI, (−0.79, −0.4)). The CIs were obtained using the RIN method^131^. The components can also be visualized (Extended Data Fig. 6b), revealing that their minima and maxima overlap with small-integer-ratio categories. The consistency between the different dimensionality reduction methods indicates the robustness of the results. The raw data for the category fitting and category weight for each group are also provided in the OSF repository associated with this publication.

#### Category predictions from rhythm priors

In this analysis (Extended Data Fig. 7), we used human psychophysical data previously obtained and published by Desain and Honing^9^ (the data were available on a website: https://www.mcg.uva.nl/index.html). In their experiment, 29 Western musicians heard one of 66 rhythms (an equally spaced array of points on the rhythm triangle) and used notation software to specify the rhythm that they heard. Because this experiment used Western musical notation, it was possible only in Western musicians. The 29 participants were highly trained professional musicians and advanced conservatory students from Dutch conservatories and from the Kyoto City University of the Arts in Japan. They had received between 7 and 17 years of musical training and were paid for their participation.

When the data were pooled across participants, there were 133 different responses in total. Each response can be expressed in ratio form. For instance, the most common response was 1:1:1, and the second most common response was 1:2:1. The results of the experiment were summarized as a set of regions associated with each musically notated rhythm as the most frequent category choice (Extended Data Fig. 7a, which was our reproduction of Fig. 11 from the Desain and Honing paper using the data we downloaded). To obtain this figure, we followed the two steps below:

1. For each of the 133 responses, we created a kernel density plot representing the interpolated probability of this response at each point on the rhythm triangle. We used a kernel width of 0.03.
2. We found the response with the largest interpolated weight at each point on the triangle.

The resulting figure contains 17 distinct rhythm categories spread over the rhythm triangle.

We generated analogous regions for the model’s categorization judgements using each group’s prior (Extended Data Fig. 7b). We used the Gaussian mixture model that we previously fitted to the tapping data (see ‘Gaussian mixture model fits’). This model is defined by three parameters: category centres {*μ*_*i*_}_*i*=1…22_, covariance matrices {*C*_*i*_}_*i*=1…22_ and weights {*w*_*i*_}_*i*=1…22_, which approximate the priors from the tapping data:

$$Q(x)=\mathop{\sum }\limits_{i=1}^{22}\frac{{w}_{i}}{2\pi \sqrt{{\rm{|}}{C}_{k}{\rm{|}}}}\exp \left(-\frac{1}{2}{\left(x-{\mu }_{i}\right)}^{{\mathrm{T}}}{C}_{k}^{-1}(x-{\mu }_{i})\right)\,$$

The model selected the category whose corresponding mixture component had the highest value at each point in the triangle. But on the basis of empirical findings that human categorical judgements are best predicted by a nonlinear transform of the underlying probability distribution^132^^,133^, we used mixture weights that depended exponentially on the prior weights:

$${U}_{i}(x)=\frac{{w}_{i}^{\gamma }}{2\pi \sqrt{{\rm{|}}{C}_{k}{\rm{|}}}}\exp \left(-\frac{1}{2}{\left(x-{\mu }_{i}\right)}^{{\mathrm{T}}}{C}_{k}^{-1}(x-{\mu }_{i})\right)\,$$

where *γ* > 1 is a parameter that prioritizes high-probability categories. We selected the value of *γ* as that which maximized the match between the human category judgements and those predicted by the prior estimated from the New York Western musician group (US.NY-WM), yielding *γ* = 7. We then omitted the US.NY-WM group from the subsequent analysis to avoid non-independence. Additionally, we found empirically that the category 1:1:1 was overrepresented in the human categorization judgements relative to those of the model. We note that this category is unique in that all three cyclic permutations correspond to the same point on the rhythm triangle, which might cause participants to choose it more than other categories. To accommodate this effect, we increased the weight *w*_*i*_ on the 1:1:1 category by a factor of three. However, the cross-cultural differences shown in Extended Data Fig. 7 were not dependent on this choice (we observed significant differences in the match of Western groups compared with the two non-Western groups in both cases—the matches were just overall worse without the overweighting).

We quantitatively assessed the match between the categories predicted by a group’s prior and those measured in Western musicians as the average distance between the two predicted categories. Specifically, for every sampled point on the rhythm triangle, we compared the model prediction with the top category selected by participants in the Desain and Honing experiment, and we measured the *L*_2_ distance in the three-dimensional space of the three intervals in the rhythm, expressed as ratios (proportions of the total pattern duration). For instance, if the participants selected the category [0.5, 0.25, 0.25] and the model predicted [0.5, 0.2, 0.3], then the distance was ||(0, −0.05, 0.05)||. We then averaged this distance for each rhythm in the experiment (that is, every sampled point on the rhythm triangle) to yield an overall measure of the match between the model’s predictions and Western musician category judgements (plotted in Extended Data Fig. 7c). To compare the accuracy of this match across sets of groups, we used a Wilcoxon rank-sum test. We used three sets of groups, defined as follows: Western participants (US.BO-ST, US.BO-WM, US.NY-NM, US.NY-WM, BO.LP-ST, UY-ST, UK-ST, TK-ST, MA-ST, KR-ST, KR-WM, JP-ST, JP-WM and US-OL), non-Western non-musicians (BO.LP-NM, BO.SB-NM, BO.SC-NM, BO.TS-NM, NA-NM, IN-NM, CN-NM, BR-OL and IN-OL) and non-Western musician and dancers (BR-LM, UY-LM, SE-LM, BG-LM, BG-DA, TK-LM, MA-LM, MA-DA, BW.SA-LM, BW.EA-LM, NA-LM, IN-LM, KR-LM and JP-LM). We excluded jazz musicians in the United States and the United Kingdom as there was not a natural hypothesis regarding how their priors would predict the categories of Western classical musicians.

#### Validation of musicianship

To compare self-reported years of musical experience between musician and non-musician groups (Extended Data Fig. 8), we used Wilcoxon tests (one-sided), applying Bonferroni correction for multiple comparisons.

#### Influence of language and musicianship

In this analysis, we evaluated whether two groups that spoke the same or different languages (or that differed in musicianship) had significantly different kernel density estimates (Extended Data Fig. 9). We used the procedure described in ‘Significant distance between two groups’ (bootstrapped Jensen–Shannon divergence).

#### Transmission error

Transmission error is the magnitude of the difference between the stimulus and response seeds in each iteration. It is used in the serial reproduction literature to monitor convergence dynamics^134^^,135^. As an error measure, we computed the average across trials of $e=\sqrt{{{(s}_{1}-{r}_{1})}^{2}+{{(s}_{2}-{r}_{2})}^{2}+{{(s}_{3}-{r}_{3})}^{2}}$, where (*s*_1_, *s*_2_, *s*_3_) and (*r*_1_, *r*_2_, *r*_3_) are the stimulus intervals and average response intervals of each iteration, respectively (that is, the response is averaged across the ten repetitions within each iteration). In our previous work, we showed that convergence occurred after about five iterations for both Tsimane’ and US participants. Here we show similar dynamics for all groups (Supplementary Fig. 2).

#### High-resolution prior visualizations

In Supplementary Figs. 3–9, we provide higher-resolution images for the measured priors presented in Fig. 3b and Extended Data Fig. 2.

### Fast-tempo experiment

#### Procedure

When experimental conditions allowed for longer sessions, we ran an additional experiment to explore whether the results would be similar at other tempos. The experiment was always run last, and was identical to the main experiment except that the pattern duration was 1,000 ms. The other experimental constants (for example, the fastest allowed interval) were scaled accordingly (the experiment was identical to the fast-tempo experiment in Jacoby and McDermott^26^, experiment S2, shown in Supplementary Fig. 3 of that paper).

#### Participants

A total of 293 participants from 13 groups (6 countries) participated in the fast-tempo experiment. These participants performed 7,587 trials (seeds) with 911,564 taps. The demographic information for these participants is summarized in Supplementary Table 1.

#### Analysis of results

The kernel density estimates of the 13 groups are provided in Extended Data Fig. 2. We provide the raw data of the experiment in the OSF repository associated with this publication.

Overall, the results at the faster tempo were similar to those at the slower tempo. All 13 groups who performed the fast-tempo experiment produced priors that were closer to integer ratios than would be obtained by chance (*P* < 0.001 in all cases), even with Bonferroni correction.

Supplementary Fig. 1 shows an analysis of the weights of the modes in the 13 groups. The weights of the 22 categories were correlated across the two tempos (*r* = 0.35–0.72 for each of the 13 groups; *P* = 0.0001–0.05; mean *r* = 0.57; s.d. = 0.1). As expected from previous literature, there were also some subtle differences between the category weights for the two tempos (Supplementary Fig. 1). Three of the four largest effect sizes were found in dancers (Bulgarian dancers: effect size of 5.5—more weight on category 2:2:3 in the fast tempo; Malian dancers: effect size of 5.6—more weight on 1:2:3 in the fast tempo; Malian dancers: effect size of 4.9—decreased weight on 3:3:2 in the fast tempo). These tempo-dependent effects in dancers are consistent with the idea that dancers have an increased sensitivity to tempo and to embodied aspects of music^136,137^. For instance, Bulgarian dancers showed much more weight on 2:2:3 at the faster tempo. Bulgarian folklorists have long recognized tempo as an important factor in metrical patterns that feature a 2:2:3 ratio, such that the metric durations are considered fundamentally unequal only when performed at fast tempos^138,139^. This idea was also reflected in one of the interviews we conducted with the musicians after the experiment. When we asked one participant whether she recognized the 2:2:3 pattern with a period of 2,000 ms, she identified it as the rhythm of a Bulgarian dance type called *rŭchenitsa*, but slower than usual. The effect of tempo in Malian dancers’ 1:2:3 category is similarly consistent with the findings of Polak et al.^27^, who showed that reproductions of short–long patterns in two-interval rhythms (the first part of the 1:2:3 pattern) strongly vary with tempo. This pattern is characteristic of the three most common Malian jembe musical pieces: Maraka, Suku and Manjanin, which are typically performed at a very fast tempo (100–200 beats per minute)^126^.

As the tempo increases, one might expect to see effects related to whether the temporal intervals in a rhythm are readily producible by humans^93^. We did not see clear evidence for this at the 1,000 ms tempo (specifically, the rhythms with the shortest intervals—123 and 132—did not have significantly lower category weights for 1,000 ms than for 2,000 ms, as evaluated with a binomial test), though there were some trends in this direction. It seems likely that for sufficiently fast tempos, and with enough data, such effects would be detectable.

### Reporting summary

Further information on research design is available in the Nature Portfolio Reporting Summary linked to this article.

### Supplementary information

Supplementary Information Supplementary Tables 1–3 and Figs. 1–9.

Reporting Summary

## Figures and Tables

**Extended Data Table 1:**
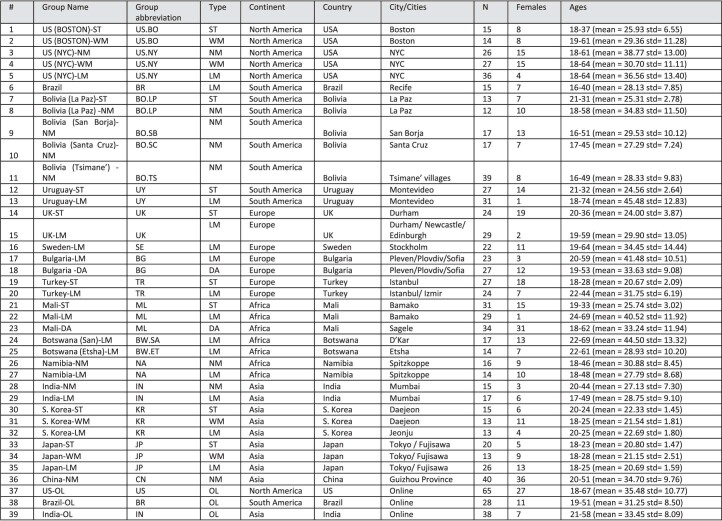
Demographic information

Participant Group Types: ST = student; NM = non-musician; WM = Musicians who play Western classical music; LM = musicians who play a local musical style that is not Western classical music; DA = dancer; OL = Online participants. Additional demographic information is provided in Supplementary Table 2.

## Data Availability

The raw data for all groups, the instructions for running the paradigm and the data plotted in each figure are provided in the OSF repository associated with the paper: https://osf.io/6zd4v/ (ref. ^140^).

## Code availability

The code for running the experiment and for performing all analyses, including those to generate all results graphs, is provided in the OSF repository associated with the paper: https://osf.io/6zd4v/ (ref. ^140^).

## References

[1] Patel, A. D. *Music, Language, and the Brain* (Oxford Univ. Press, 2008).

[2] Sundberg, J., Prame, E. & Iwarsson, J. Replicability and accuracy of pitch patterns in professional singers. in *Vocal Fold Physiology, Controlling Complexity and Chaos* (eds Davis, P. J. & Fletcher, N. H.) 291–306 (Singular, 1996).

[3] Repp BH (2005). Sensorimotor synchronization: a review of the tapping literature. Psychon. Bull. Rev..

[4] Vurma A, Ross J (2006). Production and perception of musical intervals. Music Percept..

[5] Larrouy-Maestri P, Morsomme D (2014). Criteria and tools for objectively analysing the vocal accuracy of a popular song. Logoped. Phoniatr. Vocol..

[6] Roeske TC, Tchernichovski O, Poeppel D, Jacoby N (2020). Categorical rhythms are shared between songbirds and humans. Curr. Biol..

[7] Siegel JA, Siegel W (1977). Categorical perception of tonal intervals: musicians can’t tell sharp from flat. Percept. Psychophys..

[8] Clarke, E. F. in *Action and Perception in Rhythm and Music* (ed. Gabrielsson, A.) 19–33 (Royal Swedish Academy of Music, 1987).

[9] Desain P, Honing H (2003). The formation of rhythmic categories and metric priming. Perception.

[10] Sadakata M, Desain P, Honing H (2006). The Bayesian way to relate rhythm perception and production. Music Percept..

[11] Schulze H (1989). Categorical perception of rhythmic patterns. Psychol. Res..

[12] Goldstone RL, Hendrickson AT (2010). Categorical perception. WIREs Cogn. Sci..

[13] Lomax A, Berkowitz N (1972). The evolutionary taxonomy of culture. Science.

[14] Brown S, Jordania J (2013). Universals in the world’s musics. Psychol. Music.

[15] Savage PE, Brown S, Sakai E, Currie TE (2015). Statistical universals reveal the structures and functions of human music. Proc. Natl Acad. Sci. USA.

[16] Mehr SA (2019). Universality and diversity in human song. Science.

[17] Savage, Patrick E., 'An Overview of Cross-Cultural Music Corpus Studies', in Daniel Shanahan, John Ashley Burgoyne, and Ian Quinn (eds), The Oxford Handbook of Music and Corpus Studies (online edn, Oxford Academic, 14 Feb. 2022).

[18] Kessler EJ, Hansen C, Shepard RN (1984). Tonal schemata in the perception of music in Bali and in the West. Music Percept..

[19] Perlman M, Krumhansl CL (1996). An experimental study of internal interval standards in Javanese and Western musicians. Music Percept..

[20] Krumhansl CL (2000). Cross-cultural music cognition: cognitive methodology applied to North Sami yoiks. Cognition.

[21] Curtis ME, Bharucha JJ (2009). Memory and musical expectation for tones in cultural context. Music Percept..

[22] Fritz T (2009). Universal recognition of three basic emotions in music. Curr. Biol..

[23] Laukka P, Eerola T, Thingujam NS, Yamasaki T, Beller G (2013). Universal and culture-specific factors in the recognition and performance of musical affect expressions. Emotion.

[24] Egermann H, Fernando N, Chuen L, McAdams S (2015). Music induces universal emotion-related psychophysiological responses: comparing Canadian listeners to Congolese pygmies. Front. Psychol..

[25] McDermott JH, Schultz AF, Undurraga EA, Godoy RA (2016). Indifference to dissonance in native Amazonians reveals cultural variation in music perception. Nature.

[26] Jacoby N, McDermott JH (2017). Integer ratio priors on musical rhythm revealed cross-culturally by iterated reproduction. Curr. Biol..

[27] Polak R (2018). Rhythmic prototypes across cultures: a comparative study of tapping synchronization. Music Percept..

[28] Jacoby N (2019). Universal and non-universal features of musical pitch perception revealed by singing. Curr. Biol..

[29] McPherson MJ (2020). Perceptual fusion of musical notes by native Amazonians suggests universal representations of musical intervals. Nat. Commun..

[30] Athanasopoulos G, Eerola T, Lahdelma I, Kaliakatsos-Papakostas M (2021). Harmonic organisation conveys both universal and culture-specific cues for emotional expression in music. PLoS ONE.

[31] Jakubowski K, Polak R, Rocamora M, Jure L, Jacoby N (2022). Aesthetics of musical timing: culture and expertise affect preferences for isochrony but not synchrony. Cognition.

[32] Mehr SA, Singh M, York H, Glowacki L, Krasnow MM (2018). Form and function in human song. Curr. Biol..

[33] Hilton CB (2022). Acoustic regularities in infant-directed speech and song across cultures. Nat. Hum. Behav..

[34] Locke S, Kellar L (1973). Categorical perception in a non-linguistic mode. Cortex.

[35] Burns EM, Ward WD (1978). Categorical perception—phenomenon or epiphenomenon: evidence from experiments in the perception of melodic musical intervals. J. Acoust. Soc. Am..

[36] Povel DJ (1981). Internal representation of simple temporal patterns. J. Exp. Psychol. Hum. Percept. Perform..

[37] Henrich J, Heine SJ, Norenzayan A (2010). The weirdest people in the world?. Behav. Brain Sci..

[38] Bartlett, F. C. *Remembering: A Study in Experimental and Social Psychology* (Cambridge Univ. Press, 1932).

[39] Griffiths TL, Kalish ML (2007). Language evolution by iterated learning with Bayesian agents. Cogn. Sci..

[40] Cooper, G. W. & Meyer, L. B. *The Rhythmic Structure of Music* (Univ. Chicago Press, 1960).

[41] Barrett HC (2020). Towards a cognitive science of the human: cross-cultural approaches and their urgency. Trends Cogn. Sci..

[42] Gabrielsson A, Bengtsson I, Gabrielsson B (1983). Performance of musical rhythm in 3/4 and 6/8 meter. Scand. J. Psychol..

[43] Garner, W. R. *The Processing of Information and Structure* (Lawrence Erlbaum, 1974).

[44] Fraisse, P. in *The Psychology of Music* Vol. 1 (ed. Deutsch, D.) 149–180 (Academic Press, 1982).

[45] Povel DJ, Essens P (1985). Perception of temporal patterns. Music Percept..

[46] Mead A (1992). Review of the development of multidimensional scaling. J. R. Stat. Soc. D.

[47] Jacoby N (2020). Cross-cultural work in music cognition: challenges, insights and recommendations. Music Percept..

[48] Gosling SD, Mason W (2015). Internet research in psychology. Annu. Rev. Psychol..

[49] Holzapfel A (2015). Relation between surface rhythm and rhythmic modes in Turkish makam music. J. New Music Res..

[50] England, N. M. *Music among the Z̳ũ’/‘wã-si and Related Peoples of Namibia, Botswana, and Angola* (Garland, 1995).

[51] Goldberg D (2015). Timing variations in two Balkan percussion performances. Empir. Musicol. Rev..

[52] Hannon EE, Soley G, Ullal S (2012). Familiarity overrides complexity in rhythm perception: a cross-cultural comparison of American and Turkish listeners. J. Exp. Psychol. Hum. Percept. Perform..

[53] Hannon EE, Trehub SE (2005). Metrical categories in infancy and adulthood. Psychol. Sci..

[54] Kubik, G. Africa. *Grove Music Online*10.1093/gmo/9781561592630.article.00268 (2021).

[55] Agawu VK (2006). Structural analysis or cultural analysis? Competing perspectives on the ‘Standard Pattern’ of West African rhythm. J. Am. Musicol. Soc..

[56] Kubik, G. *Angolan Traits in Black Music*, *Games and Dances of Brazil: A Study of African Cultural Extensions Overseas* Vol. 10 (Junta de Investigações Científicas do Ultramar, 1979).

[57] Washburne C (1997). The clave of jazz: a Caribbean contribution to the rhythmic foundation of an African-American music. Black Music Res. J..

[58] Toussaint, G. T. *The Geometry of Musical Rhythm: What Makes a ‘Good’ Rhythm Good?* (CRC, 2013).

[59] Rocamora, M. *Computational Methods for Percussion Music Analysis: The Afro-Uruguayan Candombe Drumming as a Case Study*. PhD thesis, Univ. de la República (2018).

[60] Spiegel MF, Watson CS (1984). Performance on frequency-discrimination tasks by musicians and non-musicians. J. Acoust. Soc. Am..

[61] Kishon-Rabin L, Amir O, Vexler Y, Zaltz Y (2001). Pitch discrimination: are professional musicians better than non-musicians?. J. Basic Clin. Physiol. Pharmacol..

[62] Fujioka T, Trainor LJ, Ross B, Kakigi R, Pantev C (2004). Musical training enhances automatic encoding of melodic contour and interval structure. J. Cogn. Neurosci..

[63] McPherson MJ, McDermott JH (2018). Diversity in pitch perception revealed by task dependence. Nat. Hum. Behav..

[64] Getz LM, Barton S, Kubovy M (2014). The specificity of expertise: for whom is the clave pattern the ‘key’ to salsa music?. Acta Psychol..

[65] Aschersleben G, Prinz W (1995). Synchronizing actions with events: the role of sensory information. Percept. Psychophys..

[66] Patel AD, Daniele JR (2003). An empirical comparison of rhythm in language and music. Cognition.

[67] Stobart H, Cross I (2000). The Andean anacrusis? Rhythmic structure and perception in Easter songs of Northern Potosí, Bolivia. Br. J. Ethnomusicol..

[68] Liu J, Hilton CB, Bergelson E, Mehr S (2023). Language experience predicts music processing in a half-million speakers of 54 languages. Curr. Biol..

[69] Norman-Haignere S, Kanwisher N, McDermott JH (2015). Distinct cortical pathways for music and speech revealed by hypothesis-free voxel decomposition. Neuron.

[70] Nettl, B. in *The Origins of Music* (eds Wallin, N. L. et al.) 463–472 (MIT Press, 2000).

[71] Feldman NH, Griffiths TL, Morgan JL (2009). The influence of categories on perception: explaining the perceptual magnet effect as optimal statistical inference. Psychol. Rev..

[72] Smith K, Kalish ML, Griffiths TL, Lewandowsky S (2008). Introduction: cultural transmission and the evolution of human behaviour. Phil. Trans. R. Soc. B.

[73] Jing X, Griffiths TL (2010). A rational analysis of the effects of memory biases on serial reproduction. Cogn. Psychol..

[74] Ravignani A, Delgado T, Kirby S (2017). Musical evolution in the lab exhibits rhythmic universals. Nat. Hum. Behav..

[75] Anglada-Tort M, Harrison PMC, Lee H, Jacoby N (2023). Large-scale iterated singing experiments reveal oral transmission mechanisms underlying music evolution. Curr. Biol..

[76] Zaslavsky N, Kemp C, Regier T, Tishby N (2018). Efficient compression in color naming and its evolution. Proc. Natl Acad. Sci. USA.

[77] Carr JW, Smith K, Culbertson J, Kirby S (2020). Simplicity and informativeness in semantic category systems. Cognition.

[78] Tierney AT, Russo FA, Patel AD (2011). The motor origins of human and avian song structure. Proc. Natl Acad. Sci. USA.

[79] Tillmann B, Bharucha JJ, Bigand E (2000). Implicit learning of tonality: a self-organizing approach. Psychol. Rev..

[80] Berlin, B. & Kay, P. *Basic Color Terms: Their Universality and Evolution* (Univ. California Press, 1969).

[81] Repp BH (1984). Categorical perception: issues, methods, findings. Speech Lang..

[82] Majid A, Kruspe N (2018). Hunter-gatherer olfaction is special. Curr. Biol..

[83] Large EW, Snyder JS (2009). Pulse and meter as neural resonance. Ann. N. Y. Acad. Sci..

[84] Apicella C, Norenzayan A, Henrich J (2020). Beyond WEIRD: a review of the last decade and a look ahead to the global laboratory of the future. Evol. Hum. Behav..

[85] Amir D, McAuliffe K (2020). Cross-cultural, developmental psychology: integrating approaches and key insights. Evol. Hum. Behav..

[86] Ravignani A, Thompson B, Grossi T, Delgado T, Kirby S (2018). Evolving building blocks of rhythm: how human cognition creates music via cultural transmission. Ann. N. Y. Acad. Sci..

[87] Boebinger D, Norman-Haignere SV, McDermott JH, Kanwisher N (2021). Cortical music selectivity does not require musical training. J. Neurophysiol..

[88] Summers JJ, Bell R, Burns BD (1989). Perceptual and motor factors in the imitation of simple temporal patterns. Psychol. Res..

[89] Repp BH, London J, Keller PE (2011). Perception–production relationships and phase correction in synchronization with two-interval rhythms. Psychol. Res..

[90] Kaplan T, Cannon J, Jamone L, Pearce M (2022). Modeling enculturated bias in entrainment to rhythmic patterns. PLoS Comp. Biol..

[91] Grahn JA, Brett M (2007). Rhythm and beat perception in motor areas of the brain. J. Cogn. Neurosci..

[92] Ozaki, Y. et al. Globally, songs and instrumental melodies are slower, higher, and use more stable pitches than speech [Stage 2 Registered Report]. Preprint at *PsyArXiv*10.24072/pci.rr.100469 (2023). 10.1126/sciadv.adm9797 PMC11095461 38748798

[93] London, J. *Hearing in Time: Psychological Aspects of Musical Meter* (Oxford Univ. Press, 2012).

[94] Repp BH (2003). Rate limits in sensorimotor synchronization with auditory and visual sequences: the synchronization threshold and the benefits and costs of interval subdivision. J. Motor Behav..

[95] London J, Himberg T, Cross I (2009). The effect of structural and performance factors in the perception of anacruses. Music Percept..

[96] Marchetti, C. C. Aristoxenus “Elements of Rhythm”: Text, translation, and commentary with a translation and commentary on POxy 2687 (Rutgers, The State University of New Jersey-New Brunswick, 2009).

[97] Brennan, B. Augustine’s “De musica”. *Vigiliae Christianae* **42**, 267–281 (1988).

[98] Descartes, R. Compendium of Music [trans. Walter Robert] (American Institute of Musicology, 1961).

[99] Euler, L. *Tentamen novae theoriae musicae* (Academiae Scientiarum, 1739).

[100] Parncutt R, Hair G (2018). Psychocultural theory of musical interval: bye bye Pythagoras. Music Percept..

[101] Serra X (2017). The computational study of a musical culture through its digital traces. Acta Musicol..

[102] Mehrabi N, Morstatter F, Saxena N, Lerman K, Galstyan A (2021). A survey on bias and fairness in machine learning. ACM Comput. Surv..

[103] McAuley JD, Jones MR, Holub S, Johnston HM, Miller NS (2006). The time of our lives: lifespan development of timing and event tracking. J. Exp. Psychol. Gen..

[104] Anglada-Tort M, Harrison PMC, Jacoby N (2022). REPP: a robust cross-platform solution for online sensorimotor synchronization experiments. Behav. Res. Methods.

[105] Bridges D, Pitiot A, MacAskill MR, Peirce JW (2020). The timing mega-study: comparing a range of experiment generators, both lab-based and online. PeerJ.

[106] Anwyl-Irvine A, Dalmaijer ES, Hodges N, Evershed JK (2021). Realistic precision and accuracy of online experiment platforms, web browsers, and devices. Behav. Res. Methods.

[107] Spearman C (1910). Correlation calculated from faulty data. Br. J. Psychol..

[108] Posch L (2022). Characterizing the global crowd workforce: a cross-country comparison of crowdworker demographics. Hum. Comput..

[109] Riester, J. *Canción y Producción en la Vida de un Pueblo Indígena: Los Chimane del Oriente Boliviano* (Los Amigos del Libro, 1978).

[110] Huanca, T. *Tsimane’ Oral Tradition, Landscape, and Identity in Tropical Forest* (Wa-Gui, 2008).

[111] Kempf, D. & Kempf, E. *Journey to Chimane Land* (Xulon, 2017).

[112] Lima, I. M. d. F. *Maracatus-Nação: Ressignificando Velhas Histórias* (Edições Bagaço, 2005).

[113] Tinhorão, J. R. *Os Sons dos Negros no Brasil: Cantos, Danças, Folguedos: Origens* 3rd edn (Editora 34, 2012).

[114] Ferreira L (2007). An afrocentric approach to musical performance in south black atlantic: The candombe drumming in Uruguay. Trans. Music Revista Transcultural de Música.

[115] Rice, T. *May It Fill Your Soul: Experiencing Bulgarian Music* (Univ. Chicago Press, 1994).

[116] Buchanan, D. A. *Performing Democracy: Bulgarian Music and Musicians in Transition* (Univ. Chicago Press, 2006).

[117] Polak R (2000). A musical instrument travels around the world: jenbe playing in Bamako, West Africa, and beyond. World Music.

[118] Hood M (1960). The challenge of ‘bi-musicality’. Ethnomusicology.

[119] Ingram C (2012). Tradition and divergence in southwestern China: Kam big song singing in the village and on the stage. Asia Pac. J. Anthropol..

[120] Friedman, J., Hastie, T. & Tibshirani, R. *The Elements of Statistical Learning* (Springer, 2009).

[121] Fraisse, P. *Les Structures Rythmiques* (Publications Univ. Louvain, 1956).

[122] Repp BH, London J, Keller PE (2013). Systematic distortions in musicians’ reproduction of cyclic three-interval rhythms. Music Percept..

[123] Repp BH, London J, Keller PE (2012). Distortions in reproduction of two-interval rhythms: when the ‘attractor ratio’ is not exactly 1:2. Music Percept..

[124] Corcoran C, Frieler K (2021). Playing it straight. Music Percept..

[125] Yates CM, Justus T, Atalay NB, Mert N, Trehub SE (2017). Effects of musical training and culture on meter perception. Psychol. Music.

[126] Polak R (2010). Rhythmic feel as meter: non-isochronous beat subdivision in jembe music from Mali. Music Theory Online.

[127] Polak, R. in *Hip Hop Africa: New African Music in a Globalizing World* (ed. Charry, E. S.) 261–281 (Indiana Univ. Press, 2012).

[128] Tukey, J. W. *Exploratory Data Analysis* Vol. 2, 39–43 (Addison-Wesley, 1977).

[129] Cooper, G. & Meyer, L. B. *The Rhythmic Structure of Music* (Univ. Chicago Press, 1963).

[130] Kolinski M (1973). A cross-cultural approach to metro-rhythmic patterns. Ethnomusicology.

[131] Bishara AJ, Hittner JB (2017). Confidence intervals for correlations when data are not normal. Behav. Res. Methods Instrum. Comput..

[132] Sanborn AN, Griffiths TL, Shiffrin RM (2010). Uncovering mental representations with Markov chain Monte Carlo. Cogn. Psychol..

[133] Harrison, P. et al. In *Advances in Neural Information Processing Systems* Vol. 33 (eds Larochelle, H. et al.) 10659–10671 (Curran Associates, 2020).

[134] Kirby S, Cornish H, Smith K (2008). Cumulative cultural evolution in the laboratory: an experimental approach to the origins of structure in human language. Proc. Natl Acad. Sci. USA.

[135] Xu J, Griffiths TL (2010). A rational analysis of the effects of memory biases on serial reproduction. Cogn. Psychol..

[136] London J, Burger B, Thompson M, Toiviainen P (2016). Speed on the dance floor: auditory and visual cues for musical tempo. Acta Psychol..

[137] Levitin DJ, Grahn JA, London J (2018). The psychology of music: rhythm and movement. Annu. Rev. Psychol..

[138] Arom S (2004). L’aksak: principes et typologie. Cah. Musiques Tradit..

[139] Dzhidzhev, T. *Problemi na Metroritŭma i Strukturata na Pesenniya Folklor* (Bŭlgarskata Akademiya na Naukite, 1981).

[140] Jacoby, N. et al. Data for commonality and variation in mental representations of music revealed by a cross-cultural comparison of rhythm priors in 15 countries. *OSF* https://osf.io/6zd4v/ (2024). 10.1038/s41562-023-01800-9 PMC11132990 38438653

