# Supplementary material for: Commonality and variation in mental representations of music revealed by a cross-cultural comparison of rhythm priors in 15 countries
Source: Nat Hum Behav. 2024 Mar 4;8(5):846–77. doi: 10.1038/s41562-023-01800-9 (PMC11132990; doi:10.1038/s41562-023-01800-9)
Supplement: Supplementary file 1 — Supplementary Tables 1–3 and Figs. 1–9. [file 41562_2023_1800_MOESM1_ESM.pdf]

nature human behaviour

Article

https://doi.org/10.1038/s41562-023-01800-9

Commonality and variation in mental representations of music revealed by a cross-cultural comparison of rhythm priors in 15 countries

In the format provided by the authors and unedited

Supplementary Information for Jacoby et al.

Supplementary Tables

Supplementary Table 1: *Demographic information for the fast tempo experiment.* ***Additional demographic information is provided in Supplementary Table 3.***

#	Group Name	Group abbreviation	Type	Continent	Country	City/Cities	N	Females	Ages
1	US(NYC)-NM	US.NY	NM	North America	USA	NYC	26	15	18-61 (mean = 38.77 std= 13.00)
2	US(NYC)-WM	US.NY	WM	North America	USA	NYC	26	14	18-64 (mean = 30.54 std= 11.29)
3	US(NYC)-LM	US.NY	LM	North America	USA	NYC	34	4	18-64 (mean = 36.94 std= 13.62)
4	Bulgaria-LM	BG	LM	Europe	Bulgaria	Pleven/Plovdiv/Sofia	23	3	20-59 (mean = 41.48 std= 10.51)
5	Bulgaria-DA	BG	DA	Europe	Bulgaria	Pleven/Plovdiv/Sofia	32	15	19-53 (mean = 33.31 std= 8.76)
6	Turkey-ST	TK	ST	Europe	Turkey	Istanbul	27	18	18-28 (mean = 20.67 std= 2.09)
7	Turkey-LM	TK	LM	Europe	Turkey	Istanbul/ Izmir	24	7	22-44 (mean = 31.75 std= 6.19)
8	Mali-LM	MA	NM	Africa	Mali	Bamako	20	1	25-58 (mean = 38.40 std= 9.95)
9	Mali-DA	MA	NM	Africa	Mali	Sagele	24	21	20-62 (mean = 37.67 std= 9.91)
10	Namibia-NM	NA	NM	Africa	Namibia	Spitzkoppe	16	9	18-46 (mean = 30.88 std= 8.45)
11	Namibia-LM	NA	NM	Africa	Namibia	Spitzkoppe	14	10	18-48 (mean = 27.79 std= 8.68)
12	India-NM	IN	ST	Asia	India	Mumbai	15	14	20-44 (mean = 27.13 std= 7.30)
13	India-LM	IN	LM	Asia	India	Mumbai	12	1	17-43 (mean = 25.67 std= 7.09)

Supplementary Table *2*: *Supplementary demographic information (main experiment)*

#	Group	Type	Country	City	Number of Languages Spoken	Main Languages	Literacy	Years Playing Instrument/ Singing	Main Instruments Played	Most favorite music	Years of formal education	Total Trials	Mean async.	Std async.
1	US.BO	ST	USA	Boston	1- 4 (mean = 1.87 std= 0.92)	English (100%), Spanish (47%), French (20%)	L	0- 4 (mean = 0.93 std= 1.17)	None (47%), Piano (27%), Guitar (20%)	Not reported	12-17 (mean = 15.13 std= 1.55)	431	-74.8 - -2.88 (mean = -41.71 std= 25.24)	39.8 - 70.6 (mean = 49.38 std= 10.03)
2	US.BO	WM	USA	Boston	1- 3 (mean = 1.57 std= 0.76)	English (100%), German (14%), Mandarin (14%)	L	9-47 (mean = 18.29 std= 9.54)	Piano (43%), Guitar (29%), Clarinet (14%)	Not reported	12-23 (mean = 16.36 std= 3.37)	529	-73.2 - 7.76 (mean = -24.23 std= 19.42)	36.5 - 50.1 (mean = 42.49 std= 4.12)
3	US.NY	NM	USA	NYC	1- 3 (mean = 1.23 std= 0.59)	English (100%), Spanish (8%), French (4%)	L	0- 9 (mean = 1.73 std= 2.86)	None (58%), Guitar (12%), Piano (12%)	Drake, Stevie wonder	12-21 (mean = 15.62 std= 2.39)	633	-120 - -15.1 (mean = -44.22 std= 26.01)	28.7 - 62.5 (mean = 47.67 std= 6.94)
4	US.NY	WM	USA	NYC	1- 5 (mean = 2.07 std= 1.17)	English (100%), French (30%), Spanish (26%)	L	10-42 (mean = 20.04 std= 8.66)	Piano (70%), Violin (30%), Guitar (15%)	Classical, Beethoven, Brahms, Jazz	12-24 (mean = 17.63 std= 2.96)	672	-61.8 - 8.68 (mean = -23.59 std= 15.89)	27.5 - 57.3 (mean = 40.22 std= 6.81)
5	US.NY	LM	USA	NYC	1- 3 (mean = 1.61 std= 0.77)	English (100%), Spanish (31%), French (6%)	L	8-59 (mean = 24.08 std= 12.62)	Piano (33%), Guitar (19%), Trombone (14%)	Jazz, Miles Davis, John Coltrane, Stravinsky, Bach, Beatles	12-25 (mean = 17.81 std= 2.86)	811	-43.1 - 23.8 (mean = -16.10 std= 14.87)	27.7 - 57.9 (mean = 38.98 std= 6.46)
6	BR	LM	Brazil	Recife	1- 2 (mean = 1.07 std= 0.26)	Portuguese (100%), English (7%)	L	5-20 (mean = 12.53 std= 4.78)	Brazilian percussion (100%),Guitar (13%), Cavaquinho (7%)	Rock, Brega, MPB, Reggae, Samba	9-20 (mean = 12.85 std= 3.39)	433	-58 - 11.6 (mean = -26.78 std= 17.26)	33.3 - 46.4 (mean = 39.21 std= 4.40)
7	BO.LP	ST	Bolivia	La Paz	2- 4 (mean = 2.38 std= 0.65)	English (100%), Spanish (100%), Portuguese (23%)	P	0-10 (mean = 3.42 std= 3.48)	Piano (38%), Guitar (31%), None (31%),	Rock, Electronic, Psytrance	13-18 (mean = 15.62 std= 1.80)	299	-89.2 - 9.76 (mean = -27.93 std= 25.38)	37.5 - 54 (mean = 46.22 std= 5.11)
8	BO.LP	NM	Bolivia	La Paz	1- 3 (mean = 2.17 std= 0.72)	Spanish (100%), Aymara (50%), English (42%), Quechua (17%)	P	0- 3 (mean = 0.79 std= 1.12)	None (58%), Drums (17%), Charango (8%)	Bolivian folk, Cumbia, Romantic, Salsa, Bachata, Reggaeton	4-20 (mean = 12.96 std= 5.06)	304	-42.9 - 33.4 (mean = -10.61 std= 24.62)	39.2 - 60.6 (mean = 51.06 std= 6.98)
9	BO.SB	NM	Bolivia	San Borja	1- 1 (mean = 1.00 std= 0.00)	Spanish (100%)	P	0-12 (mean = 1.76 std= 3.67)	Sing (65%), None (24%), Drum (12%)	Romantic, Cumbia, Ranchera, Reggaeton, La playa, Pop	5-19 (mean = 11.35 std= 4.03)	386	-64.6 - 24.7 (mean = -24.55 std= 23.14)	38.9 - 68.1 (mean = 52.81 std= 8.19)
10	BO.SC	NM	Bolivia	Santa Cruz	1- 1 (mean = 1.00 std= 0.00)	Spanish (100%)	L	0- 9 (mean = 1.24 std= 2.41)	Sing (65%), None (24%), Wind (24%)	Rock,Pop, Romantic, Reggeton, Cristiano, Latin pop	12-22 (mean = 15.41 std= 2.62)	376	-79.2 - 20.4 (mean = -21.03 std= 24.74)	45.3 - 62.9 (mean = 52.90 std= 4.94)
11	BO.TS	NM	Bolivia	Tsimane' Villages	1- 2 (mean = 1.23 std= 0.43)	Tsimane' (100%), Spanish (23%)	S	** (see below)	Drum (64%), Ricarica (64%), None (31%),	Hymns, Traditional	0-12 (mean = 3.42 std= 2.87)	792	-64.7 - 112 (mean = -6.52 std= 33.73)	52.6 - 80.3 (mean = 68.82 std= 7.58)
12	UY	ST	Uruguay	Montevideo	1- 4 (mean = 2.44 std= 0.64)	Spanish (100%), English (89%), Portuguese (26%)	L	0- 0 (mean = 0.00 std= 0.00)	None (100%)	Rock, Pop, Pop-rock, Coldplay, Imagine dragons, La vela puerca	15-22 (mean = 17.41 std= 1.80)	675	-89.6 - -6.72 (mean = -38.74 std= 25.47)	33.1 - 61.7 (mean = 46.48 std= 6.73)
13	UY	LM	Uruguay	Montevideo	1- 3 (mean = 1.32 std= 0.60)	Spanish (100%), Portuguese (19%), English (10%), Yoruba (3%)	L	7-60 (mean = 34.65 std= 13.51)	Candombe-Drums (77%), Percussion (61%), Piano (10%)	Candombe, Salsa, Samba, Jazz, Rock, Rubén Rada	6-18 (mean = 9.45 std= 2.63)	776	-74.4 - 10 (mean = -19.89 std= 18.18)	28 - 51.6 (mean = 37.82 std= 6.00)
14	UK	ST	UK	Durham	1- 4 (mean = 2.12 std= 0.95)	English (100%), French (21%), Mandarin (17%),	L	0- 8 (mean = 1.29 std= 1.88)	None (75%), Guitar (17%), Piano (17%)	Pop, Rock, Classical, Coldplay, Indie	12-21 (mean = 16.25 std= 2.17)	480	-132 - 19.5 (mean = -44.73 std= 31.09)	38.6 - 61.1 (mean = 49.88 std= 5.21)
15	UK	LM	UK	Durham/ Newcastle/ Edinburgh	1- 4 (mean = 1.48 std= 0.83)	English (100%), French (17%), German (7%)	L	6-45 (mean = 17.17 std= 9.93)	Piano (45%), Guitar (28%), Drums (21%)	Jazz, Rock, Funk, Pop, Classical, Vulfpeck	12-20 (mean = 16.41 std= 2.26)	580	-74.6 - 0.684 (mean = -24.87 std= 17.77)	27 - 50 (mean = 38.78 std= 5.43)
16	UK	LM	Sweden	Stockholm	2- 6 (mean = 2.95 std= 1.09)	English (100%), Swedish (100%), German (27%),	L	10-40 (mean = 21.59 std= 10.28)	Violin (59%), Voice (32%), Piano (23%)	Swedish-folk, Folk, Bach, Beatles	12-2.2e+02 (mean = 27.40 std= 43.15)	455	-42.1 - 9.19 (mean = -16.89 std= 12.04)	30.4 - 46.6 (mean = 38.78 std= 4.03)
17	BG	LM	Bulgaria	Pleven/ Plovdiv/ Sofia	1- 4 (mean = 2.30 std= 0.88)	Bulgarian (100%), English (61%), Russian (48%)	L	10-50 (mean = 29.52 std= 11.41)	Gadulka (35%), Piano (26%), Kaval (22%),	Bulgarian folk, Rock	12-18 (mean = 15.13 std= 2.40)	687	-43 - 8.14 (mean = -18.59 std= 13.89)	32.3 - 44.5 (mean = 37.95 std= 3.67)

18	BG	DA	Bulgaria	Pleven/ Plovdiv/ Sofia	1- 5 (mean = 1.93 std= 0.87)	Bulgarian (100%), English (37%), Russian (26%)	L	0-34 (mean = 3.11 std= 7.29)	None (56%), Tupan (15%), Kaval (11%),	Bulgarian folk, Rock	12-32 (mean = 17.04 std= 5.61)	780	-75.7 - -0.586 (mean = -27.20 std= 18.69)	32.9 - 55.5 (mean = 42.39 std= 5.79)
19	TK	ST	Turkey	Istanbul	2- 3 (mean = 2.19 std= 0.40)	English (100%), Turkish (100%), French (11%)	L	0-11 (mean = 4.31 std= 3.59)	None (15%), Baglama (11%), Guitar (11%)	Pop/rock, Turkish folk, Rock, Pop, Sezen Aksu	13-21 (mean = 14.89 std= 1.89)	746	-79.9 - 10.7 (mean = -26.93 std= 20.32)	31.2 - 55.5 (mean = 43.67 std= 6.27)
20	TK	LM	Turkey	Istanbul / Ismir	1- 4 (mean = 1.96 std= 0.86)	Turkish (100%), English (67%), German (8%)	L	9-30 (mean = 17.00 std= 5.85)	Baglama (25%), Oud (21%), Bendir (13%),	Turkish folk, Pop/rock, Pop, Jazz, Turkish classical, Turkish pop	14-25 (mean = 18.71 std= 3.38)	617	-41.1 - 12.3 (mean = -18.21 std= 12.36)	31.2 - 45.9 (mean = 38.35 std= 4.25)
21	MA	ST	Mali	Bamako	2- 7 (mean = 3.45 std= 1.09)	French (100%), Bambara (97%), English (61%), Sounrhai (16%)	L	0-15 (mean = 1.10 std= 3.45)	None (84%), Voice (6%),	Malian rap, Malian pop, Oumou Sangaré, Salif Keita, Sidiki Diabaté, Traditional malian	13-20 (mean = 15.68 std= 1.62)	636	-58.9 - 20.6 (mean = -22.12 std= 21.88)	31.3 - 59.4 (mean = 44.70 std= 6.79)
22	MA	LM	Mali	Bamako	1- 4 (mean = 2.03 std= 0.78)	Bambara (100%), French (69%), Kasonka (14%)	P	7-57 (mean = 25.93 std= 12.45)	Dundun (97%), Jembe (90%), Ngoni (14%),	Traditional malian, Malian pop, Griot, Babani Koné, Salif Keita	0-17 (mean = 6.10 std= 4.20)	589	-34.7 - 2.59 (mean = -11.40 std= 8.54)	22.9 - 49.9 (mean = 34.03 std= 5.92)
23	MA	DA	Mali	Sagele	1- 2 (mean = 1.09 std= 0.29)	Maninka (100%), French (9%)	P	0- 3 (mean = 0.09 std= 0.51)	None (47%), Ji-Dunun (29%), Voice (18%),	Salif Keita, Oumou Sangaré, Sekouba Diabaté, Traditional Malian, Guinean pop, Malian music	0-12 (mean = 3.85 std= 3.89)	834	-52.4 - 10.7 (mean = -16.69 std= 13.86)	33.2 - 57.6 (mean = 40.83 std= 5.30)
24	BW.SA	LM	Botswana	D'Kar	1- 2 (mean = 1.59 std= 0.51)	English (59%)	P	10-59 (mean = 29.73 std= 14.73)	Voice (59%), Tagadire (35%), Thumbpiano (18%)	Not reported	Not reported	170	-51.9 - 12.9 (mean = -9.16 std= 15.81)	25.8 - 49.5 (mean = 39.96 std= 5.90)
25	BW.EA	LM	Botswana	Etsha	1- 2 (mean = 1.14 std= 0.36)	English (14%)	P	7-46 (mean = 16.36 std= 9.79)	Drums (71%), Voice (50%), Fingerguitar (7%)	Not reported	Not reported	127	-52.3 - 2.03 (mean = -20.14 std= 17.33)	32.3 - 54 (mean = 42.11 std= 5.57)
26	NA	NM	Namibia	Spitzkoppe	1- 3 (mean = 1.75 std= 0.58)	Afrikaans (94%), English (75%), Oshivambo (6%)	P	0- 8 (mean = 3.91 std= 2.76)	Singing (88%), Drums (13%), None (13%)	Ma/Gaisa, Gospel, T-bozz & Staika, Damara, Kalux	Not reported	370	-76.4 - 19.6 (mean = -25.14 std= 25.15)	41.4 - 68 (mean = 47.64 std= 6.51)
27	NA	LM	Namibia	Spitzkoppe	1- 3 (mean = 2.00 std= 0.55)	English (93%), Afrikaans (79%), German (14%)	P	4-20 (mean = 10.86 std= 4.19)	Singing (100%), Drums (21%), Trumpet (14%),	T-bozz & Staika, Damara, Traditional Damara, R&B	Not reported	344	-73.5 - 19.1 (mean = -25.84 std= 25.98)	39.3 - 59.6 (mean = 46.71 std= 5.36)
28	IN	NM	India	Mumbai	2- 5 (mean = 2.73 std= 0.96)	English (100%), Hindi (80%), Marathi (33%)	L	0- 1 (mean = 0.38 std= 0.48)	None (87%), Guitar (7%), Keyboard (7%)	A. r. Rahman, Arijit Singh, Bollywood, Shreya Ghoshal, Ilaiyaraaja	14-23 (mean = 17.60 std= 2.97)	378	-76.3 - -12.7 (mean = -39.15 std= 23.40)	37.3 - 55.7 (mean = 47.23 std= 5.35)
29	IN	LM	India	Mumbai	1- 4 (mean = 2.88 std= 0.86)	English (94%), Hindi (94%), Marathi (35%)	L	6-25 (mean = 11.68 std= 5.24)	Tabla (47%), Voice (18%), Flute (12%),	Zakir Hussain, Bhimsen Joshi, Hariprasad Chaurasia, Indian Ocean, Rashid Khan, Shahid Parvez	4-26 (mean = 16.76 std= 5.47)	426	-61.8 - -1.54 (mean = -20.28 std= 14.77)	31.9 - 47.2 (mean = 37.69 std= 4.69)
30	KR	ST	S.Korea	Daejeon	1- 1 (mean = 1.00 std= 0.00)	Not-reported (100%)	L	0- 9 (mean = 3.00 std= 3.48)	None (40%), Piano (40%), Guitar (7%)	K-pop, Pop, Classical music, Hip-hop, Jazz, Rock	Not reported	450	-45.2 - 0.483 (mean = -20.15 std= 14.60)	35.3 - 54.2 (mean = 44.19 std= 4.67)
31	KR	WM	S.Korea	Daejeon	1- 1 (mean = 1.00 std= 0.00)	Not-reported (100%)	L	5-22 (mean = 12.15 std= 5.34)	Piano (31%), String (31%), Vocal (31%)	Classical music, K-pop, Jazz, Pop, Hip-hop, Rock	Not reported	390	-38.9 - 19.4 (mean = -17.55 std= 16.54)	32.4 - 58.4 (mean = 42.87 std= 6.17)
32	KR	LM	S.Korea	Jeonju	1- 1 (mean = 1.00 std= 0.00)	Not-reported (100%)	L	5-15 (mean = 8.23 std= 2.74)	Wind (54%), Percussion (15%), String (15%)	K-pop, Hip-hop, Jazz, Classical music, Pop, Traditional Korean music	Not reported	388	-30.1 - -0.944 (mean = -19.88 std= 9.64)	38.7 - 49.9 (mean = 44.91 std= 3.49)
33	JP	ST	Japan	Tokyo / Fujisawa	1- 4 (mean = 1.65 std= 0.99)	Japanese (100%), English (30%), Chinese (5%)	L	0- 3 (mean = 0.35 std= 0.88)	None (75%), Acoustic Guitar (5%), Piano (5%)	Not reported	Not reported	428	-107 - 37.8 (mean = -42.35 std= 28.91)	39 - 58.9 (mean = 49.24 std= 5.08)
34	JP	WM	Japan	Tokyo / Fujisawa	1- 4 (mean = 2.23 std= 1.09)	Japanese (100%), English (69%), Chinese (8%)	L	5-20 (mean = 12.77 std= 5.18)	Piano (77%), Vocal (23%), Wadaiko (23%)	Not reported	Not reported	261	-50.6 - -8.25 (mean = -25.64 std= 12.60)	39.2 - 50 (mean = 44.23 std= 3.07)
35	JP	LM	Japan	Tokyo / Fujisawa	1- 4 (mean = 1.58 std= 0.99)	Japanese (100%), English (27%), French (4%)	L	7-21 (mean = 13.35 std= 3.97)	Shamisen (73%), Koto (46%), Piano (38%),	Not reported	Not reported	529	-61.8 - 7.18 (mean = -20.13 std= 19.45)	37.9 - 56.2 (mean = 45.45 std= 4.84)
36	CN	NM	China	Guizhou Province	1- 2 (mean = 1.77 std= 0.42)	Dong (100%), Mandarin (78%)	P	0-26 (mean = 1.68 std= 5.23)	None (80%), Voice (20%)	Not reported	Not reported	575	-100 - 11.4 (mean = -28.96 std= 21.84)	38.7 - 64.4 (mean = 50.15 std= 6.00)
37	US	OL	USA	Online	1 - 4 (mean = 1.46 std= 0.79)	English (100%), Spanish (11%), German (6%),	L	0 - 30 (mean = 5.06 std= 6.75)	none (46%), piano (26%), guitar (17%),	Rock, Pop, Beatles, Taylor Swift, Pop/rock	8 - 22 (mean = 16.25 std= 2.78)	948	-117 - 17 (mean = -37.00 std= 27.65)	31.5 - 72.5 (mean = 52.48 std= 9.66)
38	BR	OL	Brazil	Online	1 - 4 (mean = 2.39 std= 0.69)	Portuguese (100%), English (93%), Spanish (36%)	L	0 - 20 (mean = 3.52 std= 5.27)	none (43%), guitar (32%), piano (14%),	Rock, Pop, Heavy metal	11 - 25 (mean = 16.19 std= 3.57)	413	-85.8 - 5.91 (mean = -33.15 std= 21.40)	38.9 - 70.9 (mean = 55.40 std= 7.44)
39	IN	OL	India	Online	2 - 5 (mean = 3.11 std= 0.92)	English (100%), Hindi (76%), Tamil (47%), Malayalam (24%)	L	0 - 20 (mean = 3.06 std= 5.02)	none (53%), guitar (21%), harmonium (8%)	Pop, Rock, A. r. Rahman, Classic, Rap	8 - 22 (mean = 17.48 std= 2.84)	569	-97.2 - 27.2 (mean = -38.30 std= 31.32)	31.8 - 88 (mean = 61.96 std= 11.85)

Literacy- L- literate participants; P- partial literacy: some participants are not fully literate; S- some participants had some literacy. **- Two participants reported regularly playing an instrument. 34 participants reported playing an instrument at least once.

Supplementary Table 3*: Supplementary demographic information (fast tempo experiment).*

#	Group	Type	Country	City	Number Of Languages Spoken	Main Languages Spoken	Literacy	Years Playing Instrument/Singing	Main Instruments Played	Most Mentioned Favorite Artists or Musical Genres	Years of formal education	Total Trials	Mean Asynchrony	Std Asynchrony
1	US.NY	NM	USA	NYC	1- 3 (mean = 1.23 std= 0.59)	English (100%), Spanish (8%), French (4%)	L	0- 9 (mean = 1.73 std= 2.86)	None (58%), guitar (12%), piano (12%)	Drake, Stevie Wonder	12-21 (mean = 15.62 std= 2.39)	631	-32.7 - 15.8 (mean = -9.39 std= 13.75)	17.9 - 32.4 (mean = 25.75 std= 3.39)
2	US.NY	WM	USA	NYC	1- 4 (mean = 1.96 std= 1.04)	English (100%), French (27%), Spanish (23%)	L	10-42 (mean = 19.69 std= 8.64)	Piano (73%), violin (27%), guitar (15%)	Classical, Beethoven, Brahms, Jazz	12-24 (mean = 17.54 std= 2.98)	636	-49.8 - 12.6 (mean = -11.79 std= 13.10)	16.5 - 28.5 (mean = 22.17 std= 3.16)
3	US.NY	LM	USA	NYC	1- 3 (mean = 1.59 std= 0.74)	English (100%), Spanish (29%), French (6%),	L	8-59 (mean = 24.65 std= 12.76)	Piano (32%), guitar (21%), bass (12%)	Jazz, Miles Davis, Bach, Stravinsky	12-25 (mean = 17.83 std= 2.95)	773	-35.1 - 25.8 (mean = -3.86 std= 12.62)	14.3 - 30.7 (mean = 21.98 std= 3.54)
4	BG	LM	Bulgaria	Pleven/ Plovdiv/Sofia	1- 4 (mean = 2.30 std= 0.88)	Bulgarian (100%), English (61%), Russian (48%)	L	10-50 (mean = 29.52 std= 11.41)	Gadulka (35%), piano (26%), kaval (22%), voice (17%)	Bulgarian folk, Rock	12-18 (mean = 15.13 std= 2.40)	641	-30.7 - 9.49 (mean = -10.61 std= 11.01)	17.5 - 24.3 (mean = 20.41 std= 1.93)
5	BG	DA	Bulgaria	Pleven/ Plovdiv/Sofia	1- 5 (mean = 2.03 std= 1.00)	Bulgarian (100%), English (44%), Russian (31%)	L	0-34 (mean = 3.03 std= 6.76)	None (53%), tupan (19%), kaval (9%), voice (6%)	Bulgarian folk, Rock, Michael Jackson	12-32 (mean = 17.25 std= 5.25)	961	-34.9 - 9.93 (mean = -14.71 std= 9.95)	15.4 - 28.1 (mean = 22.55 std= 2.97)
6	TK	ST	Turkey	Istanbul	2- 3 (mean = 2.19 std= 0.40)	Turkish (100%), English (100%), French (11%)	L	0-11 (mean = 4.31 std= 3.59)	None (15%), baglama (11%), guitar (11%), ney (11%)	Pop/rock, Turkish folk, Rock, Pop, Sezen Aksu	13-21 (mean = 14.89 std= 1.89)	741	-24.2 - 15.3 (mean = -4.55 std= 10.58)	17.5 - 28.7 (mean = 23.23 std= 3.07)
7	TK	LM	Turkey	Istanbul / Izmir	1- 4 (mean = 1.96 std= 0.86)	Turkish (100%), English (67%), German (8%)	L	9-30 (mean = 17.00 std= 5.85)	Baglama (25%), oud (21%), bendir (13%), clarinet (13%)	Turkish folk, Pop/rock, Pop, Jazz, Turkish classical, Turkish pop	14-25 (mean = 18.71 std= 3.38)	616	-18 - 12.4 (mean = -5.75 std= 7.65)	16.4 - 24 (mean = 20.59 std= 2.05)
8	MA	LM	Mali	Bamako	1- 4 (mean = 2.00 std= 0.86)	Bambara (95%), French (65%)	P	7-42 (mean = 23.75 std= 11.62)	Dundun (95%), jembe (90%), tamanin (25%),	Traditional Malian, Malian pop, Griot, Salif Keita	0-17 (mean = 7.05 std= 4.31)	606	-8.9 - 7.19 (mean = -0.32 std= 4.68)	14 - 22.3 (mean = 16.98 std= 2.37)
9	MA	DA	Mali	Sagele	1- 2 (mean = 1.08 std= 0.28)	Maninka (100%), French (8%)	P	0- 3 (mean = 0.12 std= 0.61)	None (50%), ji-dunun (25%), voice (17%)	Salif Keita, Sekouba Diabaté, Traditional Malian, Guinean pop, Malian music	0-12 (mean = 2.96 std= 3.83)	604	-22.9 - 19.1 (mean = -2.85 std= 9.54)	18.2 - 28.2 (mean = 22.76 std= 2.25)
10	NA	NM	Nambia	Spitzkoppe	1- 3 (mean = 1.75 std= 0.58)	Afrikaans (94%), English (75%), Oshivambo (6%)	P	0- 8 (mean = 3.91 std= 2.76)	Singing (88%), drums (13%), none (13%)	Ma/Gaisa, Gospel, T-bozz & Staika, Damara, Kalux	not reported	361	-28.9 - 32.7 (mean = -5.69 std= 16.53)	20.9 - 33.5 (mean = 25.72 std= 3.31)
11	NA	LM	Namibia	Spitzkoppe	1- 3 (mean = 2.00 std= 0.55)	English (93%), Afrikaans (79%), German (14%)	P	4-20 (mean = 10.86 std= 4.19)	Singing (100%), drums (21%), trumpet (14%),	T-bozz & Staika, Damara, Traditional Damara, R&B	not reported	341	-33.2 - 29.8 (mean = -7.11 std= 17.47)	21.6 - 31.3 (mean = 25.81 std= 2.94)
12	IN	NM	India	Mumbai	2- 5 (mean = 2.73 std= 0.96)	English (100%), Hindi (80%), Marathi (33%),	L	0- 1 (mean = 0.38 std= 0.48)	None (87%), guitar (7%), keyboard (7%)	A. r. Rahman, Arijit Singh, Bollywood, Ilaiyaraaja, Shreya Ghoshal, Atif Aslam	14-23 (mean = 17.60 std= 2.97)	376	-24.1 - 12 (mean = -8.64 std= 10.67)	21.6 - 29.5 (mean = 25.39 std= 2.58)
13	IN	LM	India	Mumbai	2- 4 (mean = 2.75 std= 0.62)	English (100%), Hindi (100%), Marathi (42%), (8%)	L	7-20 (mean = 12.25 std= 3.74)	Tabla (67%), flute (17%), harmonium (17%)	Zakir Hussain, Hariprasad Chaurasia, Indian ocean	11-21 (mean = 16.00 std= 3.13)	300	-33.4 - 18.2 (mean = -3.88 std= 13.94)	16.7 - 23.2 (mean = 19.97 std= 1.92)

Literacy- L- literate participants; P- partial literacy: some participants are not fully literate; S- some participants had some literacy.

Supplementary Figures

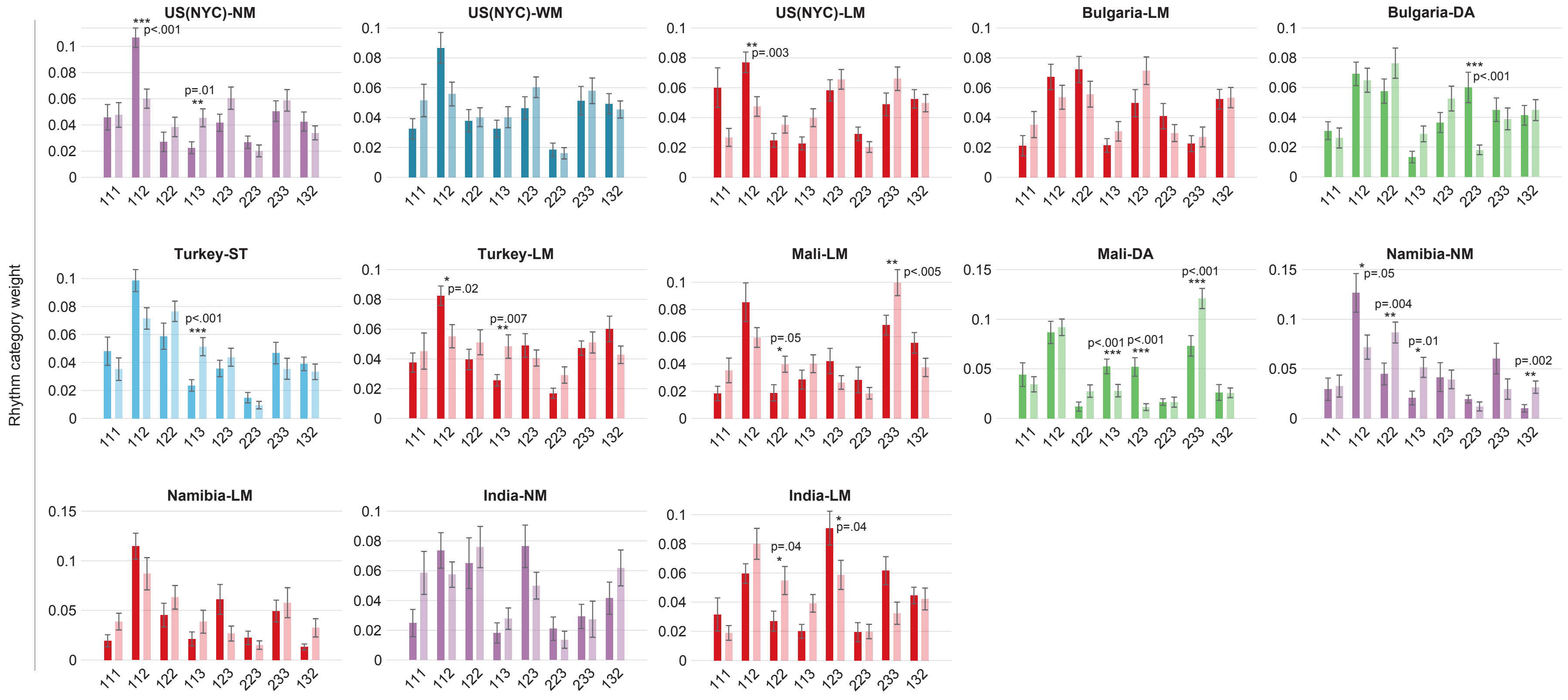

Supplementary Figure 1: *Category weights of the fast tempo experiment, compared to those from the slow tempo experiment.* Category weights from the fast and slow tempo experiments in 13 groups that performed both experiments. Darker colored bars plot category weights for the fast tempo; lighter colored bars plot category weights for the slow tempo. Asterisks mark statistical significance after Bonferroni correction for multiple comparisons (***: p<.001; **: p<.01; *: p<.05). Error bars plot SEM, derived from 1000 bootstrap samples.

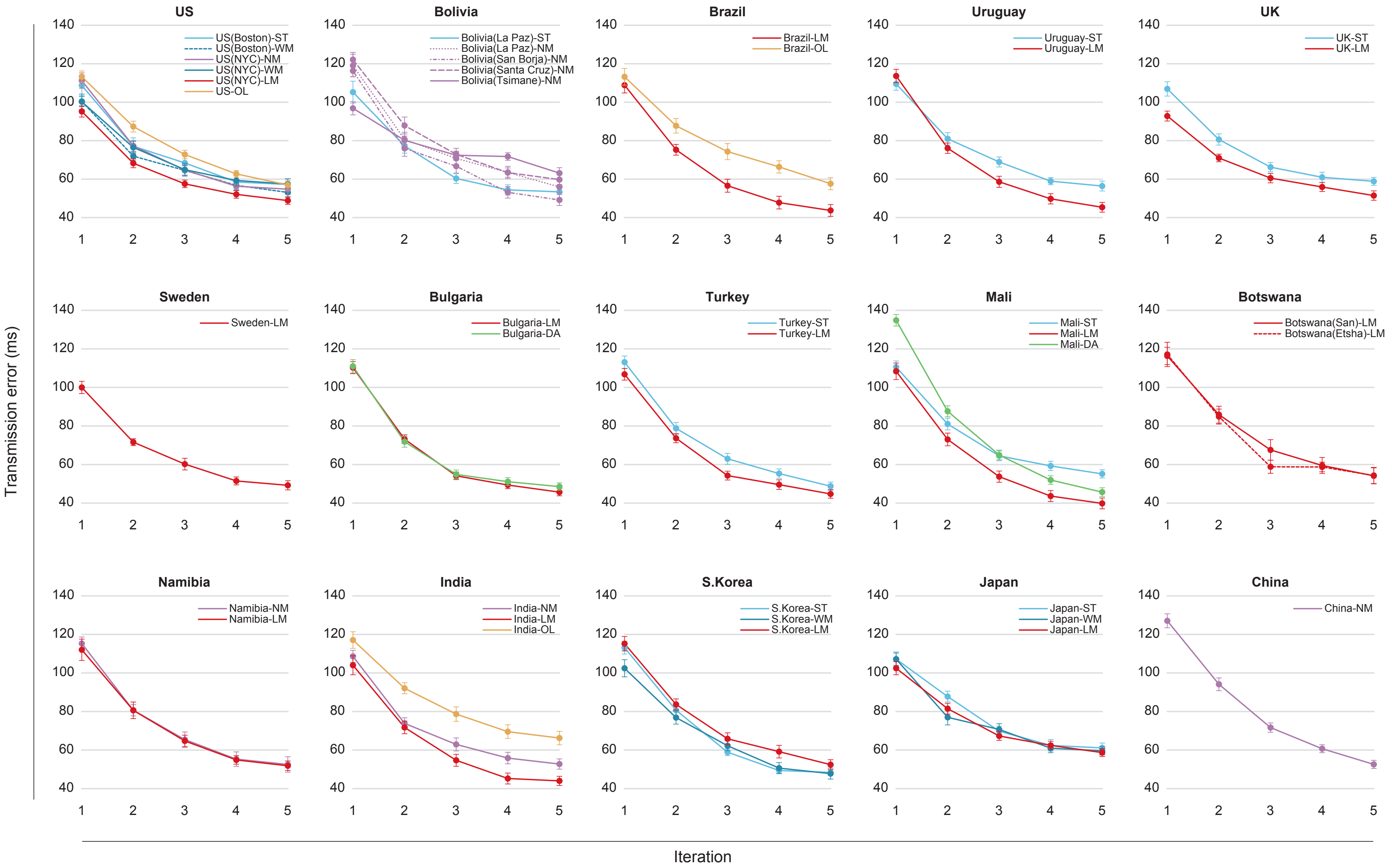

Supplementary Figure 2: *Transmission error in 39 groups*. Transmission error is a standard way to monitor the dynamics of serial reproduction experiments. As an error measure, we use the average distance in milliseconds between the seed and response in each iteration. Namely, the average across trials of $e = \sqrt{(s_1 - r_1)^2 + (s_2 - r_2)^2 + (s_3 - r_3)^2}$, where (s_1, s_2, s_3) and (r_1, r_2, r_3) are the stimulus and average response of each iteration, respectively (i.e., the response is averaged across the 10 repetitions within each iteration). Transmission error is expected to remain constant after convergence. The figure shows that the dynamics converged or nearly converged in all 39 groups by the fifth iteration. Data are presented as mean values +/- SEM across participants (see methods, number of participants is detailed in Extended Data Table 1).

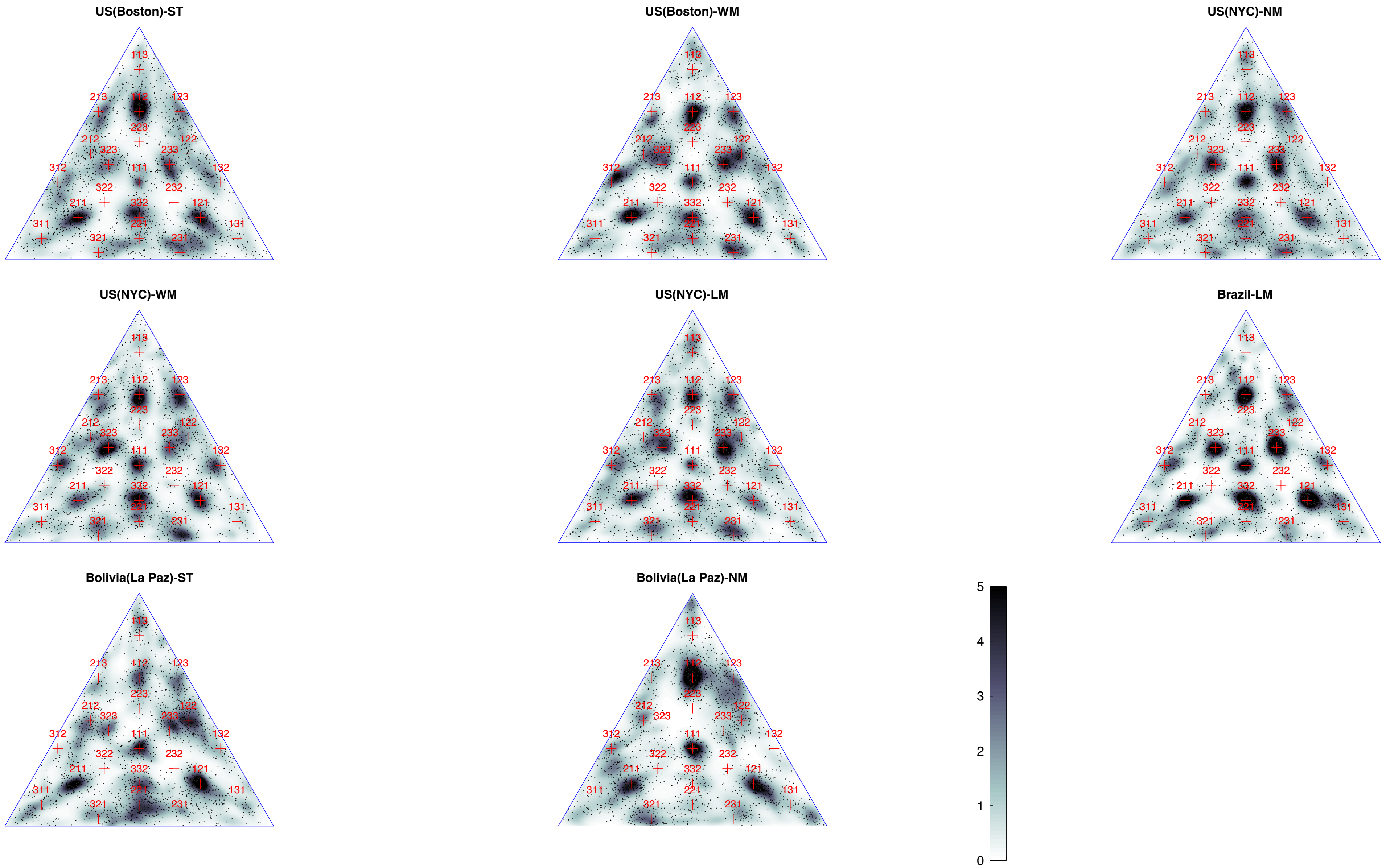

Supplementary Figure 3: *Rhythm priors from main experiment (1/5).* Kernel density estimates were clipped at a value of 5 (relative to uniform distribution; see Methods) in order to preserve the dynamic range for details at low density values.

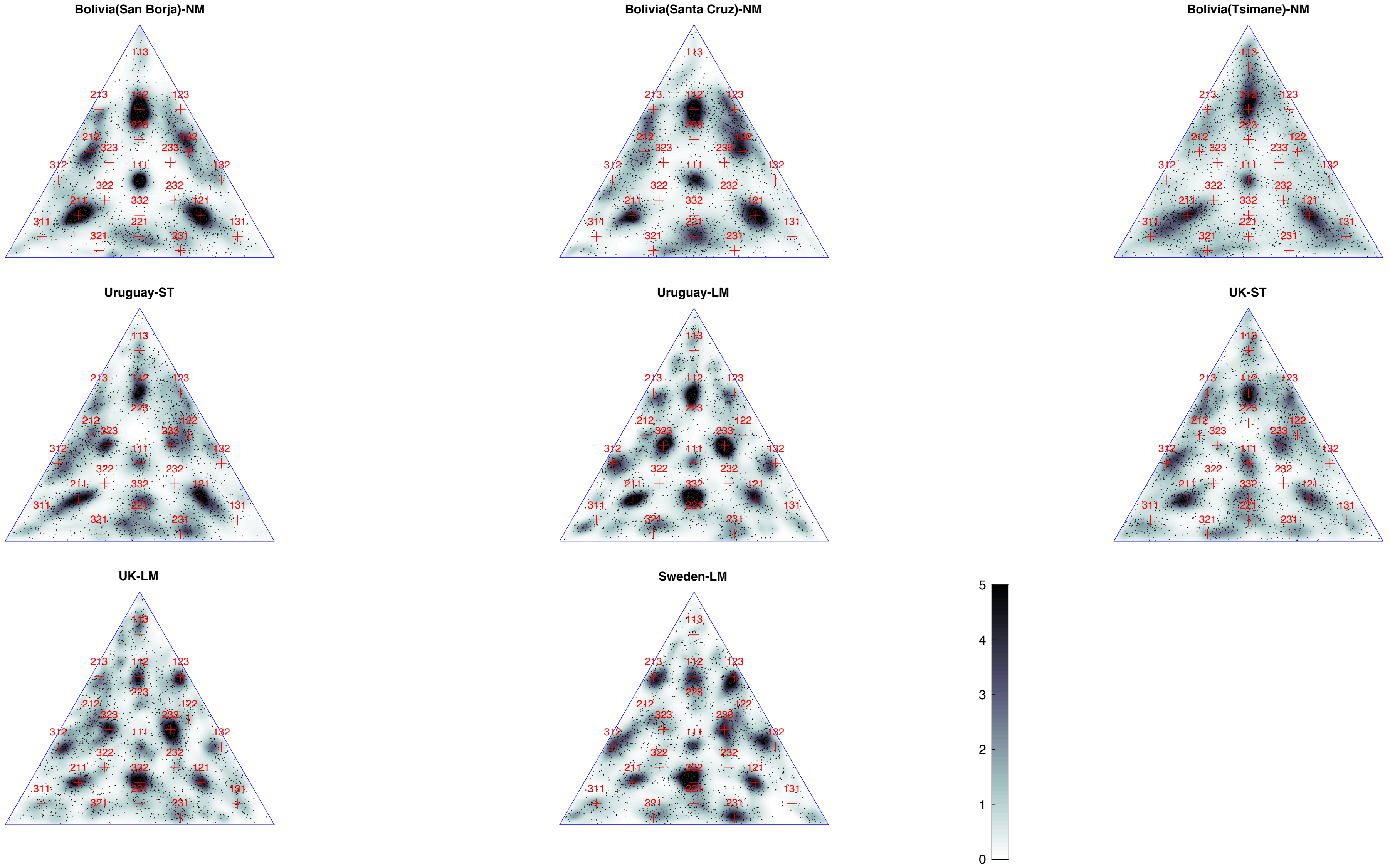

Supplementary Figure 4: *Rhythm priors from main experiment (2/5)*. Kernel density estimates were clipped at a value of 5 (relative to uniform distribution see Methods) in order to preserve the dynamic range for details at low density values.

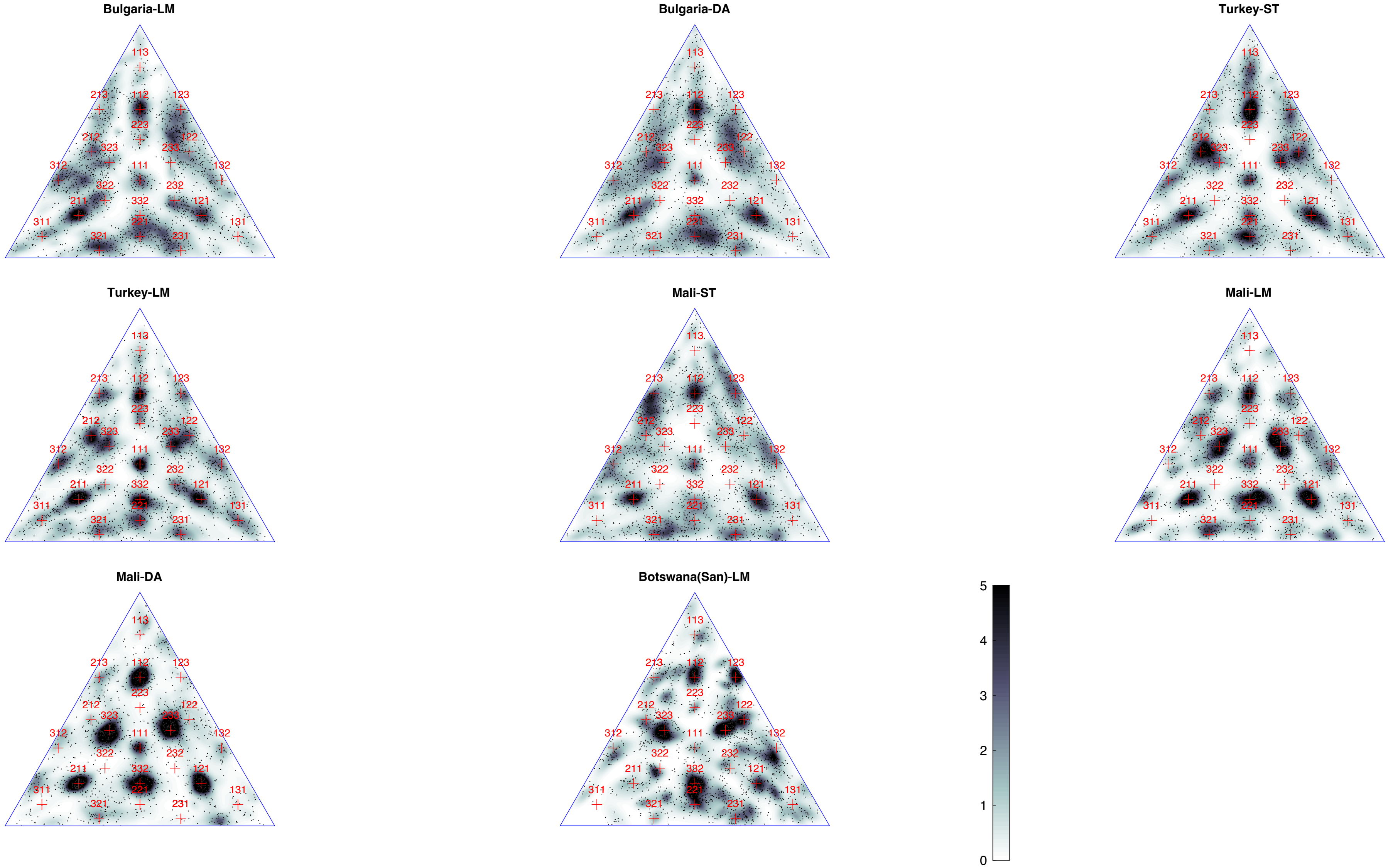

Supplementary Figure 5: *Rhythm priors from main experiment (3/5).* Kernel density estimates were clipped at a value of 5 (relative to uniform distribution see Methods) in order to preserve the dynamic range for details at low density values.

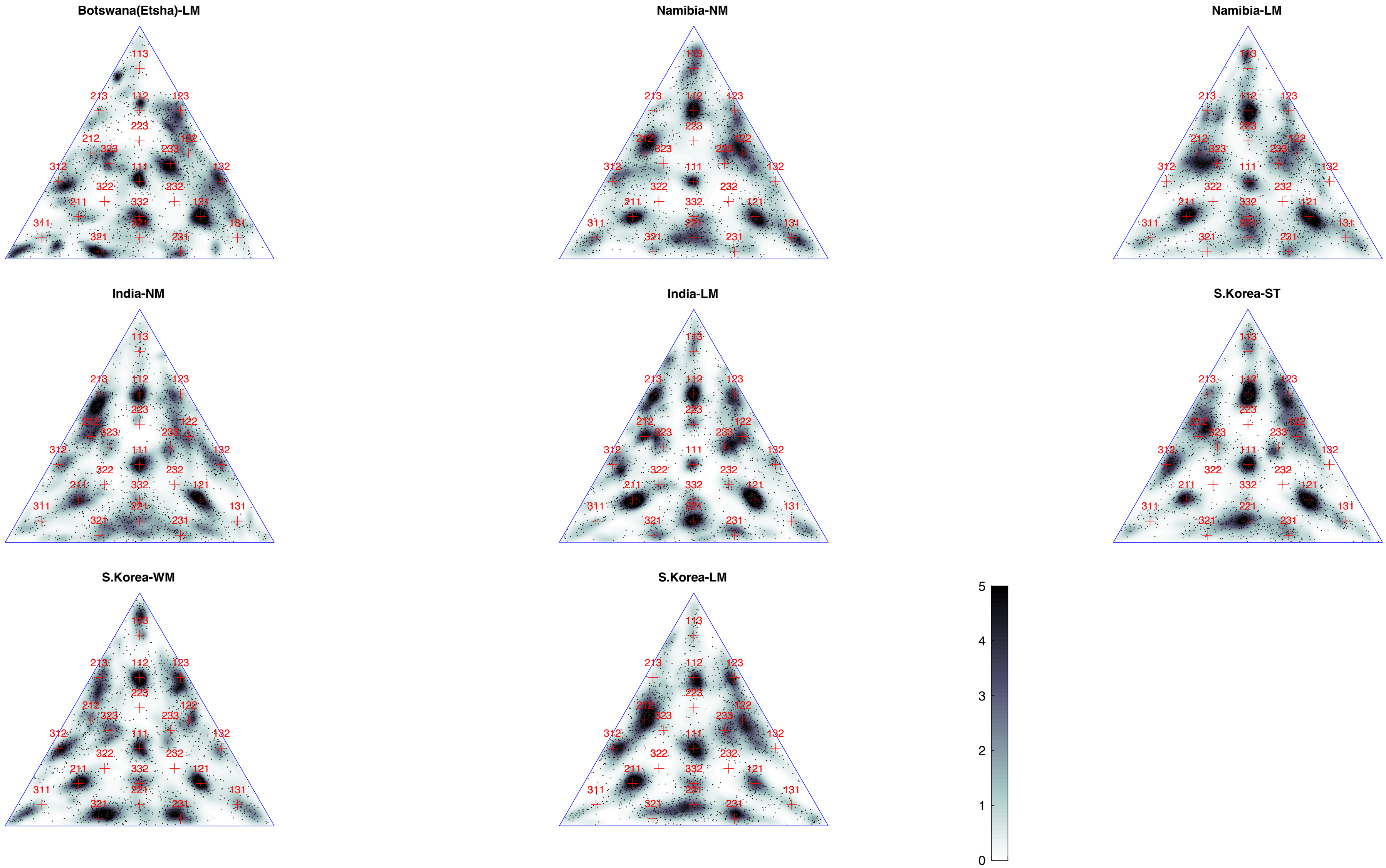

Supplementary Figure 6: *Rhythm priors from main experiment (4/5)*. Kernel density estimates were clipped at a value of 5 (relative to uniform distribution see Methods) in order to preserve the dynamic range for details at low density values.

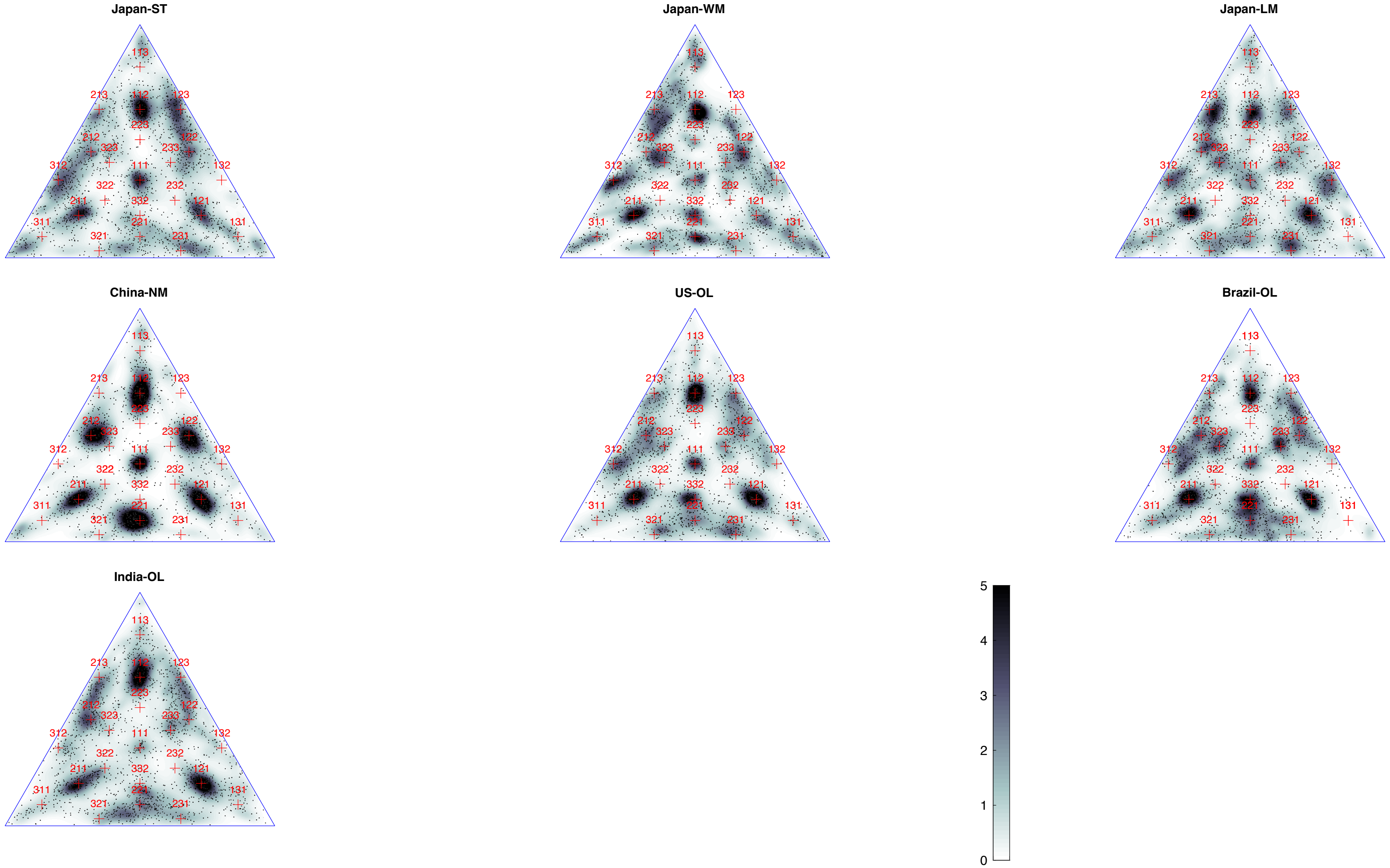

Supplementary Figure 7*: Rhythm priors from main experiment (5/5).* Kernel density estimates were clipped at a value of 5 (relative to uniform distribution see Methods) in order to preserve the dynamic range for details at low density values.

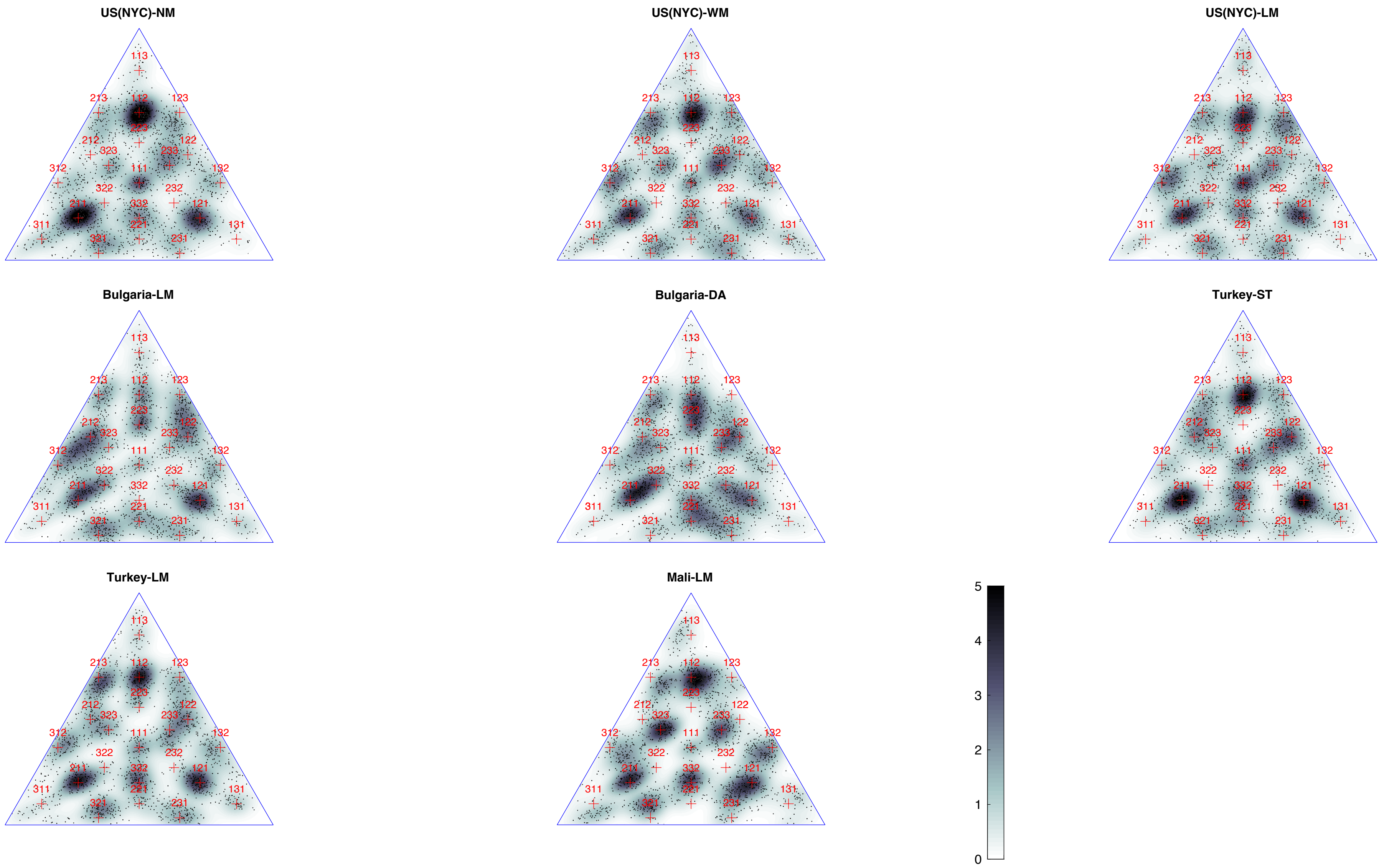

Supplementary Figure 8*: Rhythm priors from fast tempo experiment (1/2).* Kernel density estimates were clipped at a value of 5 (relative to uniform distribution see Methods) in order to preserve the dynamic range for details at low density values.

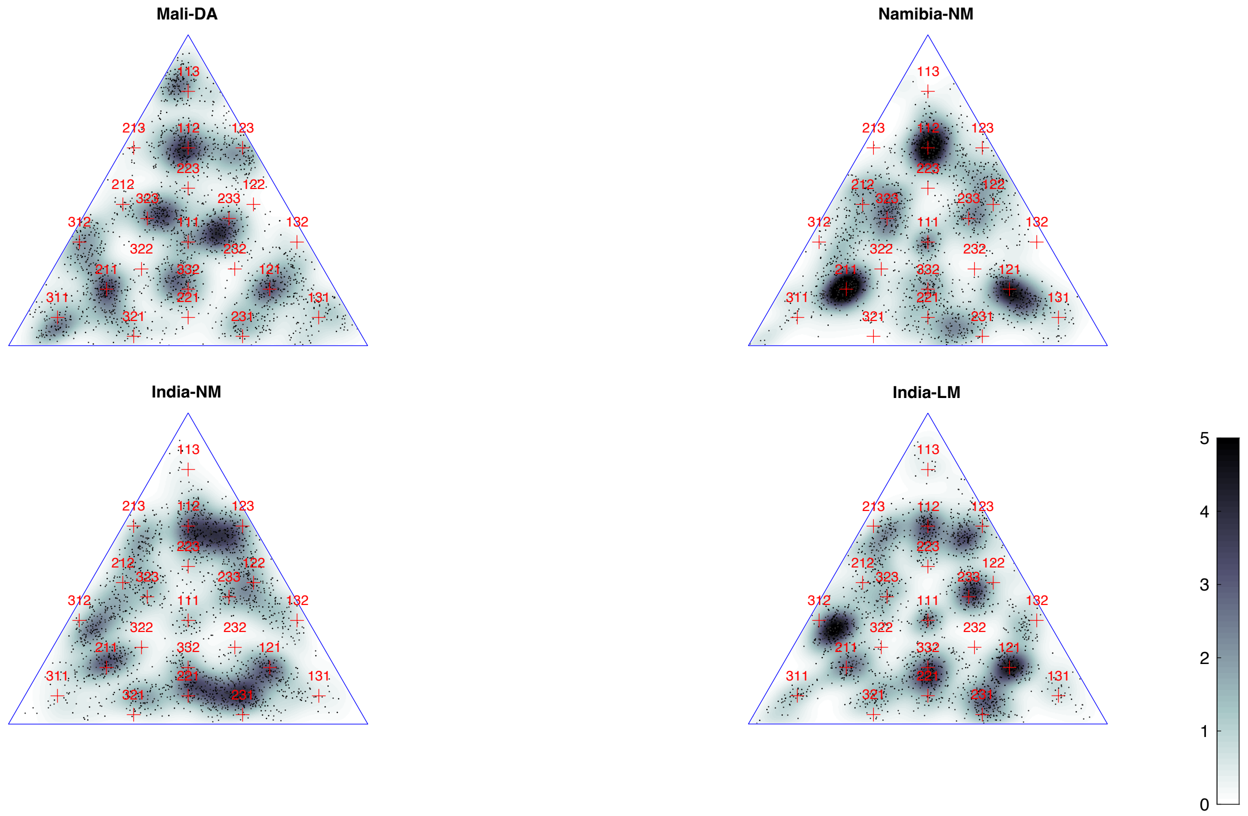

Supplementary Figure 9: *Rhythm priors from fast tempo experiment (2/2).* Kernel density estimates were clipped at a value of 5 (relative to uniform distribution see Methods) in order to preserve the dynamic range for details at low density values.
