# Supplementary material for: Commonality and variation in mental representations of music revealed by a cross-cultural comparison of rhythm priors in 15 countries
Source: Nat Hum Behav. 2024 Mar 4;8(5):846–77. doi: 10.1038/s41562-023-01800-9 (PMC11132990; doi:10.1038/s41562-023-01800-9)
Supplement: Supplementary file 2 — Reporting Summary [file 41562_2023_1800_MOESM2_ESM.pdf]

nature portfolio

Corresponding author(s): Nori Jacoby and Josh McDermott

Last updated by author(s): Oct 10, 2023

Reporting Summary

Nature Portfolio wishes to improve the reproducibility of the work that we publish. This form provides structure for consistency and transparency in reporting. For further information on Nature Portfolio policies, see our Editorial Policies and the Editorial Policy Checklist.

Statistics

For all statistical analyses, confirm that the following items are present in the figure legend, table legend, main text, or Methods section.

n/a	Confirmed	
☐	☒	The exact sample size (*n*) for each experimental group/condition, given as a discrete number and unit of measurement
☐	☒	A statement on whether measurements were taken from distinct samples or whether the same sample was measured repeatedly
☐	☒	The statistical test(s) used AND whether they are one- or two-sided<br>*Only common tests should be described solely by name; describe more complex techniques in the Methods section.*
☒	☐	A description of all covariates tested
☐	☒	A description of any assumptions or corrections, such as tests of normality and adjustment for multiple comparisons
☐	☒	A full description of the statistical parameters including central tendency (e.g. means) or other basic estimates (e.g. regression coefficient) AND variation (e.g. standard deviation) or associated estimates of uncertainty (e.g. confidence intervals)
☐	☒	For null hypothesis testing, the test statistic (e.g. *F*, *t*, *r*) with confidence intervals, effect sizes, degrees of freedom and *P* value noted<br>*Give P values as exact values whenever suitable.*
☒	☐	For Bayesian analysis, information on the choice of priors and Markov chain Monte Carlo settings
☒	☐	For hierarchical and complex designs, identification of the appropriate level for tests and full reporting of outcomes
☒	☐	Estimates of effect sizes (e.g. Cohen's *d*, Pearson's *r*), indicating how they were calculated

Our web collection on statistics for biologists contains articles on many of the points above.

Software and code

Policy information about availability of computer code

Data collection	Matlab2018a; Code and instructions for running the paradigm is provided in the OSF repository associated with the project (https://osf.io/6zd4v/)
Data analysis	Matlab2022a; Code for reproducing all analyses and results graphs is also provided in the OSF repository associated with the project (https://osf.io/6zd4v/)

For manuscripts utilizing custom algorithms or software that are central to the research but not yet described in published literature, software must be made available to editors and reviewers. We strongly encourage code deposition in a community repository (e.g. GitHub). See the Nature Portfolio guidelines for submitting code & software for further information.

Data

Policy information about availability of data

All manuscripts must include a data availability statement. This statement should provide the following information, where applicable:

- Accession codes, unique identifiers, or web links for publicly available datasets
- A description of any restrictions on data availability
- For clinical datasets or third party data, please ensure that the statement adheres to our policy

Raw data for all groups is provided in the OSF repository associated with the project (https://osf.io/6zd4v/).

Field-specific reporting

Please select the one below that is the best fit for your research. If you are not sure, read the appropriate sections before making your selection.

☐ Life sciences ☒ Behavioural & social sciences ☐ Ecological, evolutionary & environmental sciences

For a reference copy of the document with all sections, see nature.com/documents/nr-reporting-summary-flat.pdf

Behavioural & social sciences study design

All studies must disclose on these points even when the disclosure is negative.

Study description

We measured a signature of mental representations of rhythm in groups around the world. The study is based on quantitative methods and data analysis.

Research sample

We tested 39 participant groups spanning five continents and 15 countries . Overall, we recruited 923 participants (792 were run face to face and 131 online). The rationale for the chosen study sample was based on testing groups that provide a strong test of commonalities across cultures. We included groups from both industrialized and non-industrialized societies, as well as groups of local musicians who do not play Western music. We also tested groups of musicians and dancers where possible, as these populations would be expected to have substantial exposure to particular musical styles. In addition, we tested university students and online participants in a number of countries in order to assess potential effects of exposure to Western music and culture, which we expected would tend to be correlated with university attendance and internet access. The groups tested were also determined in part by practical constraints (testing time and access to particular populations). Additional information about the groups is provided in the section "Criteria for group selection" in the Methods. See Table 1 for detailed demography of all 39 groups. Additional demographic information is provided in Table E1.

Sampling strategy

Participants were recruited based on criteria that depended on the group type (Students, Musicians, Dancers, Non-musicians and Online participants). The criteria for each group type are described in section "Definition of group types" in the Methods. We used convenience sampling subject to the constraints of the group definition. Sample size was determined by power analysis based on test-retest reliability test of the kernel density estimate, using data collected for a previous publication (Jacoby and McDermott 2017).

Data collection

In-person experiments typically involved the participant, the experimenter and a translator (if the experimenter was not a native speaker of a language spoken fluently by the participant). The experimenter could hear the stimulus and the participant's responses; we found this facilitated high quality data collection as otherwise it was difficult to assess whether the participant was following instructions. Data were collected by a large set of experimenters. Some of the experimenters were not fully blind to the main hypotheses of the study, but many were.
In the online sessions, there was no experimenter present (the participant completed the experiment using their browser).

Timing

Data was collected between 2015-2021: Botswana (9/2016-10/2016), South Korea (1/2017-9/2017), Bolivia (6/2015-8/2019), Brazil (9/2017-10/2017), Mali (12/2017-1/2018), Bulgaria (3/2018-7/2018), China (5/2018), Uruguay (4/2018-5/2018), Japan (6/2018), UK (5/2018-8/2018), Sweden (10/2018), Turkey (4/2018-1/2019), India (9/2018-5/2019), Namibia (4/2019), Boston (5/2015-12/2015) and in NYC (6/2017-9/2019).

Data exclusions

We pre-determined (in a previous publication - Jacoby and McDermott, 2017) accuracy criteria for the participant's tapped response to be counted as a valid tap, iteration or trial. We defined a valid tap as occurring within a +/-150ms window of the stimulus after correcting for the mean asynchrony of the iteration. We defined a valid iteration as one which had a three-tap response for 7 or more of the 10 repetitions and whose average response (r_1,r_2,r_3) was not situated far beyond the region we defined for human-producible rhythms (i.e., did not contain an interval shorter than 285 ms of the overall duration of 2000 ms). Trials with 3 or more invalid iterations were excluded from the analysis. In addition, for the analysis we included only points inside the inner triangular region with vertices (f,f),(1-2f,f),(1/2,√3/2-f), where f=300/2000. In total this resulted in 99,189 out of 2,418,284 tapped responses being excluded from the main experiment (4.1 %).

For online experiments, before starting the experiment, participants completed a short recording test to detect hardware and software that did not meet the technical requirements of the experiment, such as malfunctioning speakers or microphones. The recording test contained three trials. If the first trial failed (for example, this could occur if the participant forgot to unplug their headphones) we reminded participants that they needed to unplug any headphones. If, despite this reminder, the test recording failed in one of the two subsequent trials, the participant was excluded from the experiment (see Methods for further details). Some participants also abandoned the task during the recording test (for example because their internet connection stopped working). In total 747 out of 1303 participants were excluded from the remainder of the experiment for one of these two reasons.

Next, participants performed a practice phase to acquaint themselves with the main tapping task and to further test technical compatibility of their browser and computer. Following the practice phase, we provided feedback to participants based on their recording quality and tapping performance. Prior to the main experiment, we asked participants to adjust their tapping based on our feedback. During the analysis of the experiment, we used two criteria to exclude trials. First, we excluded all trials for which the recording of the stimuli (recorded in the online experiment along with the responses) was determined to be inaccurate (see Methods for details). Second, we excluded all trials where the percentage of detected taps (i.e., the number of detected tapping onsets out of the total number of stimulus onsets) was less than 50% or more than 200%. Note that none of these criteria excluded trials based on actual accuracy in replicating the target rhythm, but only based on whether the signal could be correctly recorded and processed, and whether participants produced a minimally and maximally acceptable number of tapping responses. An additional 358

participants were excluded on this basis. Finally, we excluded participants who abandoned the experiment prior to its completion or who did not complete the full demographic questionnaire that we administered at the end of the experiment (67 additional participants were excluded on this basis). In total 131 participants completed the full experiment and were analyzed.

More details of the online experiment are reported in the "Online measurement of tapped responses" section of the methods. Validation of the online method (comparing the performance of online experiments and in-lab experiments), including justification of the exclusion criteria, is provided in the cited paper Anglada-Tort et al. (2021).

Non-participation

With in-person experiments, very few participants (typically 1-2 per group) had their experimental session terminated based on an inability to tap, presumably resulting from some motor dysfunction or inability to follow instructions.

Randomization

N/A. Participants were not allocated into groups. Participants were analyzed in groups determined by their location and musical experience (described in the section "Criteria for group selection" in the Methods). All participants completed the same experiment.

Reporting for specific materials, systems and methods

We require information from authors about some types of materials, experimental systems and methods used in many studies. Here, indicate whether each material, system or method listed is relevant to your study. If you are not sure if a list item applies to your research, read the appropriate section before selecting a response.

Materials & experimental systems

n/a	Involved in the study	
☒	☐	Antibodies
☒	☐	Eukaryotic cell lines
☒	☐	Palaeontology and archaeology
☒	☐	Animals and other organisms
☐	☒	Human research participants
☒	☐	Clinical data
☒	☐	Dual use research of concern

Methods

n/a	Involved in the study	
☒	☐	ChIP-seq
☒	☐	Flow cytometry
☒	☐	MRI-based neuroimaging

Human research participants

Policy information about studies involving human research participants

Population characteristics

See above.

Recruitment

Recruitment strategies varied depending on the location; see Methods for details in each case. We typically tried to test every potential participant available at a testing site. However, because participation was voluntary, participants who were less confident in their tapping skills may have been less likely to participate.

Ethics oversight

Informed consent was obtained using the following approved protocols: Ethics Council of the Max Planck Society (2017_12, 2020_05, 2020_11), Columbia University IRB-AAAR3726, University of Western Ontario Health Science Research Ethics Board 108477, Korea Advanced Institute of Science and Technology (KAIST IRB -KH2017-15), Chinese University of Hong Kong SBRE-19-695, BCPHS-12301 (Brandeis), Durham University Music Department Ethics Committee (February 2018), Boğaziçi University Ethical Board for Human Research SBB-EAK 2017/1, Committee on the Use of Humans as Experimental Subjects at MIT (COUHES) Protocol Number 1209005242R004. Consent to publish images was obtained from participants or music ensembles.

Note that full information on the approval of the study protocol must also be provided in the manuscript.
